# Synthesis of novel sulfide-based cyclic peptidomimetic analogues to solonamides

**DOI:** 10.3762/bjoc.15.247

**Published:** 2019-10-25

**Authors:** José Brango-Vanegas, Luan A Martinho, Lucinda J Bessa, Andreanne G Vasconcelos, Alexandra Plácido, Alex L Pereira, José R S A Leite, Angelo H L Machado

**Affiliations:** 1 Instituto de Química, Universidade de Brasília, Campus Universitário Darcy Ribeiro, 70910-900, Asa Norte, Brasília DF, Brasil; 2 LAQV/REQUIMTE, Departamento de Química e Bíoquímica, Faculdade de Ciências da Universidade do Porto, 4169007, Porto, Portugal; 3 Área de Morfologia, Faculdade de Medicina, Universidade de Brasília, 70910-900, Brasília, DF, Brasil; 4 Glial Cell Biology Lab, Instituto de Investigação e Inovação em Saúde, i3S, Universidade do Porto, 420013, Porto, Portugal; 5 Bioprospectum, Lda, UPTEC, 4200135, Porto, Portugal; 6 Campus de Ceilândia, Universidade de Brasília, Centro Metropolitano, 72220-275, Ceilândia Sul, Ceilândia, DF, Brazil

**Keywords:** antivirulence drug, bacteria, macrocyclization, pathoblocker, quorum quenching

## Abstract

Eight new sulfide-based cyclic peptidomimetic analogues of solonamides A and B have been synthesized via solid-phase peptide synthesis and S_N_2’ reaction on a Morita–Baylis–Hillman (MBH) residue introduced at the *N*-terminal of a tetrapeptide. This last step takes advantage of the electrophilic feature of the MBH residue and represents a new cyclization strategy occurring. The analogues were prepared in moderate overall yields and did not show toxic effects on *Staphylococcus aureus* growth and were not toxic to human fibroblasts. Two of them inhibited the hemolytic activity of *S. aureus*, suggesting an interfering action in the bacterial quorum sensing similar to the one already reported for solonamides.

## Introduction

**Figure 1:**
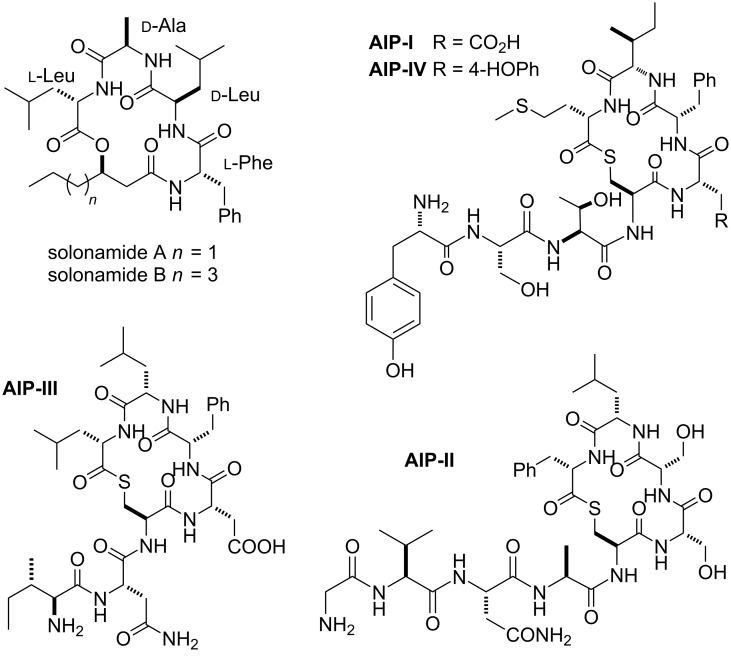
Chemical structure of solonamides and autoinducing peptides (AIP).

The cyclodepsipeptides called solonamides A and B are natural molecules extracted from the marine bacterium *Photobacterium halotolerans* [1–2] (Figure 1). They are able to prevent the expression of *Staphylococcus aureus* virulence factors such as α-hemolysin and phenol-soluble modulins without affecting the bacterial growth [3]. Particularly, solonamide B and its analogues revealed no detectable toxicity against erythrocytes or human neutrophils [3–4].

*Staphylococcus aureus* is an opportunistic Gram-positive bacterium found in human commensal microbiota. Once epithelial barriers or immune systems become compromised, *S. aureus* can cause skin and soft tissue infections besides severe invasive diseases such as endocarditis, pneumonia, and septicemia [5–7]. In particular, methicillin-resistant *S. aureus* (MRSA) is considered an endemic cause of nosocomial infections and has spread into the community and livestock animals as well [8]. Expression of many *S. aureus* virulence factors is controlled by a sophisticated intercellular chemical signalling pathway named quorum-sensing (QS) circuit Agr (accessory gene regulator) [8–11]. Four native thiolactonic cyclopeptides, named autoinducing peptides (AIPs, Figure 1), were found to be the chemical signals for the QS circuit Agr. Their chemical structures are remarkably alike to solonamides, and the synthesis of new molecules structurally related to these natural peptidomimetics has been used as a promising strategy for the attenuation of bacterial virulence in strains of *S. aureus* [12–15].

**Scheme 1:**
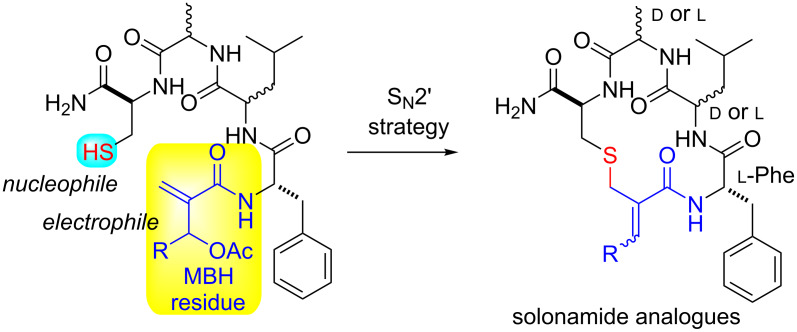
Macrocyclization strategy based on S_N_2’.

Herein, we report the synthesis of new sulfide-based cyclic peptidomimetics through the allylic nucleophilic substitution (S_N_2’) of cysteine sulfhydryl side chain to electrophilic Cβ of an *O*-acetylated Morita–Baylis–Hillman (MBH) adduct residue (Scheme 1). Despite reports describing the use of amino acid residues with nucleophilic side chains to prompt the macrocyclization of peptides and their mimetics, to the best of our knowledge, this is the first report on the participation of MBH residues as electrophilic sites to allow an S_N_2’-based macrocyclization of peptidomimetics [16–30]. Two of these new compounds were able to interfere with the hemolytic activity of *S. aureus*, and since the hemolysin expression is activated by *S. aureus* QS, we can suppose that the reported activity may be related to the inhibition of this bacterial communication system.

## Results and Discussion

### Rational design and synthesis of the solonamide analogues

The rational design of our solonamide analogues was based on the conservation of the 16-membered macrocyclic scaffold and the apolar tripeptidyl moiety found in the solonamides. Both features are important to guarantee the interference with *S. aureus* QS [12–15]. The ester linkage of the lactone core was substituted by the sulfide group. Cyclic thioether peptides have been found in the chemical skeletons of natural products and synthetic ones that display a wide variety of activities, including antibiotics [31], vascular cell adhesion molecule-1 antagonists [32] and anticardiolipin antibodies [33–34].

**Scheme 2:**
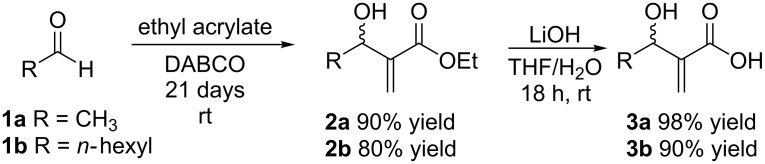
Chemical synthesis of the MBH adducts **2** and their carboxylic acids **3**.

Two MBH adducts (**2**) (R = Me, heptyl) and their respective carboxylic acids **3** were obtained in good yields based on previously reported procedures (Scheme 2) [35–36].

**Scheme 3:**
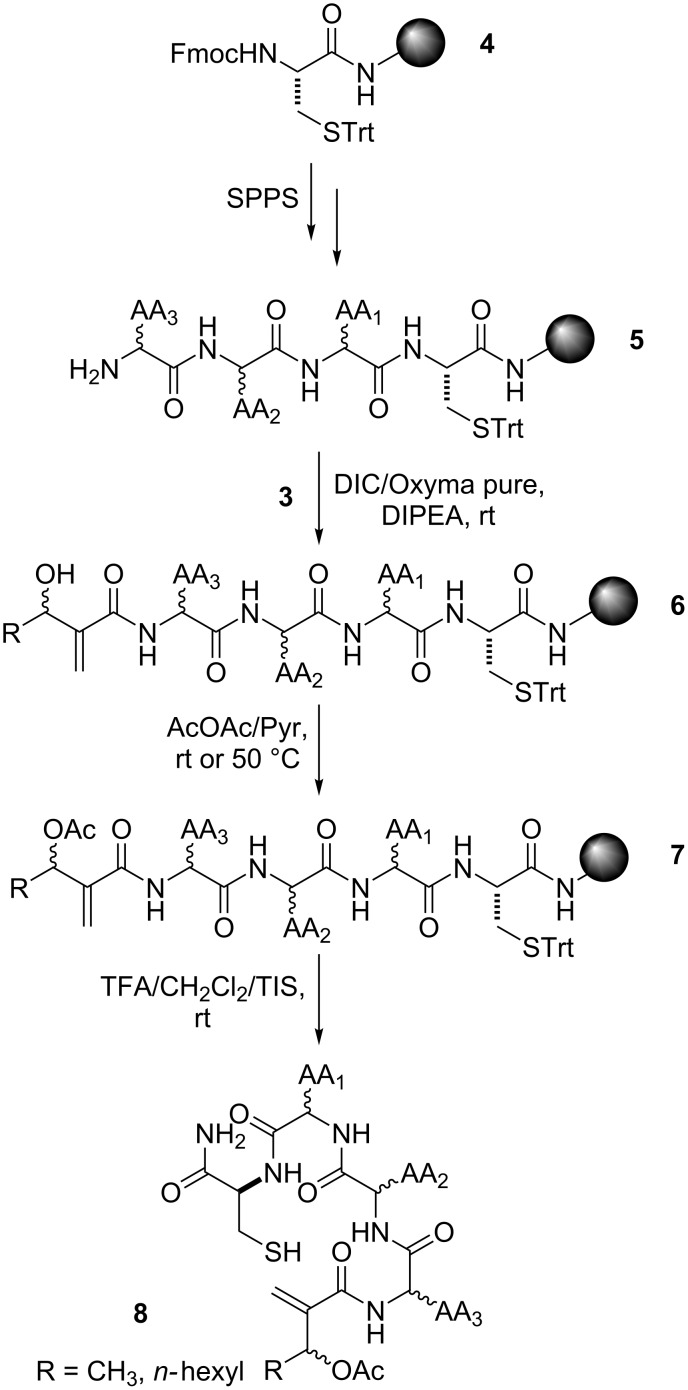
Chemical synthesis of the linear peptidomimetics **8**.

Starting from Rink Amide AM resin-bound orthogonally protected Fmoc-Cys-(Trt) **4**, solonamide analogues were synthesized following stepwise Fmoc deprotection and standard repetitive Fmoc-amino acid couplings yielding the linear resin-bound tetrapeptides **5** (Scheme 3) [37–38]. The MBH acids **3** were coupled to the free amine at the *N*-terminal of **5** to afford the resin-bound linear peptidomimetics **6**, which subsequently had the hydroxy group of the MBH residue acylated with acetic anhydride to yield **7**. Despite the good results for the acetylation of peptides **6** with R = Me at room temperature, the reaction of the ones with R = *n-*hexyl needed to be conducted under heat conditions (50 °C). Acidic treatment of the acetates **7** with trifluoroacetic acid: triisopropylsilane decoupling cocktail afforded the resin-free linear peptidomimetics **8**.

**Scheme 4:**
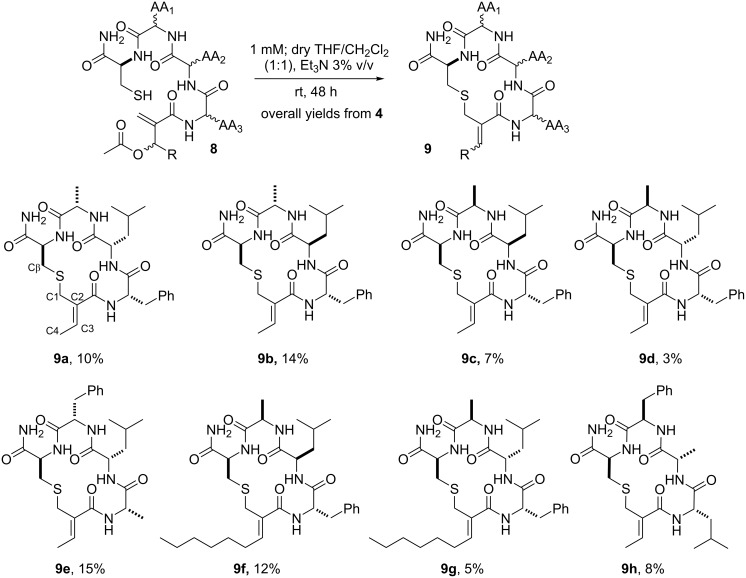
Macrocyclization strategy based on S_N_2’ reaction to affords the solonamide analogues **9** and their overall yields based on the initial resin’s molarity.

The S_N_2’ macrocyclization step was performed immediately after the cleavage procedure to avoid the oxidative disulfide dimerization observed when preparative HPLC purification of the resin-free linear peptidomimetics **8** was tried. Thus, a 1 mM solution of peptides **8** in dry THF/CH_2_Cl_2_ (1:1) containing Et_3_N was vigorously stirred for 48 h at room temperature (Scheme 4) as previously described in a similar procedure for the intermolecular S_N_2’ reaction between thiophenol and *O*-acetylated MBH adducts [39]. The solonamide analogues **9** were obtained after preparative HPLC purification and lyophilization. The overall yield for this 11-step synthesis ranged from 7% to 15% for almost all solonamide analogues **9**, based on the initial resin’s molarity. The exception relays on those containing ᴅ-Ala and ʟ*-*Leu amino acid residues sequentially attached to an ʟ-Cys residue, which were obtained in yields lower than 5%. We ascribed this result to the steric strains imposed by the spatial accommodation of these amino acid residue side chains on the resin-free linear peptidomimetics **8**, which seems to disfavour the approximation of the nucleophilic sulfhydryl group to the electrophilic MBH residue.

### Spectral characterization of the chemical structures of the solonamide analogues **9**

The compounds were characterized by one- and two-dimensional NMR spectroscopy, infrared spectroscopy (IR) and mass spectrometry. The high-resolution MS/MS analysis allowed the observation of a fragmentation pattern that does not coincide with the one found in linear peptides, where we would observe the loss of the ʟ-Cys residue. In addition to the sequential loss of ammonia (17 amu) and CO (28 amu) to yield the iminium [M − NH_3_ − CO]^+^ (Schemes S4–S11, Supporting Information File 1), as expected for the C-terminal carboxamide, we observed three ions derived from the breaking of two amide bonds, starting from the opening of the macrocycle by the loss of one, two or three amino acid residues as neutral fragments. This fragmentation pattern agrees with the one expected for cyclic peptides [40]. Noteworthy, the detected ions **A**–**C** always showed the –SCH_2_CH=NH moiety, confirming the formation of the sulfide group (Schemes S4–S11, Supporting Information File 1).

The NMR experiments also confirmed the macrocyclic structure (Supporting Information File 1). The ^1^H NMR spectra of compounds **9** had the characteristic signal in δ 3.2–3.6 ppm, resonating as a dd, assigned to C1 methylene protons (Scheme 4); this signal only changes in the multiplicity for compounds **9g** and **9h** that is presented as a broad singlet. Additionally, we observed another signal around δ 5.8–6.5 ppm, that depending on the compound can be a q or t, integrating for one hydrogen, assigned to C3 methine hydrogen. The ^1^H,^13^C-HMBC spectra allows us to observe an important strong correlation between the signals assigned to the protons on C1 and the carbon Cβ of the cysteine, which strongly suggest the formation of the new C–S bond. The configuration of the double bond could be assigned as *Z* for all compounds due to the ^1^H,^1^H-NOESY correlations between the C3 hydrogen and the NH hydrogen of the amino acid residue attached to the adduct residue.

The IR spectra of analogues **9** were quite similar (Supporting Information File 1). Three main absorption bands could be readily observed around 3280, 1650 and 1520 cm^−1^. The first one was assigned to the stretch for N–H bonds of the peptide linkage. The stretch for the lactam and lactone C=O bonds gives rise to the broad absorption close to 1650 cm^−1^. The lowering on the wavenumber values for the lactone C=O stretch was also observed for bands assigned to the C=C bonds as consequence of their conjugation.

### Evaluation of the growth inhibition and hemolytic activity of *S. aureus* for the solonamide analogues

Initially, the antibacterial activity of all analogues **9** was tested by determining the minimum inhibitory concentration against two antibiotic-susceptible reference strains of *S. aureus*, *S. aureus* ATCC 25923 and *S. aureus* ATCC 29213 (see Supporting Information File 1, assay 1) [41]. Two-fold serial dilutions were performed, allowing to test several concentrations within the range of 300–0.3 µM. None of the compounds presented antibacterial activity against *S. aureus*, since no MIC value could be obtained in the range of concentrations tested (MIC > 300 µM).

**Figure 2:**
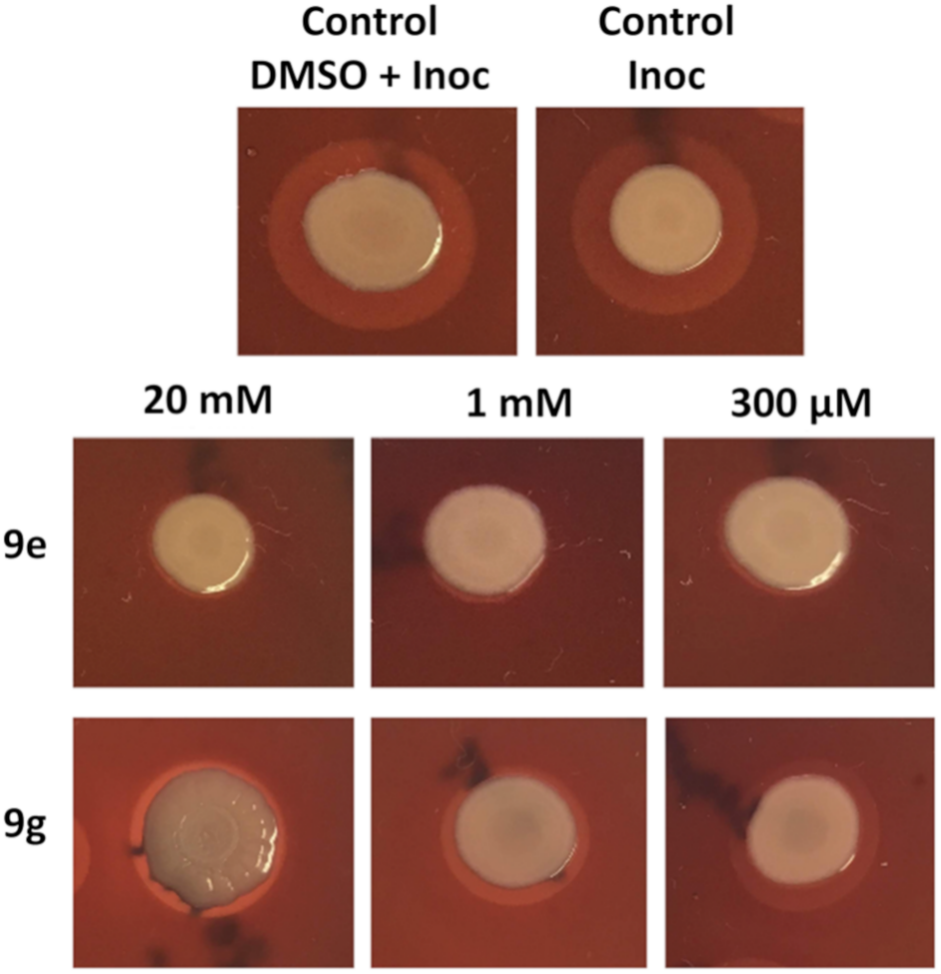
Effect of compounds **9e** and **9g** at three concentrations on the hemolysis production by *S. aureus* ATCC 25923 (Assay 2, Inoc = inoculum). Regarding compound **9e**, no hemolysis halo was observed around the bacterial spot.

Subsequently, a screening assay was carried out to evaluate the effect of these compounds on the hemolysis of defibrinated sheep blood promoted by *S. aureus* ATCC 25923, a strain that produces hemolysins under the control of QS (see Supporting Information File 1, assay 2) [42]. Among all compounds, **9e** and **9g** showed the best results, inhibiting the hemolytic activity of *S. aureus* at lower concentrations (Table 1 and Figure 2). Analogues **9e** and **9g** were then tested at lower concentrations (300–5 µM), and **9e** was able to hamper the hemolysis by the strain at 10 µM (see Supporting Information File 1, Table S1).

**Table 1:** Halos of hemolysis or inhibition of hemolysis of *S. aureus* ATCC 25923 on sheep blood agar plates (Assay 2).^a^

Analogue	20 mM	1 mM	200 μM

**9a**	+	+	+
**9b**	+	+	+
**9c**	+	+	+
**9d**	+	+	+
**9e**	–	–	–
**9f**	+	+	+
**9g**	–	+*	+*
**9h**	–	+	+

^a^(+): Hemolysis halo; (–): no hemolysis halo; *visibly smaller hemolysis halo when compared to controls.

**Figure 3:**
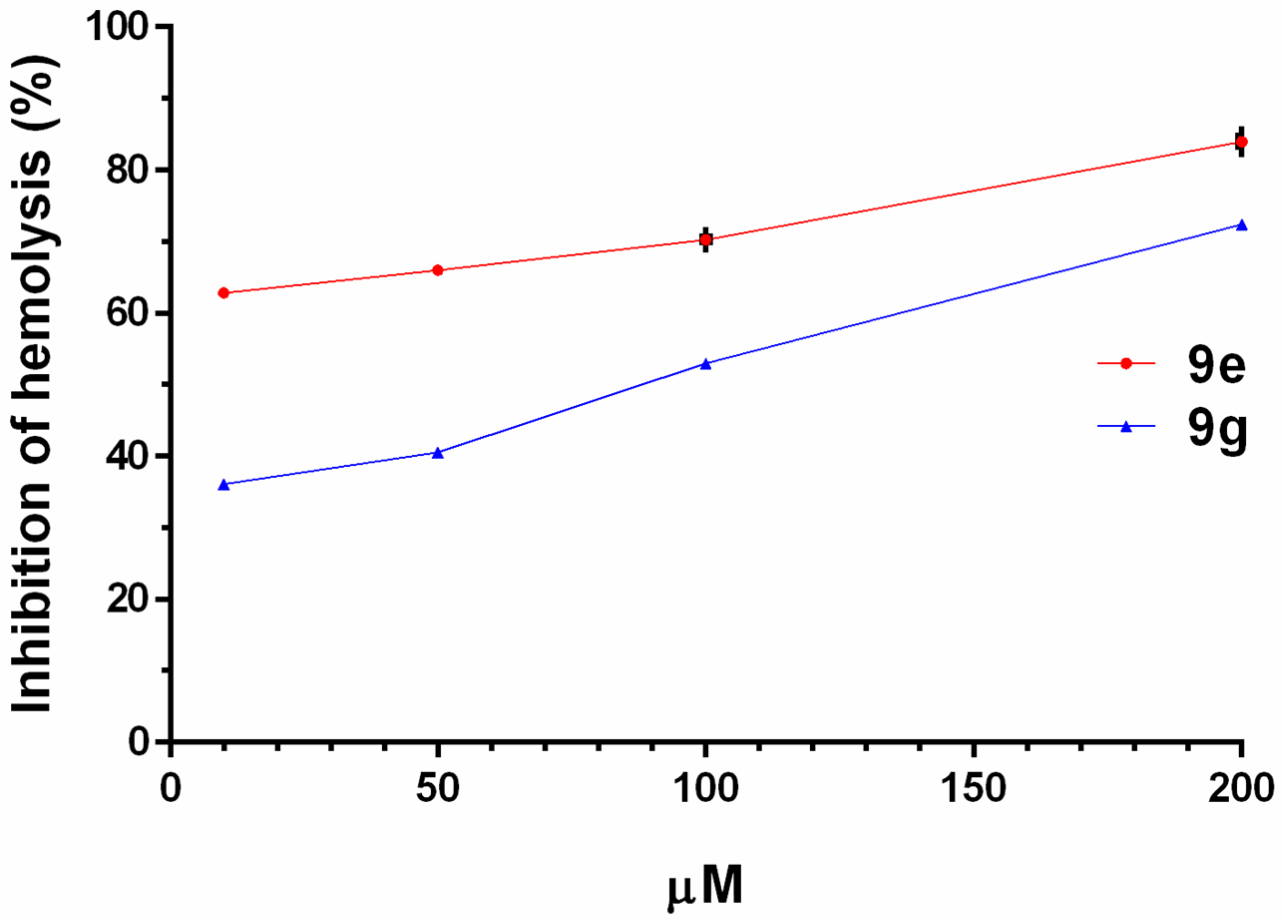
Inhibition of hemolysis (%) on human red blood cells caused by *S. aureus* ATCC 25923 after being in presence of several concentrations of compounds **9e** and **9g** (assay 3). The hemolysis is scored in % of hemolysis relative to the control (hemolysis caused by *S. aureus* ATCC 25923 in absence of any compound; only DMSO), which was set to 100%. Two independent experiments were performed in duplicate. Error bars represent standard deviations.

The antihemolytic activity of **9e** and **9g** was also tested in a quantitative microdilution assay using human red blood cells (see Supporting Information File 1, assay 3) [43]. Analogue **9e** showed better capacity to inhibit the hemolysis than **9g** at the same concentration (Figure 3). At concentrations of 10 µM and 200 µM of **9e**, the hemolysis production by *S. aureus* was inhibited by 63% and 84%, respectively.

**Figure 4:**
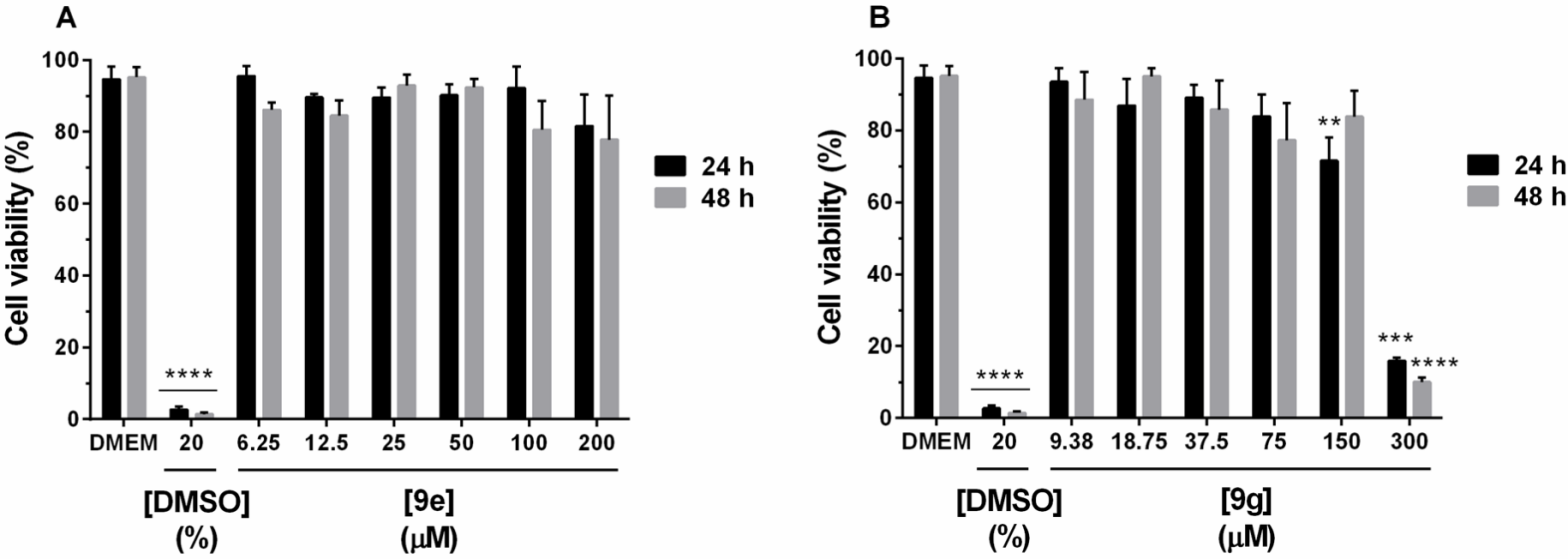
Cell viability of human fibroblast exposed to compounds **9e** (A) and **9g** (B) for 24 and 48 hours. The values are expressed as mean ± SEM. ****p < 0.0001, **p < 0.005 and ***p < 0.001 versus DMEM control group.

Also, a cell viability assay on human fibroblasts (Detroit 551 cell line) was performed for compounds **9e** and **9g** in order to observe their eventual cytotoxicity to normal cells (see Supporting Information File 1, assay 4). The concentration range tested was 6.25 to 200 µM for **9e** and 9.38 to 300 µM for **9g,** chosen based on the optimal dose observed in the hemolysis inhibition assay. We observed that these two compounds did not affect the fibroblast viability in the concentrations tested (Figure 4). Furthermore, no statistically significant difference was observed between exposition times of 24 and 48 h. These data suggest that **9e** and **9g** are not toxic to normal cells within the tested experimental conditions.

Solonamide B has been shown to inhibit *agr*-controlled phenotypes in *S. aureus* (including hemolysis) at concentrations ranging between 8.5 µM and 17 µM [3]. Similar inhibitory concentrations (10 to 20 µM) were displayed by our analogues, **9e** and **9g**, with the advantage that sulfide linkage is less prompted to hydrolysis than the ester found at solonamides [44–45]*.* Comparing the chemical structure of analogues **9e**–**g** and **9h** to the previously reported analogues of solonamides and *S. aureus* AIPs, some observations can be made: (1) **9e** is the only one among all the analogues derived from the acetaldehyde adduct that, regardless of the configuration of the stereogenic centres, has the same amino acid residue sequence found in solonamides; (2) despite the close similarity on the amino acid residue sequence displayed by **9e** and **9h**, the stereochemistry of the Phe residue is detrimental to the antihemolytic activity; (3) in a similar sense, for **9f** and **9g**, diastereosiomers on the Ile residue, the L-configuration on this residue is necessary to the observed hemolysis inhibition by *S. aureus*.

## Conclusion

A new, effective and simple cyclization strategy using MBH adducts for the preparation of eight new sulfide-based cyclic peptidomimetics structurally similar to solonamides was established. Two of these new compounds had the capacity to inhibit the hemolytic activity of *S. aureus* ATCC 25923 without affecting its growth, at very low concentrations, namely 10 μM, and were not cytotoxic to human fibroblasts at the same concentration. Due to the well-known relationship of the α-hemolysins expression with the accessory gene regulator (*agr*) of the *S. aureus*, we can suggest that these two peptidomimetics may exert an effect on *S. aureus* quorum sensing. To evaluate this hypothesis, we are now rationally designing new solonamide analogues, based on the S_N_2’ strategy reported here, to be tested for AgrC-inhibitory activity.

## Supporting Information

File 1 Detailed synthetic procedures, biological assay procedures and copies of NMR and MS spectra of all compounds.

## References

[1] Mansson M, Nielsen A, Kjærulff L, Gotfredsen C H, Wietz M, Ingmer H, Gram L, Larsen T O (2011). Mar Drugs.

[2] Kitir B, Baldry M, Ingmer H, Olsen C A (2014). Tetrahedron.

[3] Nielsen A, Månsson M, Bojer M S, Gram L, Larsen T O, Novick R P, Frees D, Frøkiær H, Ingmer H (2014). PLoS One.

[4] Baldry M, Kitir B, Frøkiær H, Christensen S B, Taverne N, Meijerink M, Franzyk H, Olsen C A, Wells J M, Ingmer H (2016). PLoS One.

[5] Broderick A H, Stacy D M, Tal-Gan Y, Kratochvil M J, Blackwell H E, Lynn D M (2014). Adv Healthcare Mater.

[6] Grundmann H, Aires-de-Sousa M, Boyce J, Tiemersma E (2006). Lancet.

[7] (2018). European Centre for Disease Prevention and Control. Surveillance of antimicrobial resistance in Europe – Annual report of the European Antimicrobial Resistance Surveillance Network (EARS-Net) 2017.

[8] Lina G, Jarraud S, Ji G, Greenland T, Pedraza A, Etienne J, Novick R P, Vandenesch F (1998). Mol Microbiol.

[9] Novick R P, Geisinger E (2008). Annu Rev Genet.

[10] Greenberg E P (2003). J Clin Invest.

[11] Camilli A, Bassler B L (2006). Science.

[12] Tal-Gan Y, Stacy D M, Foegen M K, Koenig D W, Blackwell H E (2013). J Am Chem Soc.

[13] Tal-Gan Y, Ivancic M, Cornilescu G, Cornilescu C C, Blackwell H E (2013). J Am Chem Soc.

[14] Tal-Gan Y, Ivancic M, Cornilescu G, Blackwell H E (2016). Org Biomol Chem.

[15] Hansen A M, Peng P, Baldry M, Perez-Gassol I, Christensen S B, Vinther J M O, Ingmer H, Franzyk H (2018). Eur J Med Chem.

[16] Feng Y, Pattarawarapan M, Wang Z, Burgess K (1999). Org Lett.

[17] Feng Y, Burgess K (2000). Biotechnol Bioeng.

[18] Park C, Burgess K (2001). J Comb Chem.

[19] Burgess K (2001). Acc Chem Res.

[20] Lindman S, Lindeberg G, Gogoll A, Nyberg F, Karlén A, Hallberg A (2001). Bioorg Med Chem.

[21] Johannesson P, Lindeberg G, Johansson A, Nikiforovich G V, Gogoll A, Synnergren B, Le Grèves M, Nyberg F, Karlén A, Hallberg A (2002). J Med Chem.

[22] Giulianotti M, Nefzi A (2003). Tetrahedron Lett.

[23] Reyes S J, Burgess K (2005). Tetrahedron: Asymmetry.

[24] Grieco P, Cai M, Liu L, Mayorov A, Chandler K, Trivedi D, Lin G, Campiglia P, Novellino E, Hruby V J (2008). J Med Chem.

[25] Derbel S, Ghedira K, Nefzi A (2010). Tetrahedron Lett.

[26] Nefzi A, Arutyunyan S, Fenwick J E (2010). J Org Chem.

[27] Nefzi A, Fenwick J E (2011). Tetrahedron Lett.

[28] Wu Z-M, Liu S-Z, Cheng X-Z, Ding W-Z, Zhu T, Chen B (2016). Chin Chem Lett.

[29] Nishihara T, Kitada H, Fujiwara D, Fujii I (2016). Biopolymers.

[30] Sutherland B P, El-Zaatari B M, Halaszynski N I, French J M, Bai S, Kloxin C J (2018). Bioconjugate Chem.

[31] Jung G (1991). Angew Chem, Int Ed Engl.

[32] Fotouhi N, Joshi P, Tilley J W, Rowan K, Schwinge V, Wolitzky B (2000). Bioorg Med Chem Lett.

[33] Jones D S, Gamino C A, Randow M E, Victoria E J, Yu L, Coutts S M (1998). Tetrahedron Lett.

[34] Roberts K D, Lambert J N, Ede N J, Bray A M (1998). Tetrahedron Lett.

[35] Silva V S, Tolentino T A, Rodrigues T C A F, Santos F F M, Machado D F S, Silva W A, de Oliveira H C B, Machado A H L (2019). Org Biomol Chem.

[36] Mateus C R, Feltrin M P, Costa A M, Coelho F, Almeida W P (2001). Tetrahedron.

[37] Kim Y-W, Grossmann T N, Verdine G L (2011). Nat Protoc.

[38] (2019). AAPPTEC. Practical Guide to Solid Phase Peptide Synthesis.

[39] Kamimura A, Morita R, Matsuura K, Mitsudera H, Shirai M (2003). Tetrahedron.

[40] Liu W-T, Ng J, Meluzzi D, Bandeira N, Gutierrez M, Simmons T L, Schultz A W, Linington R G, Moore B S, Gerwick W H (2009). Anal Chem (Washington, DC, U S).

[41] (2019). Staphylococcus aureus subsp. Aureus Rosenbach (ATCC® 29213™).

[42] Nakayama J, Uemura Y, Nishiguchi K, Yoshimura N, Igarashi Y, Sonomoto K (2009). Antimicrob Agents Chemother.

[43] Sakoulas G, Eliopoulos G M, Moellering R C, Wennersten C, Venkataraman L, Novick R P, Gold H S (2002). Antimicrob Agents Chemother.

[44] Webster A M, Cobb S L (2018). Chem – Eur J.

[45] Sivanathan S, Scherkenbeck J (2014). Molecules.

