# Supplementary material for: Synthesis of novel sulfide-based cyclic peptidomimetic analogues to solonamides
Source: Beilstein J Org Chem. 2019 Oct 25;15:2544–51. doi: 10.3762/bjoc.15.247 (PMC6839570; doi:10.3762/bjoc.15.247)
Supplement: File 1 — Detailed synthetic procedures, biological assay procedures and copies of NMR and MS spectra of all compounds. [file Beilstein_J_Org_Chem-15-2544-s001.pdf]

BEILSTEIN JOURNAL OF ORGANIC CHEMISTRY

Supporting Information

for

Synthesis of novel sulfide-based cyclic peptidomimetic analogues to solonamides

José Brango-Vanegas, Luan A. Martinho, Lucinda J. Bessa, Andreanne G. Vasconcelos, Alexandra Plácido, Alex L. Pereira, José R. S. A. Leite and Angelo H. L. Machado

Beilstein J. Org. Chem. **2019,** *15,* 2544–2551. doi:10.3762/bjoc.15.247

Detailed synthetic procedures, biological assay procedures and copies of NMR and MS spectra of all compounds

License and Terms: This is a supporting information file under the terms of the Creative Commons Attribution License (http://creativecommons.org/licenses/by/4.0). Please note that the reuse, redistribution and reproduction in particular requires that the authors and source are credited.
The license is subject to the *Beilstein Journal of Organic Chemistry* terms and conditions: (https://www.beilstein-journals.org/bjoc)

Table of Contents

**1. Abbreviations**	S2
**2. Experimental materials and methods**	S3
**2.1 Reagents and analysis**	S3
**2.2 Activity assays on *S. aureus* and on human fibroblasts**	S4
**3. Synthetic procedures and analytical data**	S5
**3.1 Synthetic procedure of Morita–Baylis–Hillman adducts (2)**	S5
**3.2 Synthetic procedure of hydrolysis of Morita–Baylis–Hillman adducts (3)**	S6
**3.3 Procedure of solid-phase peptide synthesis – SPPS**	S6
**3.4 Synthetic procedure for the cyclization reaction**	S7
**3.5 Analytical data of the cyclopeptides**	S8
**4. NMR and MS data**	S16
**References**	S66

1. Abbreviations

SPPS: Solid-phase synthesis of peptides
THF: Tetrahydrofuran
HBTU: *N,N,N′,N′*-Tetramethyl-*O*-(1*H*-benzotriazol-1-yl)uronium hexafluorophosphate
HATU: *N*-[(Dimethylamino)-1*H*-1,2,3-triazolo-[4,5-*b*]pyridin-1-ylmethylene]-*N*-methylmethanaminium hexafluorophosphate *N*-oxide
6-Cl-HoBt: 6-Chloro-1-hydroxybenzotriazole
DIC: *N,N′*-Diisopropylcarbodiimide
Oxyma Pure: Ethyl (hydroxyimino)cyanoacetate
DIPEA: *N*,*N*-Diisopropylethylamine
MHB-II: Muller-Hilton Broth II medium
DMSO: Dimethyl sulfoxide
CFU: Colony forming unit
hRBCs: Human red blood cells
NMR: Nuclear magnetic resonance spectroscopy
DAD: Diode-array detector
TFA: Trifluoracetic acid
APT: Attached proton test 1DC NMR experiment
DABCO: 1,4-diazabicyclo[2.2.2]octane
TEM: Temperature
GS1: Ion source gas 1
GS2: Ion source gas 2
CUR: Curtain gas
ISVF: Ion spray voltage floating
DP: Declustering potential
EDTA: Ethylenediamine tetraacetic acid
DCM: Dichloromethane
TMS: Tetramethylsilane
HRMS: High-resolution mass spectrometry
TOF: Time-of-flight mass analyzers
ESI: Electrospray ionization
Fmoc: Fluorenylmethyloxycarbonyl protecting group

2. Experimental materials and methods

2.1 Reagents and analysis

All organic solvents and liquid reagents used in the SPPS were available from Sigma-Aldrich, Scharlau, J.T. Baker and Merck, and were used without further purification. Some solvents were dried before used; to dry Et_3N $CaH_2$was used and to dry THF it was used sodium metal in the presence of benzophenone.
Rink Amide AM resin (100–200 mesh) with 0.7 mmol/g loading of Novabiochem® (EMD Millipore Corporation - Merck) was used for SPPS. Fmoc-Ala-OH, Fmoc-Leu-OH, Fmoc-Phe-OH, were obtained from Aldrich® (Sigma-Aldrich); Fmoc-(D)-Ala-OH, Fmoc-(D)-Leu from 3PBioSystem and Fmoc-Cys(Trt)-OH from Novabiochem (EMD Millipore Corporation - Merck). As coupling agents for the formation of the peptide bond, HBTU or HATU, from Fluka® (Sigma-Aldrich), together with 6-Cl-HOBt from Acros® as racemization agent were used. DIC (Fluka®, Sigma-Aldrich) with Oxyma Pure® (Merck) were used for coupling to MBH adduct acid. DIPEA was used as bases.
One and two-dimensional nuclear magnetic resonance spectra were recorded on a Bruker Ascend 600 spectrometers operating to 14 T (600 MHz for ^{1}H-NMR). The APT and ^{13}C NMR spectra were acquired on a Varian Oxford YH300 on 7.04 T (75 MHz for ^{13}C NMR). On ^{1}H NMR the TMS was used as internal referential, chemical shifts were reported as δ (ppm) values and coupling constants were in hertz (Hz). Chemical shifts for ^{13}C NMR were reported in ppm relative to the solvent peak. Data were analyzed using MestReNova 6.0.2.
The analytical and semi-preparative HPLC experiments were done on a Shimadzu apparatus, equipped with two independent pumps LC-20AR and a DAD detector SPD-M20A. The selected wavelength was 216 nm. A gradient of two solvents was used; solvent A was a 0.2% solution of TFA in H_2O and solvent B was CH_3CN with the same concentration of acid. HPLC profiles in the analytical mode were obtained using a Shimadzu Shim-pack VP-ODS (C18) column (250 mm(L) × 4.6 mm(d.i), 5 µm). The run started with a constant flow of 5% B for 5 min; after a gradient by 40 min, starting in 5% to 100% B was employed; from 45 min to 52 min a constant flow of 100% B, and on 3 min the concentration of B decreases from 100% to 5% to establishing the original condition. Then 5% B for 5 min more. The flow used was 1.0 mL/min. Purification of the cyclopeptides compounds in the semi-preparative mode was done using a Vydac C18 column (250 mm(L) × 10 mm(di), 5 µm, 300 Å). The run was started with 25% of solution B for 2 min, then a gradient in which the concentration of solvent B increased to 54% for 54 min, after this time the solvent B followed a second gradient to 100% for two min and a isocratic elution for 5 min more was followed, after the original elution condition (25% of B) was returned in 2 min. The flow rate was 2.5 mL/min.
HRMS-ESI-TOF data were acquired on a Triple TOF 5600-SCIEX by injection analysis on flow using an Eksigent Ultra LC 100 - SCIEX chromatograph adjusted at a flow rate of 0.3 mL/min. A DuoSpray Ion Source (ESI) was used and the MS spectra were acquired in the positive mode, using external calibration, in the range of 100 - 1000 Da. The acquisition parameters were: TEM 450, GS1 45, CUR 25, GS2 50, ISVF 5500 and DP 80. Data were analyzed using mMass 5.5.0 (http://www.mmass.org).[1]
Infrared spectra were obtained on the Jasco FT/IR-4100 spectrophotometer operating by attenuated total reflectance (ATR) mode. IR-ATR were expressed as transmittance (% T) versus wavenumber (cm^{-1}).

2.2 Activity assays on *S. aureus* and on human fibroblasts

Assay 1. Determination of the minimum inhibitory concentration (MIC). The antibacterial activity of each cyclopeptide was assessed against two *S. aureus* reference strains, *S. aureus* ATCC 25923 and *S. aureus* ATCC 29213.The microdilution test was performed according to protocol M07-A of Clinical and Laboratory Standards Institute (CLSI),[2] using the MHB-II medium (Sigma-Aldrich). A stock solution in DMSO of 20 mM of each analogue **9** was used to prepare two-fold dilutions to achieve the tested concentration range between 300 and 0.3 μM. DMSO was used as control to ensure that it did not interfere with the bacterial growth. The microplates were inoculated with 5×10^5 CFU/mL of the strain and incubated at 37 °C for 24 h. After that time, the MIC, defined as the lowest concentration that completely inhibited the growth of bacteria, was obtained as detected by the naked eye.

Assay 2. Assessment of the hemolytic activity of *S. aureus* ATCC 25923 on sheep blood agar. The methodology used was adapted from Nakayama *et al*. 2009.[3] Briefly, 5 µL of three concentrations of each cyclopeptide in DMSO (300 μM, 1 mM and 20 mM) were spotted onto a sheep blood agar plate and let to dry. 5 µL of DMSO were used as solvent control. Then, on the top of each one of the previous drops, 1 µL of an inoculum of *S. aureus* ATCC 25923, previously prepared from fresh colonies and standardized to an optical density at 600 nm (OD_{600}) of 0.1 (approximately 1×10^8 CFU/mL), was added. A control spot of only 1 µL of the inoculum was also used to observe the hemolysis halo produced by the strain. Each plate was incubated at 37 °C for 20–24 h. The presence or absence of halos of hemolysis around the spotted bacteria were observed and photographed. Due to the results achieved, compounds **9e** and **9g** were then also tested at lower concentrations (5, 10, 50, 100 and 300 μM). Two independent experiments were performed, each in duplicate.

Tabel S1: Halos of hemolysis or inhibition of hemolysis of *S. aureus* ATCC 25923 on blood agar for compounds **9e** and **9g** at lower concentrations (Assay 2).[a]

Analogue	300 μM	100 μM	50 μM	10 μM	5 μM
**9e**	-	-	-	-	+*
**9g**	+*	+	nt	nt	nt

[a] (+): hemolysis halo; (-): no hemolysis halo; * visibly smaller hemolysis halo when compared to controls; nt: not tested.

Assay 3. Hemolysis assay using human red blood cells (hRBCs). Fresh colonies of *S. aureus* ATCC 25923 were used to prepare an inoculum with an OD_{600}=0.1. The compounds **9e** and **9g** were tested at different concentrations (10, 50, 100 and 200 μM). Briefly, 2 µL of solutions 100x concentrated (1 mM, 5 mM, 10 mM and 20 mM in DMSO) of compound **9e** and **9g**) were added to 198 µL of the prepared inoculum. Two positive controls were used, 2 µL of DMSO on 198 µL of inoculum and 200 µL of only inoculum. An incubation at 37 °C for 7 h happened, and after that, 100 µL of each bacterial suspension were directly added to 100 µL of an hRBC suspension of 2%. Previously, whole human blood was collected in EDTA (1.8 mg/mL), washed three times, and resuspended in PBS (pH 7.4) and then diluted to 2% in PBS. The mixtures of bacterial suspension plus hRBCs were incubated for 30 min at 37°C and then another 30 min at 4 °C.[4] Then, they were centrifuged at 1000 × g for 5 min and the absorbance (Abs) at 420 nm of the supernatant was read. Two independent experiments were performed, each in duplicate.

Assay 4. Cell viability assay on human fibroblasts. Human fibroblasts (Detroit 551 line) was purchased from Rio de Janeiro Cell Bank (BCRJ, Brazil). Cells were cultured in Dulbecco's Modified Eagle's Medium (DMEM, Vitrocell, Brazil) supplemented with 1% antibiotic solution (5,000 U/mL penicillin and 5 mg/mL streptomycin, LGC Biotecnologia, Brazil) and 10% heat inactivated fetal bovine serum (LGC Biotecnologia, Brazil) at 37 °C and 5% CO_2 in a humidified atmosphere. The cells were seeded into 96-well culture plate at a density of 5×10^3 and maintained for 24 h at 37 °C and 5% CO_2. The treatment was carried out with 100 µL of different concentrations of compounds **9e** (6.25 to 200 µM) and **9g** (9.38 to 300 µM) prepared by serial dilutions in DMEM medium containing 0.5% DMSO. The plates were incubated for 24 and 48 h.

Cell viability was evaluated by the MTT (3-[4,5-dimethylthiazol-2-yl]-2,5-diphenyltetrazolium bromide, Sigma-Aldrich, USA) dye reduction method, according to Dos Santos *et al.* 2018.[5] After exposition time, the medium was removed and a mix of 15 µL of MTT solution in PBS (5 mg/mL) and 85 µL of DMEM was added in each well. The plate was incubated for 2 h at 37 °C and 5% CO_2 in a humidified atmosphere, subsequently the medium with MTT was removed and 100 µL DMSO was added to dissolve the formazan salts produced by living cells. The absorbance was measured at 595 nm using a SpectraMax® Plus 384 microplate reader (Molecular Devices, USA).

The absorbance values were converted to percentage using the transform tool of the GraphPad Prism version 6.0 (GraphPad Softwares, USA). The cell viability was evaluated by analysis of variance (ANOVA) followed by Bonferroni's multiple comparison test to verify statistical differences between treated groups and DMEM control group. Two-way ANOVA followed by Bonferroni's multiple comparison test was employed to compare means between the exposition time 24 and 48 h. The data were expressed as mean ± standard error of the mean (SEM) of the quintuplicate and differences among means were considered statistically significant at $p<0.05$.

3. Synthetic procedures and analytical data

3.1 Synthetic procedure of Morita–Baylis–Hillman adducts (2)

The adducts were synthesized as previously reported.[6] In a Schlenk flask were added the aldehyde **1** (34.7 mmol), ethyl acrylate (52.5 mmol) and DABCO (5.00 mmol). The mixture was kept protected from the light at room temperature under stirring for 21 days. The reaction mass was concentrated under vacuum. The residue was purified by flash column chromatography on silica gel (20% ethyl acetate in hexane (v/v) as the eluent solution) to furnish the desired product 2.

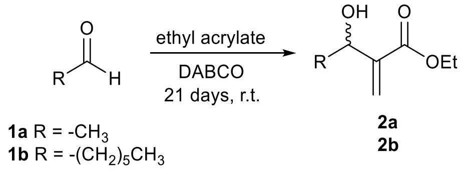

Scheme S1. MBH adducts production reaction.

Ethyl 3-hydroxy-2-methylenebutanoate (2a)[5] Physical state: colourless oil. Yield: 90%.
Ethyl 3-hydroxy-2-methylenenonanoate (2b)[5] Physical state: colourless oil. Yield: 80%.

3.2 Synthetic procedure of hydrolysis of Morita–Baylis–Hillman adducts (3)

The carboxylic acids were synthesized as previously reported.[7] The adduct **2** (23.3 mmol) was dissolved in THF (40 mL), then 60 mL of 3.5 M LiOH solution was added and vigorously stirred at room temperature for 24 h. The THF was removed under vacuo and water (60 mL) were added. The solution was washed with ethyl acetate (3 × 15 mL). The residual aqueous phase was then acidified to pH 1.0 with 20% hydrochloric acid solution and extracted with of ethyl acetate (3 × 20 mL). The organic phases were combined, dried over anhydrous Na_2SO_4, filtered and concentrated in vacuo to furnish the desired product **3**.

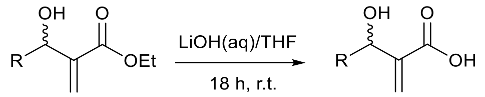

2a R = $-CH_3$ **3a**
2b R = $-(CH_2)_5CH_3$ **3b**

Scheme S2. Hydrolysis of MBH adducts.

3-Hydroxy-2-methylenebutanoic acid (3a)[8] Physical state: yellow oil. Yield: 98%. ^{1}H NMR (600 MH, $CDCl_3$) δ 6.38 (s, 1H), 5.95 (s, 1H), 4.66 (q, *J* = 6.6 Hz, 1H), 1.42 (d, J = 6.6 Hz, 3 H). ^{13}C NMR (75 MHz, $CDCl_3$) δ 171.4, 142.9, 126.8, 67.1, 22.1. HRMS (ESI-TOF): $[M+H]^+$ calculated for $C_5H_9O_3$ 177.0546; found 177.0559.

3-Hydroxy-2-methylenenonanoic acid (3b)[9] Physical state: colourless oil. Yield: 90%. ^{1}H NMR (600 MH, $CDCl_3$) δ 6.39 (s, 1H), 5.92 (s, 1H), 4.42(q, *J* = 6.6 Hz, 1H), 1.73-1.62 (m, 2 H), 1.48-1.24 (m, 8H), 0.88 (t, *J* = 7.0 Hz, 3H). ^{13}C NMR (75 MHz, $CDCl_3$) δ 171.4, 142.0, 127.6, 71.7, 36.3, 31.9, 29.2, 25.9, 22.7, 14.2. HRMS (ESI-TOF): $[M-OH]^+$ calculated for $C_{10}H_{17}O_2$ 169.1223; found 169.1202.

3.3 Procedure of solid-phase peptide synthesis – SPPS

All the cyclopeptides were synthesized manually by Fmoc protection methodology. In a round bottom flask equipped with sintered glass filter used as a container for SPFS (Figure S1), 400 mg of Rink amide AM resin (0.28 mmol) was added. The using repetitive cycles of deprotection with 20% solution of 4-methylpyridine in DMF for 15 min and stirred, and couplings using 4 equiv of the respective amino acid, 4 equiv of HCTU or HATU and 2 equiv of 6-Cl-HOBt as an agent to prevent racemization. The MBH adducts acid **3** were added in the linear sequence **5**, using 4 equiv of **3**, and thus the same equivalence for DIC/Oxyme Pure. The reactions were stirred for 1.5 h. After this time, the reagents in solution were removed by filtration, the resin was sequentially washed with DCM, DMF and further DCM, each wash was done two times.

The hydroxy group at the MBH residue in **6** was acetylated using 10 ml of a mixture of pyridine: acetic anhydride (2:1) for 40 min. This procedure was performed two times with reactive renovation to yield **7** which was cleaved from the resin by the action of 10 mL of the cleavage cocktail (TFA: CH_2Cl_2:TIS; 90:8.5:1.5 (v/v/v)) under stirring for 1 h at rt. The supernatant was filtered from the resin, the resin was washed with 5 mL of cleavage cocktail for 15 min, the supernatant was added to the initial collection and the resin was further washed with 5 ml of TFA (3 × 5 mL) for 15 min. These last washes with TFA were also added to the initial supernatants and concentrated under reduced pressure without heating until obtaining a yellow oil. Subsequently, 30 mL of cold hexane/ethyl ether (1:1) were added, observing the formation of precipitate, which was

centrifuged, and washed with hexane/ethyl ether (3 × 5 mL) to yield the crude mixture of linear peptides containing **8**.

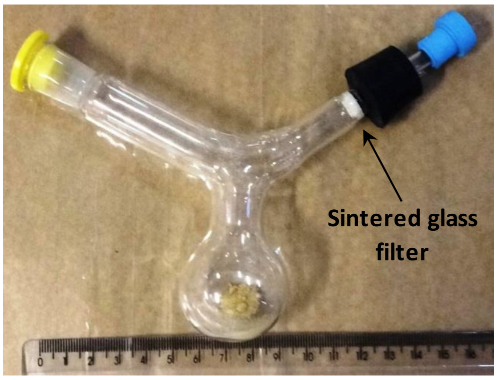

Figure S1. Round bottom flask for SPPF.

3.4 Synthetic procedure for the cyclization reaction.

The cyclization step was performed immediately after the cleavage procedure. For this, the crude mixture of peptides containing **8** obtained from the cleavage and centrifugation was weighed, solubilized in dry THF:CH_2Cl_2 (1:1) to a concentration close to 1mM, and stirred. Then, enough Et_3N was added dropwise so that it reached 3% v/v. The reaction proceeded with vigorous stirring for 48 h at rt. The volatiles were removed under vacuo and the residual product was purified by semi-preparative HPLC as described previously to yield the analogues **9**.

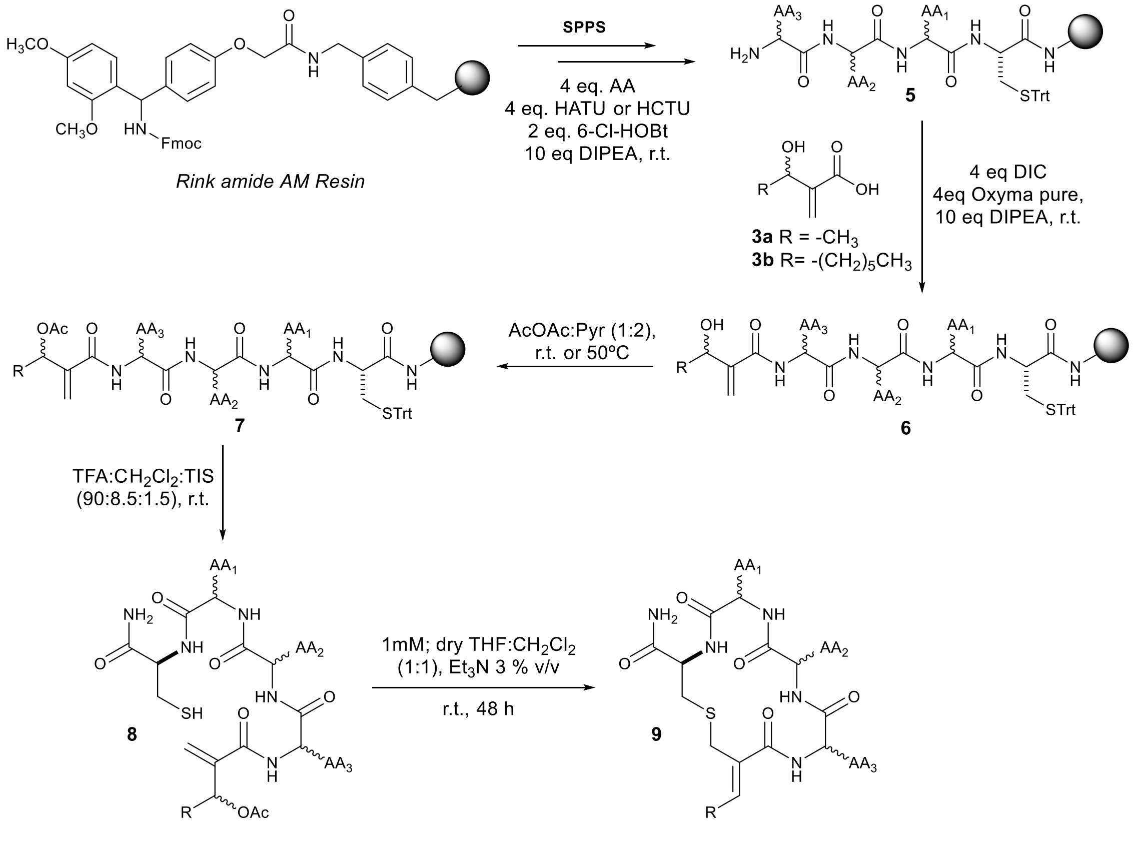

Scheme S3. Synthesis of linear peptides **8** and its cyclization to yield analogues **9**.

3.5 Analytical data of the cyclopeptides

Compounds 9a

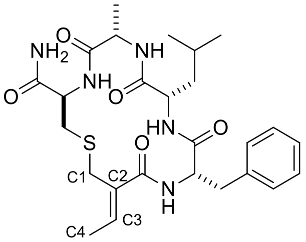

White solid, 10.2% yield; HRMS (ESI-TOF): $[M+H]^+$ calculated for $C_{26}H_{37}N_5O_5S$ 532.2588; found 532.2599 and $[M+Na]^+$ calculated for $C_{26}H_{36}N_5NaO_5S$ 554.2408; found 554.2412; IR-ATR: 3290 (N-H), 1653 (C=O), 1526 (C=C).

Table S2. NMR spectroscopic data (DMSO-d_6) for compound 9a

Amino acid	Position	δH (ppm) (multiplicity, *J* (Hz))	δC (ppm)	COSY/TOCSY	HMBC	NOESY
Cys	NH	7.30 (1H, d, 7.2)	-	Cα, Cβ	Cα, Cβ, CO (Ala)	NH (Ala)
	CO	-	171.79	-	Cα, Cβ	
	Cα	4.34 (1H, td, 9.0, 6.0, 3.6)	55.05 (CH)	NH, Cβ	CO, Cβ	
	Cβ	2.73 (1H, dd, 13.8, 6.00)	31.68 (CH_2)	NH, Cα	Cα, CO, C1	
		2.78 (1H, dd, 13.8, 9.00)				
Ala	NH	7.95 (1H, d, 7.8)	-	Cα, Cβ	Cα, Cβ, CO (Leu)	NH (Cys)
	CO	-	171.49	-	Cα, Cβ	
	Cα	4.09 (1H, m)	49.45 (CH)	NH, Cβ	CO, Cβ	
	Cβ	1.33 (3H, d, 7.2)	16.34 (CH_3)	NH, Cα	Cα, CO	
Leu	NH	8.07 (1H, d, 6.6)	-	Cα	Cα, Cβ, CO (Phe)	NH (Phe)
	CO	-	172.52	-	Cα	
	Cα	4.04 (1H, m)	53.57 (CH)	NH, Cβ	CO, Cβ, Cγ	
	Cβ	1.49 (1H, m)	39.58 (CH_2)	Cα, Cγ, $C\delta_1$, $C\delta_2$	Cγ, Cα, $C\delta_1$, $C\delta_2$	
		1.73 (1H, m)				
	Cγ	1.57 (1H, m)	24.28 (CH)	$C\delta_1$, $C\delta_2$	Cα, Cβ	
	$C\delta_1$	0.84 (3H, d, 6.6)	21.53 (CH_3)	Cβ, Cγ	Cβ, Cγ, $C\delta_2$	
	$C\delta_2$	0.91 (3H, d, 6.6)	22.86 (CH_3)	Cβ, Cγ	Cβ, $C\delta_1$, Cγ	
Phe	NH	8.58 (1H, d, 7.8)	-	Cα, Cβ	Cα, Cβ, CO (Adduct)	C3, NH (Leu)
	CO	-	172.32	-	Cα, Cβ	
	Cα	4.10 (1H, m)	55.90 (CH)	NH, Cβ	CO, Cγ, Cβ, CO (Adduct)	
	Cβ	3.14 (1 H, d, 8.4, 2.4)	35.50 (CH_2)	NH, Cα	Cα, CO, Cm, Cγ	
		3.15 (1H, br. s, 8.4)				
	Cγ	-	138.01	-	Cα, Cβ	
	C*o*	7.29 (2H, m)	128.22 (CH)	n/o	Cγ	
	C*m*	7.21 (2H, m)	129.05 (CH)	n/o	C*p*	
	C*p*	7.22 (1H, m)	126.32 (CH)	n/o	C*m*	
Adduct	CO	-	169.08	-	C1, C3	
	C1	3.29 (1H, d, 13.8)	26.40 (CH_2)	-	CO, C2, C3, Cβ (Cys)	
		3.51 (1H, d, 13.2)		-		
	C2	-	134.19	-	C1, C4	
	C3	6.04 (1H, q, 4.0)	132.02(CH)	C4	C1, C4, CO	NH (Phe)
	C4	1.71 (3H, d, 6.60)	13.63 (CH_3)	C3	C1, C2, C3	

n/o: not observed.

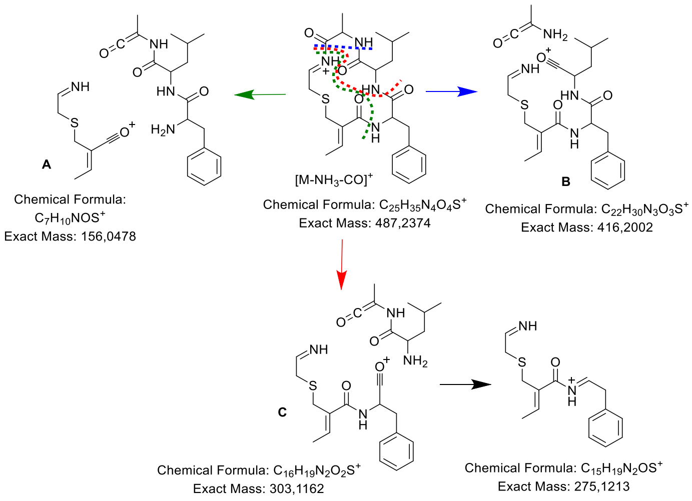

Scheme S4. ESIMS/MS Fragmentations patterns observed for **9a** evidencing the formation of the sulfide group as the macrocyclic linkage.

Compound 9b

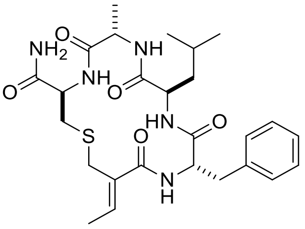

White solid, 14.4% yield; HRMS (ESI-TOF): $[M+H]^+$ calculated for $C_{26}H_{37}N_5O_5S$ 532.2588; found 532.2586 and $[M+Na]^+$ calculated for $C_{26}H_{36}N_5NaO_5S$ 554.2408; found 554.2408; IR-ATR: 3282 (N-H), 1625 (C=O), 1544 (C=C).

Table S3. NMR spectroscopic data (DMSO-d_6) for compound 9b

Amino acid	Position	δH (ppm) (multiplicity, J (Hz))	δC (ppm)	COSY/TOCSY	HMBC	NOESY
Cys	NH	7.78 (d, 1H, 7.8)	-	Cα, Cβ	Cα, Cβ, CO (Ala)	Cα (Ala)
	CO	-	171.71	-	Cα, Cβ	
	Cα	4.18 (1H, m)	52.80 (CH)	NH, Cβ	CO, Cβ	
	Cβ	2.73 (1H, dd, 12.6, 7.8)	32.76 (CH_2)	NH, Cα	Cα, CO, C1	
		2.80 (1H, dd, 12.6, 6.6)				
Ala	NH	8.33 (1H, d, 7.8)	-	Cα, Cβ	Cα, Cβ, CO (Leu)	
	CO	-	171.20	-	Cα, Cβ	
	Cα	4.26 (1H, q, 7.2)	48.28 (CH)	NH, Cβ	CO, Cβ	NH (Cys)
	Cβ	1.17 (3H, d, 7.2)	16.64 (CH_3)	NH, Cα	Cα, CO	
Leu	NH	8.26 (1H, d, 7.2)	-	Cα, Cβ	Cα, Cβ, CO (Phe)	Cα (Phe)
	CO	-	172.29	-	Cα, Cγ	
	Cα	4.16 (1H, m)	51.24 (CH)	NH, Cβ	CO, Cβ, Cγ	
	Cβ	1.40 (1H, m)	39.27 (CH_2)	NH, Cα, Cγ, Cδ$_1$, Cδ$_2$	CO, Cγ, Cα, Cδ$_1$, Cδ$_2$	
		1.43 (1H, m)				
	Cγ	1,33 (1H, m)	23.89 (CH)	Cδ$_1$, Cδ$_2$	Cα, Cβ, Cδ$_1$, Cδ$_2$	
	Cδ$_1$	0.75 (3H, d, 6.6)	21.92 (CH_3)	Cβ, Cγ	Cβ, Cγ, Cδ$_2$	
	Cδ$_2$	0.83 (3H, d, 6.0)	22.68 (CH_3)	Cβ, Cγ	Cβ, Cδ$_1$, Cγ	
Phe	NH	8.14 (1H, d, , 7.8)	-	Cα, Cβ	Cα, Cβ, CO (Adduct)	C3, Ha-C1
	CO	-	171.67	-	Cα, Cβ	
	Cα	4.54 (1H, q, 7.8)	54.47 (CH)	NH, Cβ	CO, Cγ, Cβ, CO	NH (Leu)
	Cβ	2,92 (1 H, dd, 13.2, 9.0)	36.76 (CH_2)	NH, Cα	Cα, CO, Cm/, Cγ	
		2,97 (1H, dd, 13.2, 6.6)				
	Cγ	-	137.98	-	Cα, Cβ, Co, Cm, Cp	
	Co	7.25 (2H, m)	128.03 (CH)	n/o	Cp, Cγ	
	Cm	7.24 (2H, m)	129.16 (CH)	n/o	Cp, Cγ	
	Cp	7.18 (1H, m)	126.19 (CH)	n/o	Co, Cm, Cγ	
Adduct	CO	-	168.00	-	C1, C3	
	C1	3.33 (1H, d, 13.2)	27.33 (CH_2)	-	CO, C2, C3, Cβ (Cys)	NH (Phe)
		3.46 (1H, d, 13.2)		-		
	C2	-	133.60	-	C1, C3, C4	
	C3	6,27 (1H, q, 7.2)	132.24 (CH)	C4	C1, C2, C4, CO	NH (Phe)
	C4	1.73 (3H, d, 6.6)	13.66 (CH_3)	C3	C1, C2, C3	

n/o: not observed; Ha-C1: hydrogen Ha in the methylene carbon C1.

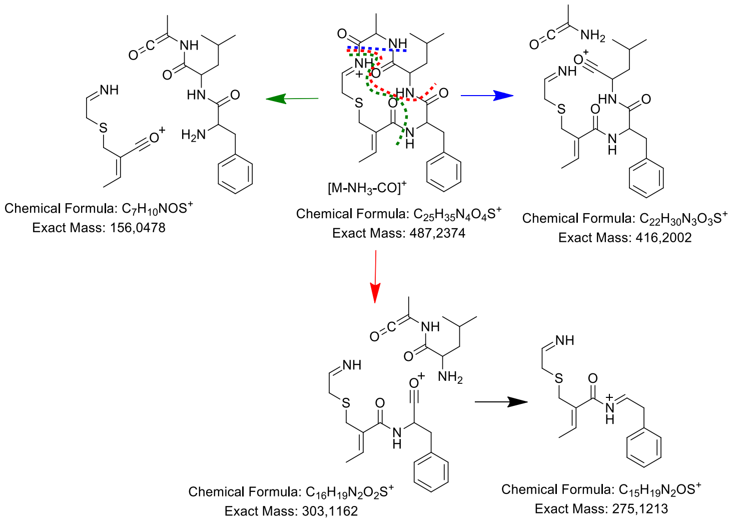

Scheme S5. ESIMS/MS Fragmentations patterns observed for **9b** evidencing the formation of the sulfide group as the macrocyclic linkage.

Compound 9c

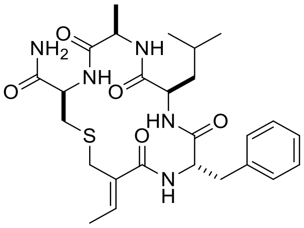

White solid, 7.3% yield; HRMS (ESI-TOF): $[M+H]^+$ calculated for $C_{26}H_{37}N_5O_5S$ 532.2588; found 532.2588 and $[M+Na]^+$ calculated for $C_{26}H_{36}N_5NaO_5S$ 554.2408; found 554.2412; IR-ATR: 3292 (N-H), 1652 (C=O), 1525 (C=C).

Table S4. NMR spectroscopic data (DMSO-d_6) for compound 9c

Amino acid	Position	δH (ppm) (multiplicity, J (Hz))	δC (ppm)	COSY/TOCSY	HMBC	NOESY
Cys	NH	7.60 (1H, d, 7.2)	-	Cα, Cβ	Cα, Cβ, CO (Ala)	
	CO	-	171.75	-	Cα, Cβ	
	Cα	3.99 (1H, m)	54.73 (CH)	NH, Cβ	CO, Cβ	
	Cβ	2.84 (1H, m)	31.07 (CH_2)	NH, Cα	CO, Cα, C1	
		2.87 (1H, m)				
Ala	NH	8.00 (1H, d, 9.0)	-	Cα, Cβ	Cα, Cβ, CO (Leu)	
	CO	-	171.70	-	Cα, Cβ	
	Cα	4.19 (1H, qt, 8.4, 7.2)	49.21 (CH)	NH, Cβ	CO, Cβ	
	Cβ	1.30 (3H, d, 6.6)	17.15 (CH_3)	NH, Cα	Cα, CO	
Leu	NH	8.27 (1H, d, 7.8)	-	Cα, Cβ	Cα, Cβ, CO (Phe)	
	CO	-	171.98	-	Cα	
	Cα	4.02 (1H, m)	51.89 (CH)	NH, Cβ	CO, Cβ, Cγ	
	Cβ	1.33 (1H, m)	39.39 (CH_2)	Cα, $C\delta_1$, $C\delta_2$	Cγ, Cα, $C\delta_1$, $C\delta_2$	
		1.40 (1H, m)				
	Cγ	1.02 (H1, m)	23.61(CH)	$C\delta_1$, $C\delta_2$	Cα, Cβ, $C\delta_1$	
	$C\delta_1$	0.67 (3H, d, 6.6)	21.09 (CH_3)	Cα, Cγ	Cβ, Cγ, $C\delta_2$	
	$C\delta_2$	0.75 (3H, d, 6.6)	23.06 (CH_3)	Cα, Cγ	Cβ, $C\delta_1$	
Phe	NH	8.50 (1H, d, 5.4)	-	Cα, Cβ	Cα, Cβ, CO (Adduct)	C3
	CO	-	171.98	-	Cα, Cβ	
	Cα	4.39 (1H, q, 7.2)	56.09 (CH)	NH, Cβ	CO, Cγ, Cβ	
	Cβ	2.89 (1H, m)	36.63 (CH_2)	NH, Cα	Cα, CO, Cm, Cγ,	
		3.00 (1H, dd, 13.2, 7.2)				
	Cγ	-	137.25	-	C*o*, Cα, Cβ	
	C*o*	7.27 (2H, m)	128.10 (CH)	n/o	Cγ	
	C*m*	7.25 (2H, m)	129.15 (CH)	n/o	C*p*	
	C*p*	7.20 (1H, m)	126.29 (CH)	n/o	Cβ, C*m*	
Adduct	CO	-	169.18	-	C1, C3, C4	
	C1	3.40 (1H, d, 12.60)	27.14 (CH_2)	-	CO, C2, C3, Cβ (Cys)	
		3.49 (1H, d, 12.60)		-		
	C2	-	132.85	-	C1, C4	
	C3	6.28 (1H, q, 7.2, 6.6)	132.64 (CH)	C4	C1, C4, CO	NH (Phe)
	C4	1.74 (3H, d, 6.6)	13.63 (CH_3)	C3	CO, C1, C2	

n/o: not observed; qt: quintet.

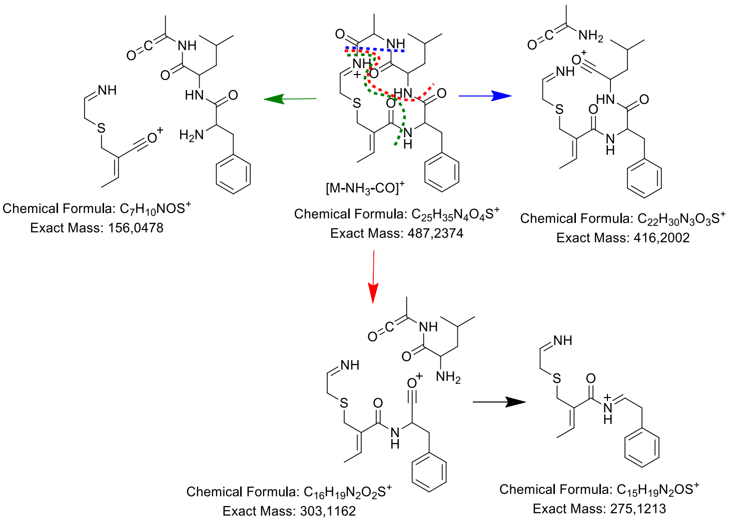

Scheme S6. ESIMS/MS Fragmentations patterns observed for **9c** evidencing the formation of the sulfide group as the macrocyclic linkage.

Compound 9d

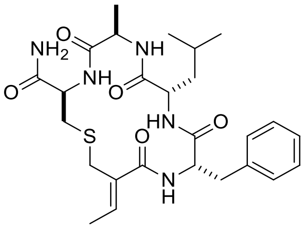

White solid, 2.7% yield; HRMS (ESI-TOF): $[M+H]^+$ calculated for $C_{26}H_{37}N_5O_5S$ 532.2588; found 532.2589 and $[M+Na]^+$ calculated for $C_{26}H_{36}N_5NaO_5S$ 554.2408; found 554.2407; IR-ATR: 3298 (N-H), 1652 (C=O), 1521 (C=C).

Table S5. NMR spectroscopic data (DMSO-d_6) for compound 9d

Amino acid	Position	δH (ppm) (multiplicity, J (Hz))	δC (ppm)	COSY/TOCSY	HMBC	NOESY
Cys	NH	7.21 (1H, d, 8.4)	-	Cα, Cβ	Cα, Cβ, CO (Ala)	
	CO	-	171.75	-	Cα, Cβ	
	Cα	4.20 (1H, m)	52.12 (CH)	NH, Cβ	CO, Cβ	
	Cβ	2.72 (1H, dd, 11.4, 5.4)	33.10 (CH_2)	NH, Cα	CO, Cα, C1	
		2.85 (1H, dd, 12.0, 8.4)				
Ala	NH	8.55 (1H, d, 6.6)	-	Cα, Cβ	Cα, Cβ, CO (Leu)	Cα (Leu)
	CO	-	171.75	-	Cβ	
	Cα	4.03 (1H, t, 6.6)	49.83 (CH)	NH, Cβ	Cβ	
	Cβ	1.23 (3H, d, 7.2)	17.00 (CH_3)	NH, Cα	Cα, CO	
Leu	NH	7.56 (1H, d, 7.2)	-	Cα, Cβ	Cα, CO (Phe)	NH (Phe)
	CO	-	171.22	-	Cα	
	Cα	4.33 (1H, m)	51.60 (CH)	NH, Cβ	CO, Cβ, Cγ	NH (Ala)
	Cβ	1.46 (1H, m)	41.32 (CH_2)	Cα, $C\delta_1$, $C\delta_2$	Cγ, Cα, $C\delta_1$, $C\delta_2$	
		1.51 (1H, m)				
	Cγ	1.46 (H1, m)	24.42 (CH)	$C\delta_1$, $C\delta_2$	Cα, Cβ, $C\delta_1$, $C\delta_2$	
	$C\delta_1$	0.87 (3H, d, 6.6)	22.50 (CH_3)	Cβ, Cγ	Cβ, Cγ, $C\delta_2$	
	$C\delta_2$	0.92 (3H, d, 6.0)	22.73 (CH_3)	Cβ, Cγ	Cβ, $C\delta_1$, Cγ	
Phe	NH	8.25 (1H, d, 8.4)	-	Cα, Cβ	Cα, Cβ, CO (Adduct)	C3, NH (Leu)
	CO	-	171.29	-	Cα, Cβ	
	Cα	4.37 (1H, m)	56.13 (CH)	NH, Cβ	CO, Cγ	
	Cβ	2.93 (1H, dd, 13.8, 11.4)	37.00 (CH_2)	NH, Cα	Cα, CO, C*m*, Cγ,	
		3.04 (1H, dd, 13.8, 4.8)				
	Cγ	-	138.04	-	C*o*, Cα, Cβ	
	C*o*	7.27 (2H, m)	128.19 (CH)	n/o	Cγ	
	C*m*	7.23 (2H, m)	128.92 (CH)	n/o	C*p*	
	C*p*	7.20 (1H, m)	126.36 (CH)	n/o	Cβ, C*m*	
Adduct	CO	-	167.81	-	C1, C3, C4	
	C1	3.38 (1H, bs)	26.98 (CH_2)	-	CO, C2, C3, Cβ (Cys)	
				-		
	C2	-	134.15	-	C1, C4	
	C3	6.06 (1H, q, 7.2, 6.6)	130.65 (CH)	C4	C1, C4, CO	NH (Phe)
	C4	1.71 (3H, d, 6.6)	13.55 (CH_3)	C3	CO, C2, C3	

n/o: not observed; bs: broad singlet

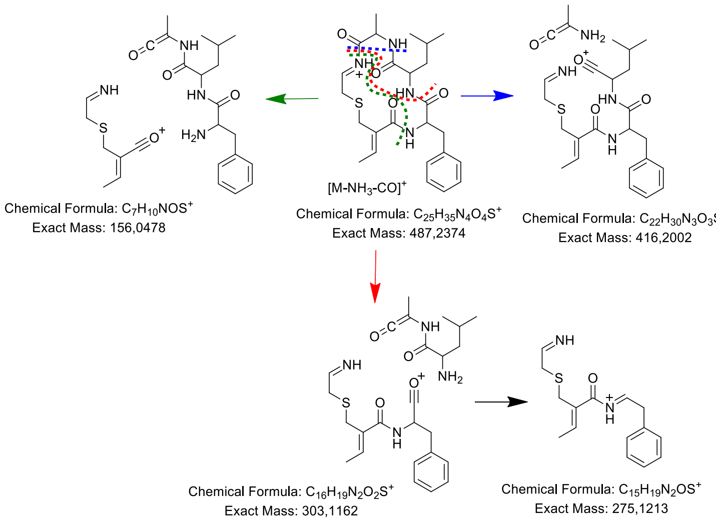

Scheme S7. ESIMS/MS Fragmentations patterns observed for **9d** evidencing the formation of the sulfide group as the macrocyclic linkage.

Compound 9e

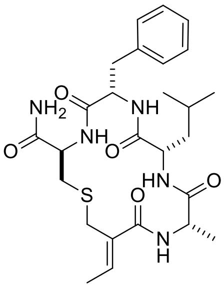

White solid, 14.8% yield; HRMS (ESI-TOF): $[M+H]^+$ calculated for $C_{26}H_{37}N_5O_5S$ 532.2588; found 532.2587 and $[M+Na]^+$ calculated for $C_{26}H_{36}N_5NaO_5S$ 554.2408; found 554.2405; IR-ATR: 3302 (N-H), 1650 (C=O), 1530 (C=C).

Table S6. NMR spectroscopic data (DMSO-d_6) for compound 9e

Amino acid	Position	δH (ppm) (multiplicity, *J* (Hz))	δC (ppm)	COSY/TOCSY	HMBC	NOESY
Cys	NH	7.61 (1H, d, 9.0)	-	Cα, Cβ	Cα, Cβ, CO (Phe)	
	CO	-	171.78	-	Cα, Cβ	
	Cα	4.40 (1H, td, 9.0, 3.6)	55.62 (CH)	NH, Cβ	Cα, CO	
	Cβ	2.76 (1H, dd, 13.8, 3.0)<br>2.88 (1H, dd, 13.8, 9.6)	31.54 (CH_2)	NH, Cα	C1, Cα, CO	
Phe	NH	7.81 (1H, d, 8.4)	-	Cα, Cβ	Cα, Cβ, CO (Leu)	
	CO	-	170.60	-	Cβ	
	Cα	4.34 (1H, ddd, 14.4, 6.0, 4.2)	54.92 (CH)	NH, Cβ	Cβ, Cγ	
	Cβ	2.94 (1H, dd, 13.8, 10.8)<br>3.21 (1H, dd, 13.8, 4.2H)	36.22 (CH_2)	NH, Cα	Cα, C*m*, Cγ, CO	
	Cγ	-	137.95	-	Cα, Cβ, C*o*, C*p*	
	C*o*	7.27 (2H, m)	128.22 (CH)	n/o	Cγ	
	C*m*	7.21 (2H, m)	128.91 (CH)	n/o	C*p*, Cβ	
	C*p*	7.20 (1H, bs)	126.32 (CH)	n/o	Cγ, C*m*	
Leu	NH	7.90 (1H, d, 7.2)	-	Cα, Cβ	Cα, Cβ, CO (Ala)	
	CO	-	172.60	-	Cα, Cβ	
	Cα	4.02 (m, 1H)	53.26 (CH)	NH, Cβ, $C\delta_1$, $C\delta_2$	Cβ, Cγ, CO	
	Cβ	1.21 (1H, m)<br>1.44 (1H, m)	39.63 (CH_2)	Cα, $C\delta_1$, $C\delta_2$	Cα, Cγ, $C\delta_1$, $C\delta_2$, CO	
	Cγ	1.43 (1H, m)	24.22 (CH)	$C\delta_1$, $C\delta_2$	Cα, Cβ, $C\delta_1$, $C\delta_2$	
	$C\delta_1$	0.74 (3H, d, 6.0)	21.61 (CH_3)	Cγ	$C\delta_2$, Cγ, Cβ	
	$C\delta_2$	0.80 (3H, d, 6.0)	22.62 (CH_3)	Cγ	$C\delta_1$, Cγ, Cβ	
Ala	NH	8.64 (1H, d, 6.6)	-	Cα, Cβ	Cα, Cβ, CO, CO (Adduct)	C3
	CO	-	173.16	-	Cα, Cβ	
	Cα	3.92 (1H, qt, 7.2)	50.65 (CH)	NH, Cβ	Cβ, CO, CO (Adduct)	
	Cβ	1.37 (3H, d, 7.2)	16.80 (CH_3)	NH, Cα	Cα, CO	
Adduct	CO	-	169.35	-	C1, C3	
	C1	3.34 (1H, d, 13,2)<br>3.53 (1H, d, 13,8)	26.78 (CH_2)	-<br>-	Cβ (Cys), CO, C2, C3	C4
	C2	-	134.31	-	C1, C4	
	C3	6.39 (1H, q, 7.2, 6.6)	132.78 (CH)	C4	C4, C1, CO	NH (Ala)
	C4	1.78 (3H, d, 7.2)	13.70 (CH_3)	C3	CO, C1, C2, C3	

n/o: not observed; qt: quintet; bs: broad singlet.

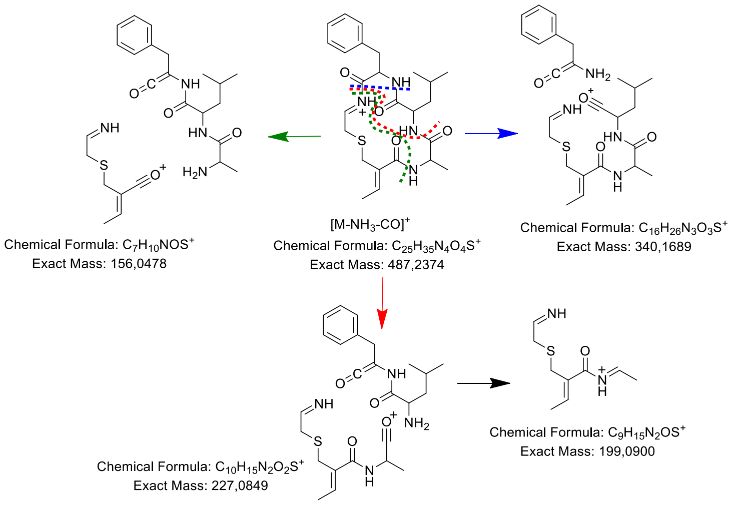

Scheme S8. ESIMS/MS Fragmentations patterns observed for **9e** evidencing the formation of the sulfide group as the macrocyclic linkage.

Compound 9f

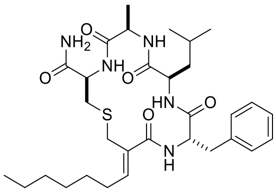

White solid, 11.8% yield; HRMS (ESI-TOF): $[M+H]^+$ calculated for $C_{31}H_{47}N_5O_5S$ 602.3371; found 602.3358 and $[M+Na]^+$ calculated for $C_{31}H_{46}N_5NaO_5S$ 624.3190; found 624.3174; IR-ATR: 3282 (N-H), 1652 (C=O), 1522 (C=C).

Table S7. NMR spectroscopic data (DMSO-d_6) for compound 9f

Amino acid	Position	δH (ppm) (multiplicity, *J* (Hz))	δC (ppm)	COSY/TOCSY	HMBC	NOESY
Cys	NH	7.60 (1H, d, 7.2)	-	Cα, Cβ	Cα, Cβ, CO (Ala)	Hb-C1
	CO	-	171.70	-	Cα, Cβ	
	Cα	3.99 (1H, m)	54.67 (CH)	NH, Cβ	CO, Cβ	
	Cβ	2.85 (1H, m)<br>2.87 (1H, m)	31.27 (CH_2)	NH, Cα	Cα, CO, C1	
Ala	NH	8.00 (1H, d, 8.4)	-	Cα, Cβ	Cα, Cβ, CO (Leu)	Cα (Leu),
	CO	-	171.77	-	Cα, Cβ	
	Cα	4.20 (1H, qt, 7.2)	49.19 (CH)	NH, Cβ	CO, Cβ	
	Cβ	1.30 (3H, d, 7.2)	17.17 (CH_3)	NH, Cα	Cα, CO	
Leu	NH	8.25 (1H, d, 7.8)		Cα, Cβ, Cγ, $Cδ_1$, $Cδ_2$	Cα, Cβ, CO (Phe)	Cα (Phe), Cγ
	CO	-	171.99	-	Cα, Cβ	
	Cα	4.02 (1H, m)	51.88 (CH)	NH, Cβ	CO, Cβ, Cγ	NH (Ala)
	Cβ	1.33 (1H, m)<br>1.40 (1H, m)	39.51 (CH_2)	NH, Cα, Cγ, $Cδ_1$, $Cδ_2$	CO, Cα, $Cδ_1$	
	Cγ	1.00 (1H, m)	23.58 (CH)	$Cδ_1$, $Cδ_2$	Cα, $Cδ_1$, $Cδ_2$	NH
	$Cδ_1$	0.67 (3H, d, 6.6)	21.10 (CH_3)	Cγ	Cβ, Cγ	
	$Cδ_2$	0.75 (3H, d, 6.6)	23.07 (CH_3)	Cγ	Cβ, $Cδ_1$, Cγ	
Phe	NH	8.50 (1H, d, 6.00)		Cα, Cβ	Cα, Cβ, CO (Adduct)	Ha-C1, C3
	CO	-	171.96	-	Cα, Cβ	
	Cα	4.38 (1H, q, 7.2)	56.13 (CH)	NH, Cβ	CO, Cβ	NH (Leu)
	Cβ	2.91 (1H, m)<br>3.00 (1H, dd, 13.2, 7.2)	36.63 (CH_2)	NH, Cα	CO, Cα, Cγ, C*m*	
	Cγ	-	137.26	-	C*o*, Cβ,	
	C*o*	7.26 (2H, m)	128.08 (CH)	n/o	Cβ, Cγ	
	C*m*	7.25 (2H, m)	129.19 (CH)	n/o	C*p*	
	C*p*	7.20 (1H, m)	126.30 (CH)	n/o	C*m*	
Adduct	CO	-	169.15	-	C1, C3	
	C1	3.38 (1H, d, 12.0)<br>3.48 (1H, d, 12.6)	27.42 (CH_2)	-<br>-	CO, C2, C3, Cβ (Cys)	NH (Phe)<br>NH (Cys)
	C2	-	131.75	-	C1, C4	
	C3	6.21 (1H, t, 7.8)	137.96 (CH)	C4, C6	C1, C4, C6, CO	NH (Phe)
	C4	2.12 (2H, q, 7.2)	27.67 (CH_2)	C3, C5, C6	C2, C3, C5	
	C5	1.29 (2H, m)	28.40 (CH_2)	C4	C6, C7	
	C6	1.37 (2H, m)	28.40 (CH_2)	C3, C4	C5, C7	
	C7	1.26 (2H, m)	31.11 (CH_2)	C9	C8	
	C8	1.28 (2H, m)	22.04 (CH_2)	C9	C9	
	C9	0.87 (3H, t, 6.6)	13.96 (CH_3)	C7, C8	C7, C8	

n/o: not observed; Ha-C1: hydrogen Ha in the methylene carbon C1; Hb-C1: hydrogen Hb in the methylene carbon C1; qt: quintet.

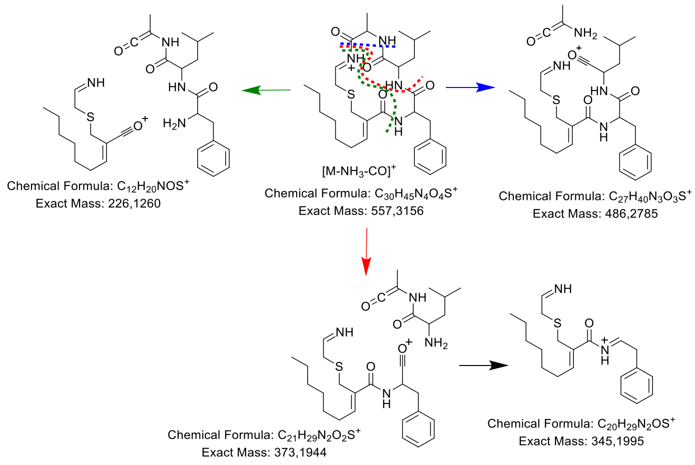

Scheme S9. ESIMS/MS Fragmentations patterns observed for **9f** evidencing the formation of the sulfide group as the macrocyclic linkage.

Compound 9g

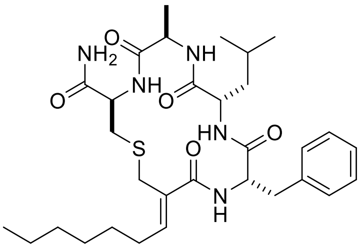

White solid, 4.7% yield; HRMS (ESI-TOF): $[M+H]^+$ calculated for $C_{31}H_{47}N_5O_5S$ 602.3371; found 602.3378 and $[M+Na]^+$ calculated for $C_{31}H_{46}N_5NaO_5S$ 624.3190; found 624.3199; IR-ATR: 3300 (N-H), 1630 (C=O), 1533 (C=C).

Table S8. NMR spectroscopic data (DMSO-d_6) for compound 9g

Amino acid	Position	δH (ppm) (multiplicity, *J* (Hz))	δC (ppm)	COSY/TOCSY	HMBC	NOESY
Cys	NH	7.21 (1H, d, 8.4)	-	Cα, Cβ	Cα, Cβ, CO (Ala)	
	CO	-	171.77	-	Cα, Cβ	
	Cα	4.19 (1H, dt, 8.4, 4.8)	52.12 (CH)	NH, Cβ	CO, Cβ	
	Cβ	2.71 (1H, dd, 11.4, 5.4)	33.25 (CH_2)	Cα, NH	CO, Cα	
		2.83 (1H, dd, 12.0, 8.4)				
Ala	NH	8.56 (1H, d, 6.6)	-	Cα, Cβ	Cα, Cβ, CO (Leu)	Cα (Ala)
	CO	-	171.81	-	Cα, Cβ	
	Cα	4.03 (1H, qt; 7.2)	49.88 (CH)	NH, Cβ	CO, Cβ	
	Cβ	1.23 (3H, d, 7.2)	17.00 (CH_3)	NH, Cα	Cα, CO	
Leu	NH	7.57 (1H, d, 7.2)	-	Cα, Cβ	Cα, CO (Phe)	NH (Phe)
	CO	-	171,26	-	Cβ	
	Cα	4.33 (1H, m)	51.67 (CH)	NH, Cβ	CO, Cβ, Cγ	NH (Ala)
	Cβ	1.46 (1H, m)	41.33 (CH_2)	NH, Cα, $C\delta_1$, $C\delta_2$	Cα, $C\delta_1$, $C\delta_2$	
		1.53 (1H, m)				
	Cγ	1.47 (1H, m)	24.46 (CH)	$C\delta_1$, $C\delta_2$	Cα, CO, $C\delta_1$, $C\delta_2$	
	$C\delta_1$	0.88 (3H, m)	22.77(CH_3)	Cγ, Cβ	Cβ, Cγ	
	$C\delta_2$	0.92 (3H, d, 6.0)	22.51 (CH_3)	Cγ, Cβ	Cβ, $C\delta_1$, Cγ	
Phe	NH	8.20 (1H, d, 8.4)	-	Cα, Cβ	Cα, Cβ, CO (Adduct)	C3, NH (Leu)
	CO	-	171.32	-	Cα	
	Cα	4.35 (1H, m)	56.15 (CH)	NH, Cβ	Cβ, Cγ, CO (Adduct)	
	Cβ	2.93 (1H, dd, 13.2, 10.8)	36.98 (CH_2)	NH, Cα	Cα, Cγ	
		3.05 (1H, dd, m, 13.8, 4.8)				
	Cγ	-	138.06	-	Cα, Cβ, C*o*	
	C*o*	7.26 (m)	128.16 (CH)	n/o	C*m*, Cγ	
	C*m*	7.23 (m)	129.01 (CH)	n/o	C*p*, Cβ	
	C*p*	7.19 (m)	126.38 (CH)	n/o	C*m*	
Adduct	CO	-	167.97	-	C1, C3	
	C1	3.36 (2H, bs)	27.34 (CH_2)	-	CO, C2, C3, Cβ (Cys)	C4
				-		
	C2	-	133.17 (CH)		C1, C4	
	C3	5.91 (1H, t, 7.8)	135.99 (CH)	C4, C6	C1, C4, C6, CO	NH (Phe)
	C4	2.08 (2H, m)	27.34 (CH_2)	C3, C6	C2, C3, C5	C1
	C5	1.25 (2H, m)	28.29 (CH_2)	C6	C1, C8	
	C6	1.32 (2H, m)	28.51 (CH_2)	C4, C5	C5, C7	
	C7	1.25 (2H, m)	31.10 (CH_2)	C9	C6, C9	
	C8	1.27 (2H, m)	22.07(CH_2)	C9	C5, C7, C9	
	C9	0.87 (3H, t)	13.98 (CH_3)	C6, C7, C8	C7, C8	

n/o: not observed; dt: doublet of triplets; qt: quintet.

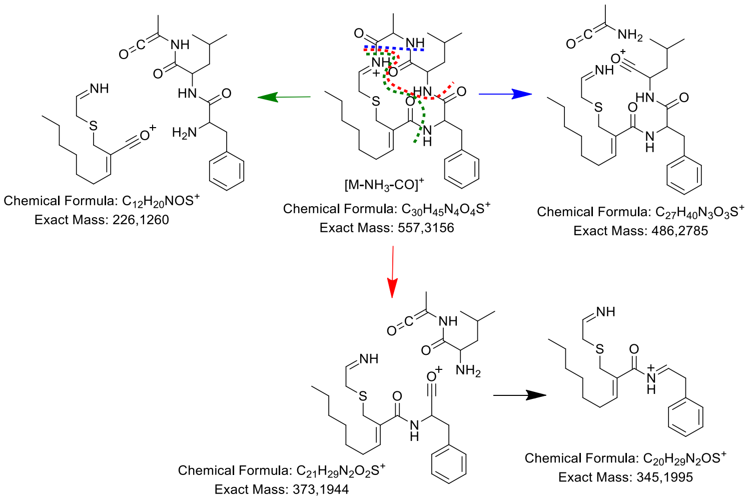

Scheme S10. ESIMS/MS Fragmentations patterns observed for **9g** evidencing the formation of the sulfide group as the macrocyclic linkage.

Compound 9h

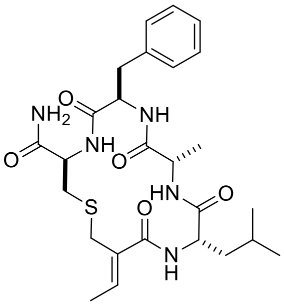

White solid, 7.6% yield; HRMS (ESI-TOF): $[M+H]^+$ calculated for $C_{26}H_{37}N_5O_5S$ 532.2588; found 532.2587 and $[M+Na]^+$ calculated for $C_{26}H_{36}N_5NaO_5S$ 554.2408; found 554.2413; IR-ATR: 3285 (N-H), 1648 (C=O), 1521 (C=C).

Table S9. NMR spectroscopic data (DMSO-d_6) for compound 9h

Amino acid	Position	δH (ppm) (multiplicity, J (Hz))	δC (ppm)	COSY/TOCSY	HMBC	NOESY
Cys	NH	7.74 (1H, d, 7.2)	-	Cα, Cβ	Cα, Cβ, CO (Phe)	
	CO	-	171.63	-	Cα, Cβ	
	Cα	4.00 (1H, ddd, 9.0, 7.2, 4.2)	54.48 (CH)	NH, Cβ	Cβ, CO, CO (Phe)	
	Cβ	2.84 (1H, dd, 13.2, 9.0)<br>2.88 (1H, dd, 13.2, 4.8)	31.06 (CH_2)	NH, Cα	C1, Cα, CO	
Phe	NH	8.00 (1H, d, 9.0)	-	Cα, Cβ	Cα, CO (Ala)	
	CO	-	170.74	-	Cα, Cβ	
	Cα	4.28 (1H, m)	55.33 (CH)	NH, Cβ	Cβ, Cγ, CO	NH (Ala)
	Cβ	2.99 (1H, dd, 13.8, 11.4)<br>3.15 (1H, dd, 13.8, 4.2)	36.30 (CH_2)	NH, Cα	Cα, Cm, Cγ, CO	
	Cγ	-	138.02	-	C*o*	
	C*o*	7.27 (2H, m)	128.14 (CH)	n/o	Cγ	
	C*m*	7.21 (2H, m)	129.00 (CH)	n/o	C*p*, Cβ	
	C*p*	7.20 (1H, bs)	126.24 (CH)	n/o	Cm	
Ala	NH	8.44 (1H, d, 6.6)	-	Cα, Cβ	Cα, Cβ, CO (Leu)	Cα (Phe)
	CO	-	172.43	-	Cα, Cβ,	
	Cα	3.94 (1H, q, 6.6)	49.85 (CH)	NH, Cβ	Cβ, CO, CO (Leu)	
	Cβ	1.03 (3H, d, 7.2)	16.83 (CH_3)	NH, Cα	Cα, CO	
Leu	NH	8.17 (1H, d, 6.0)	-	Cα, Cβ	Cα, CO (Adduct)	C3
	CO	-	172.98	-	Cα, Cβ	
	Cα	4.30 (1H, m)	52.10 (CH)	NH, Cβ, $C\delta_1$, $C\delta_2$	Cβ, Cγ, CO, CO (Adduct)	
	Cβ	1.41 (1H, m)<br>1.58 (1H, m)	39.96 (CH_2)	Cβ, Cα, $C\delta_1$, $C\delta_2$	Cα, Cγ, $C\delta_1$, $C\delta_2$, CO	
	Cγ	1.63 (1H, m)	24.30 (CH)	$C\delta_1$, $C\delta_2$	Cα, Cβ, $C\delta_1$, $C\delta_2$	
	$C\delta_1$	0.88 (3H, d, 6.6)	21.75 (CH_3)	Cγ	$C\delta_2$, Cγ, Cβ	
	$C\delta_2$	0.90 (3H, d, 6.6)	23.03 (CH_3)	Cγ	$C\delta_1$, Cγ, Cβ	
Adduct	CO	-	168.72	-	C1, C3	
	C1	3.44 (1H, d, 13.2)<br>3.55 (1H, d, 13.2)	26.96 (CH_2)	-	Cβ (Cys), CO, C2, C3	
	C2	-	132.85	-	C1, C4	
	C3	6.37 (1H, q, 7.2)	132.55 (CH)	C4	C4, C1, CO	C4, NH (Leu)
	C4	1.73 (3H, d, 6.6)	13.55 (CH_3)	C3	CO, C1, C2, C3	C3

n/o: not observed; bs: broad singlet

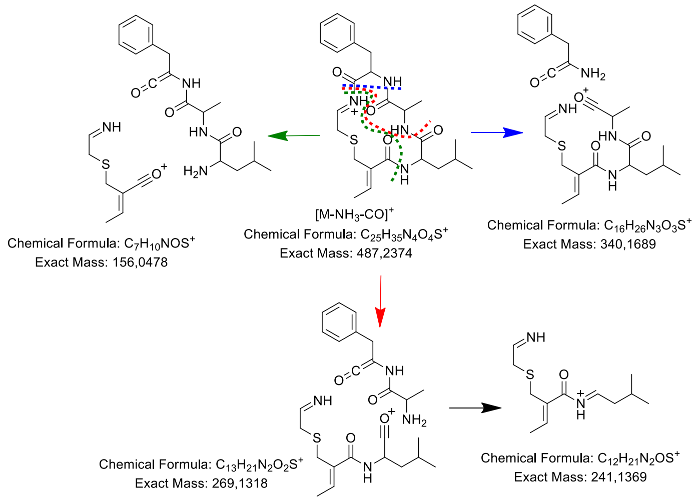

Scheme S11. ESIMS/MS Fragmentations patterns observed for **9h** evidencing the formation of the sulfide group as the macrocyclic linkage.

4. NMR and MS data

Characterization data for MBH adducts carboxylic acids (3)

^1H NMR Spectra of **3a** in $CDCl_3$

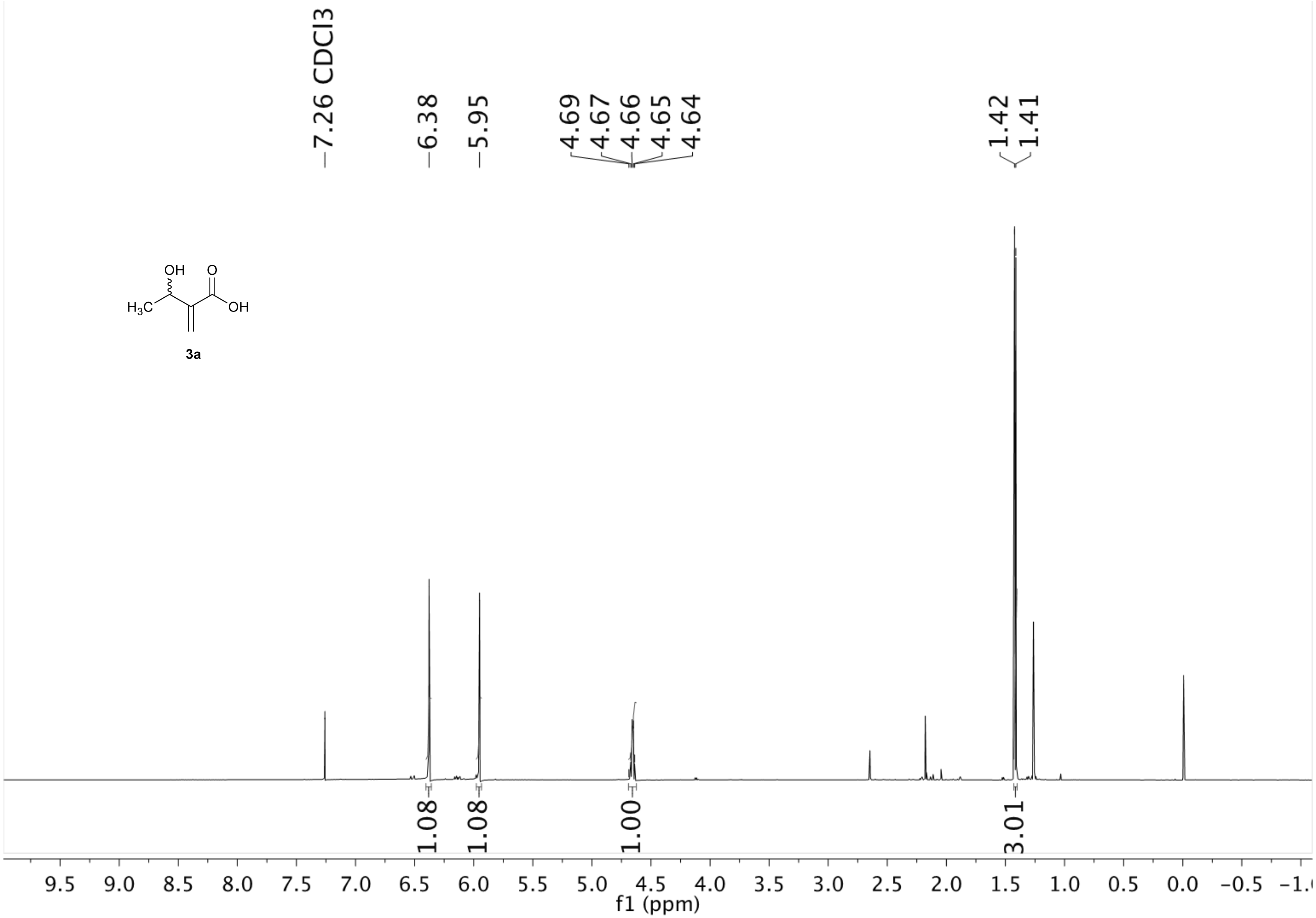

^{13}C NMR Spectra of **3a** in $CDCl_3$

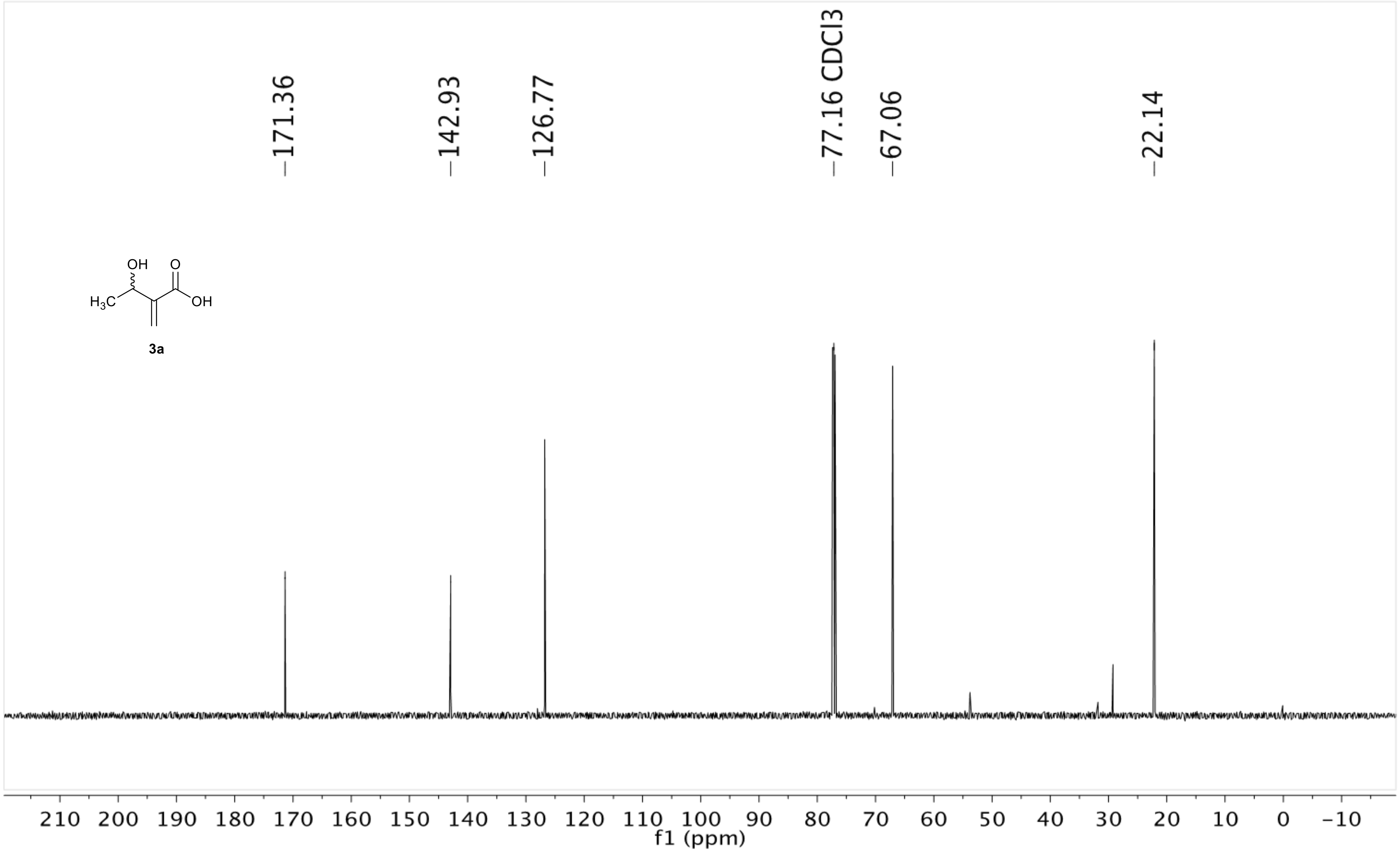

ESI-HRMS Spectra of **3a**

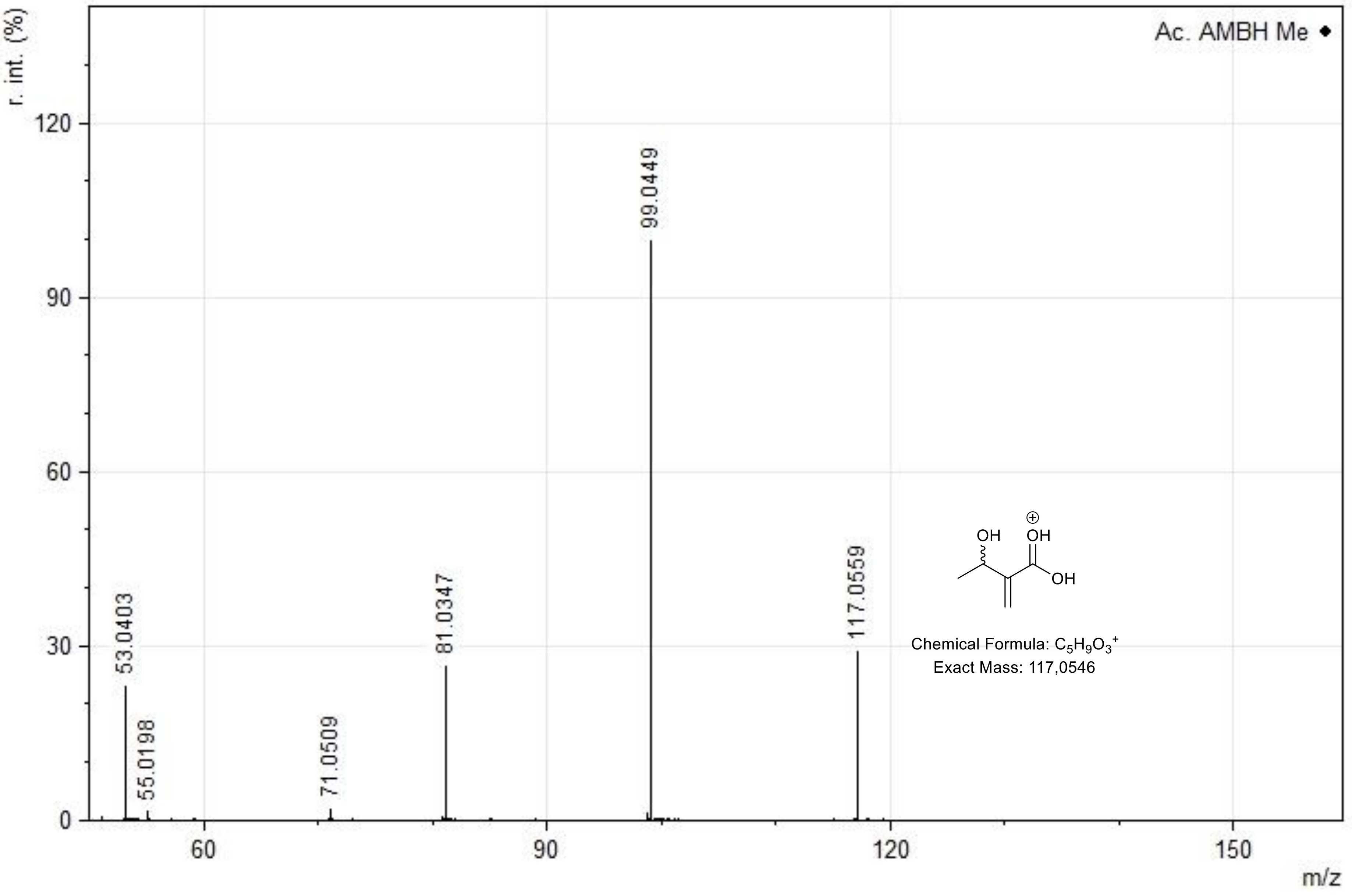

^{1}H NMR Spectra of **3b** in $CDCl_3$

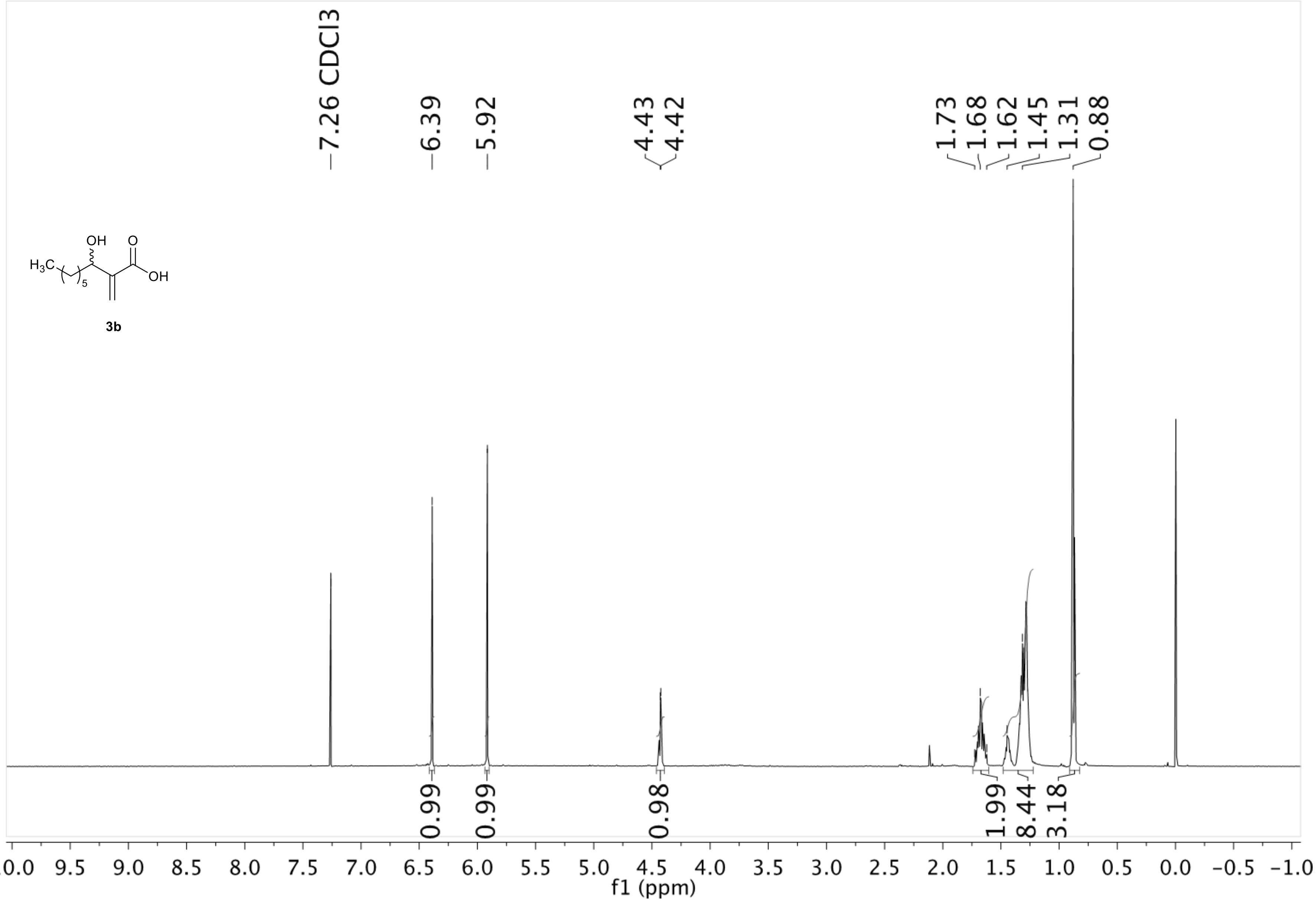

^{13}C NMR Spectra of **3b** in $CDCl_3$

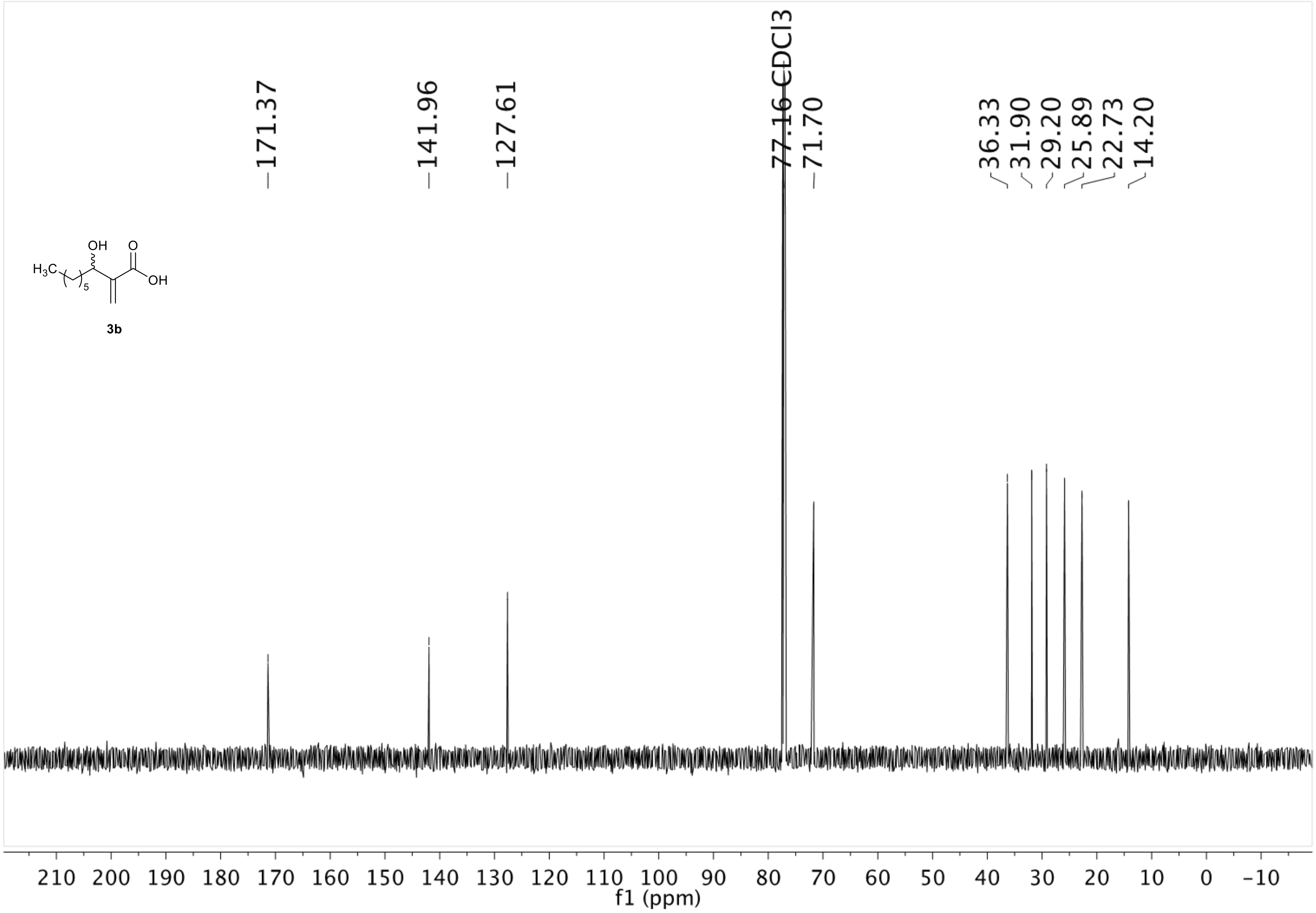

ESI-HRMS Spectra of **3b**

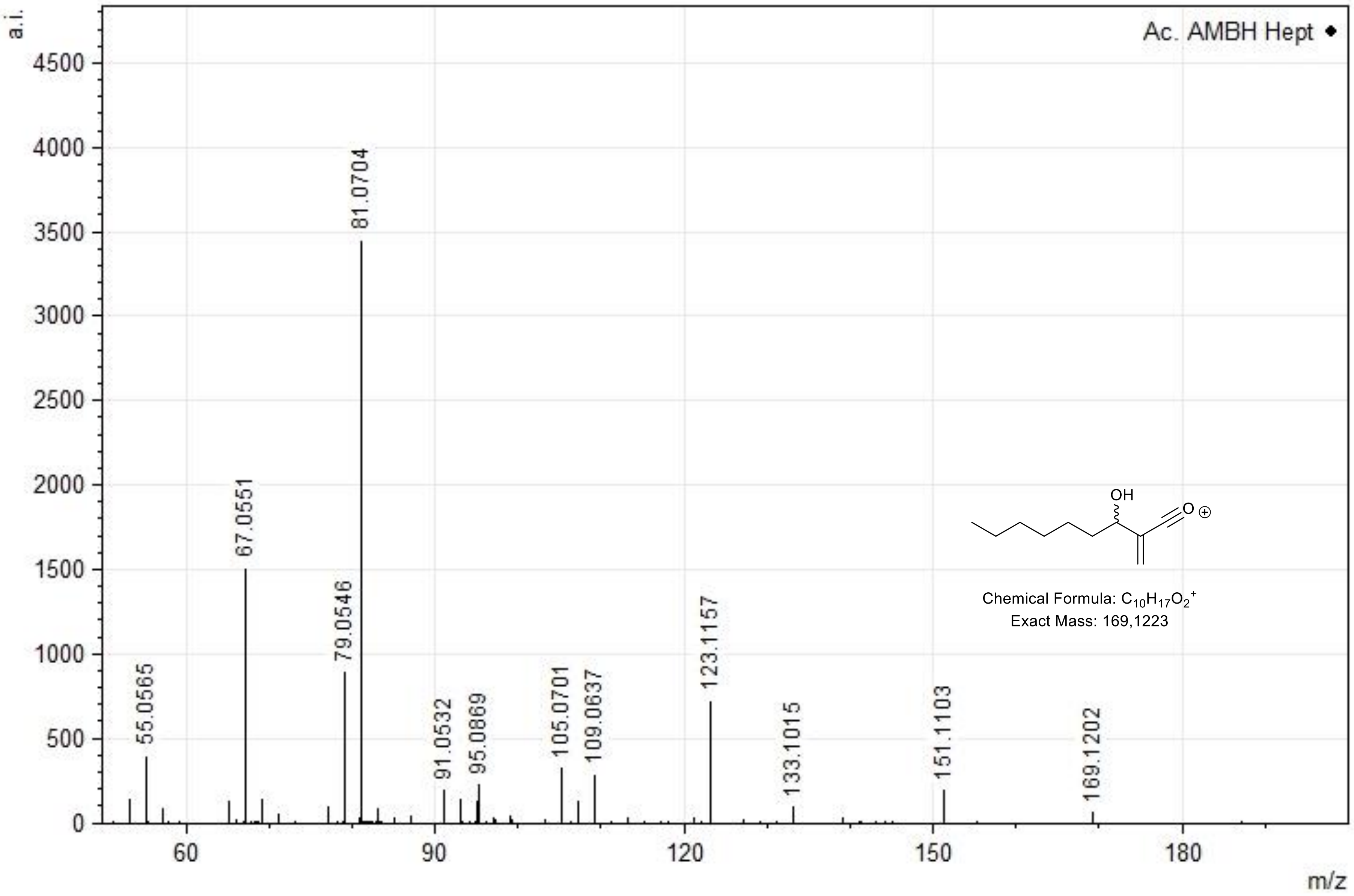

Characterization data for Compound 9a

^{1}H NMR Spectra of **9a** in DMSO-d_6

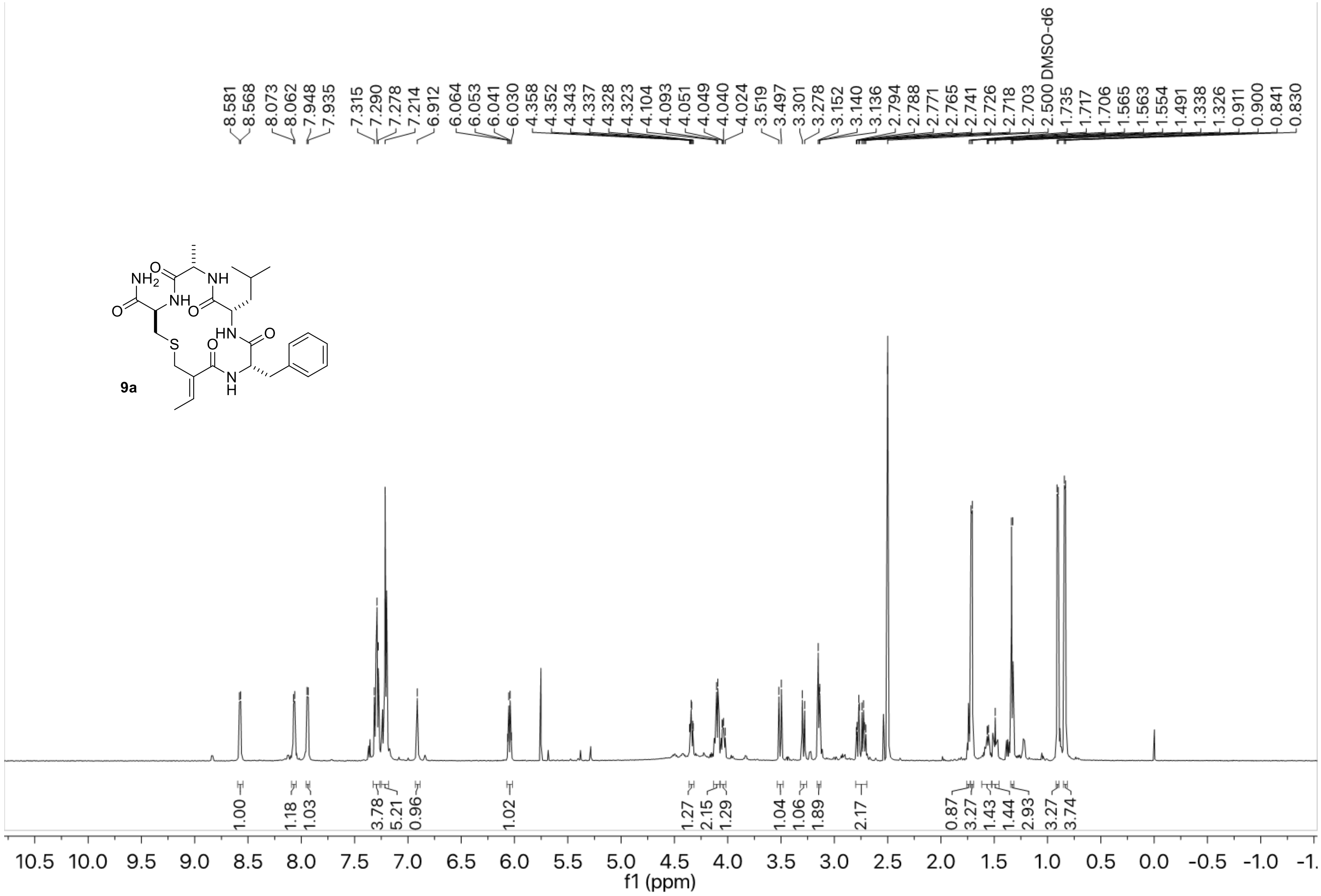

^{13}C NMR Spectra of **9a** in DMSO-d_6

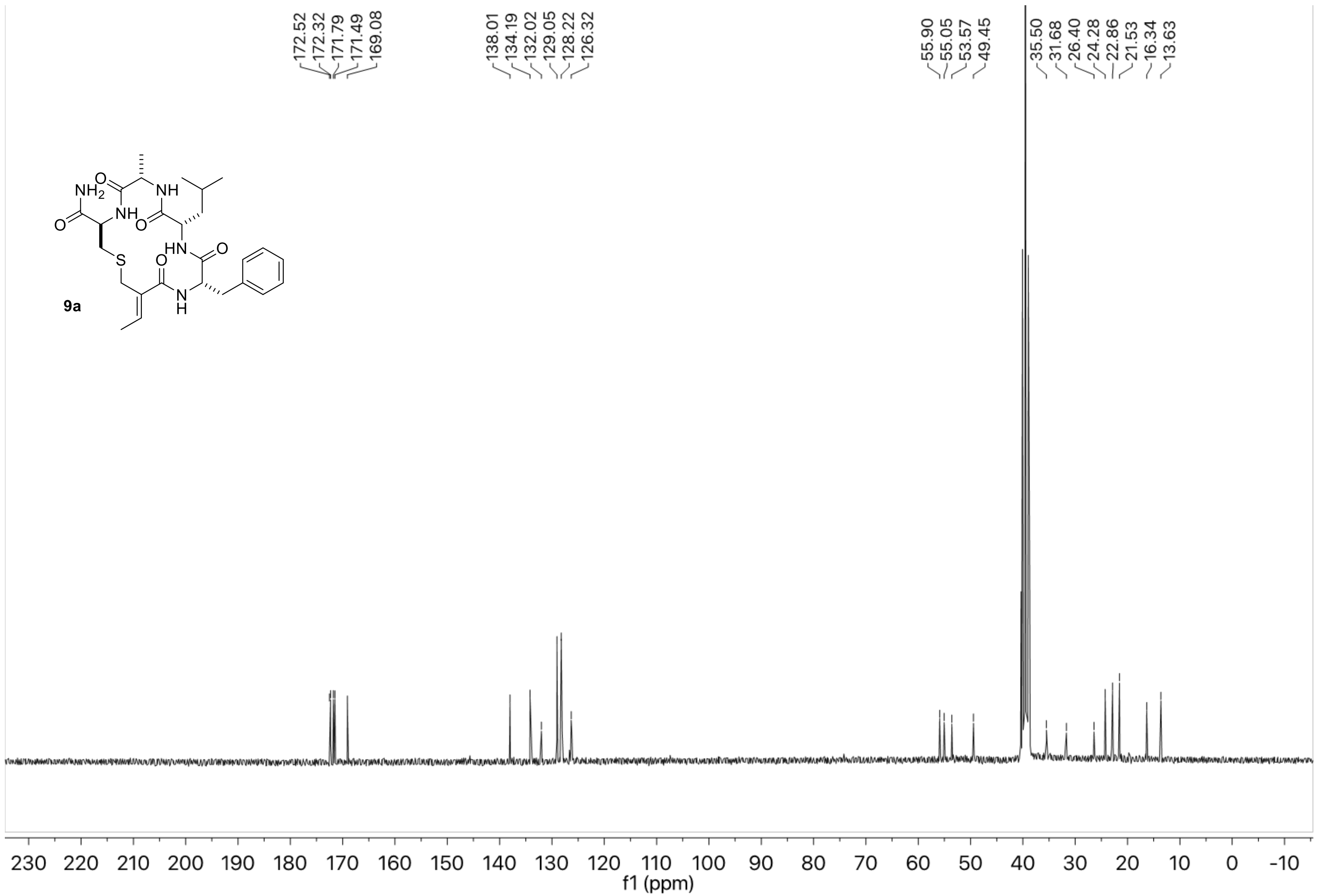

HSQC Spectra of **9a** in DMSO-d_6

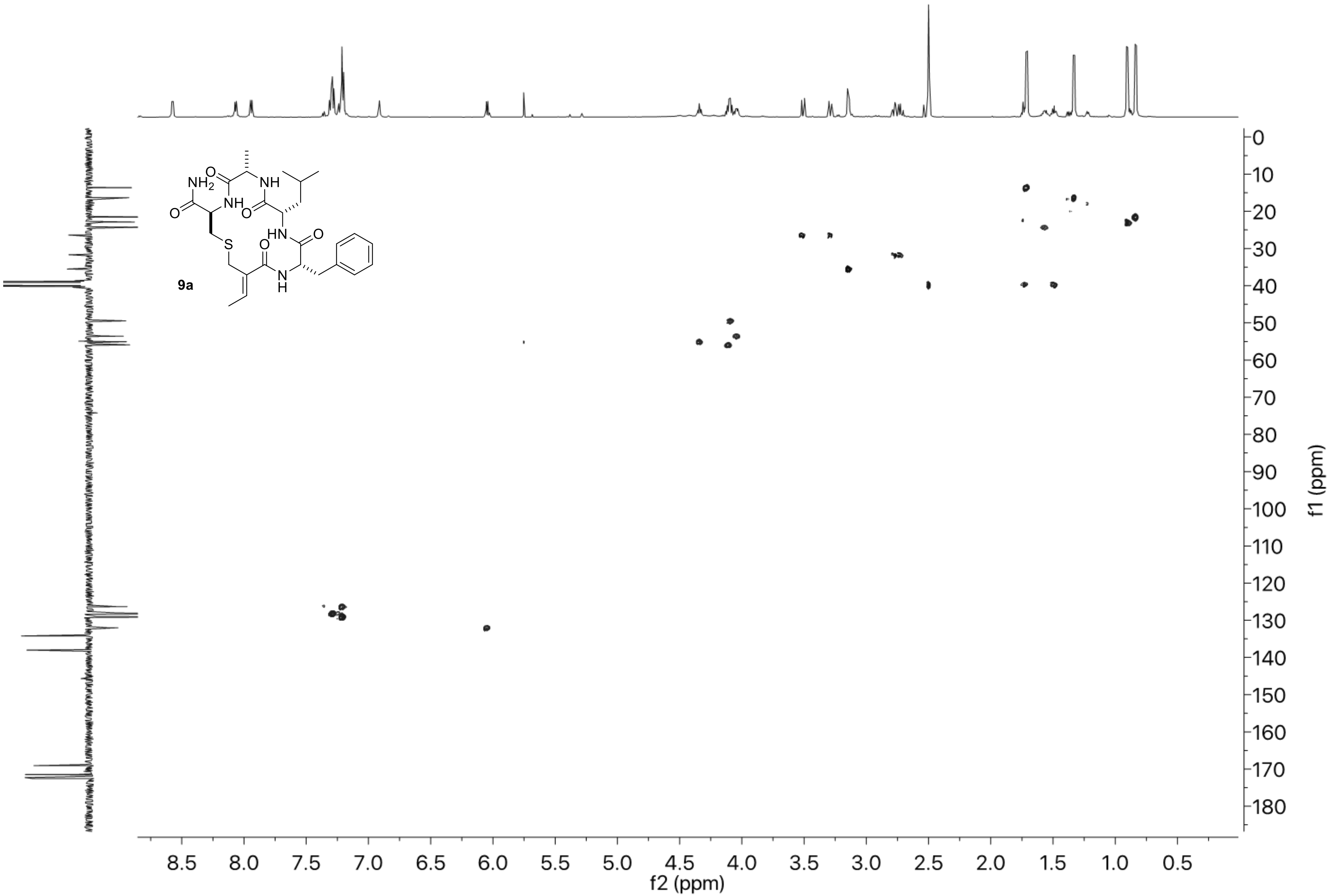

HMBC Spectra of **9a** in DMSO-d_6

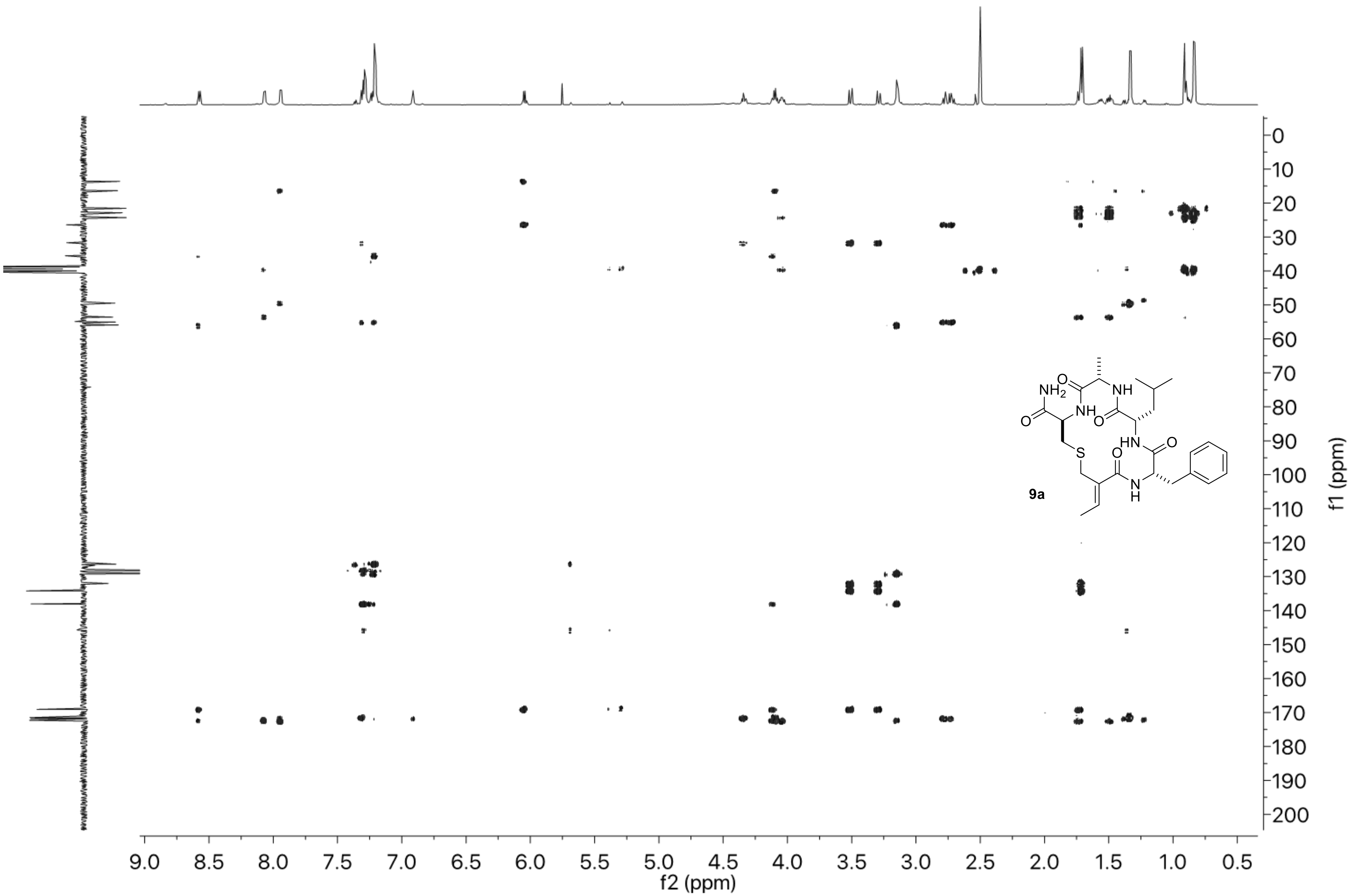

HRMS-ESI-TOF, HRMS/MS spectra, chromatogram and IR-ATR spectrum of **9a**

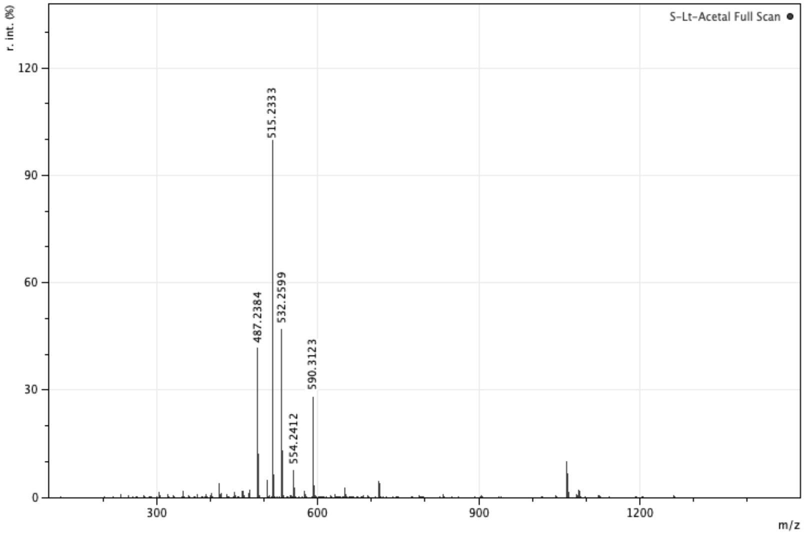

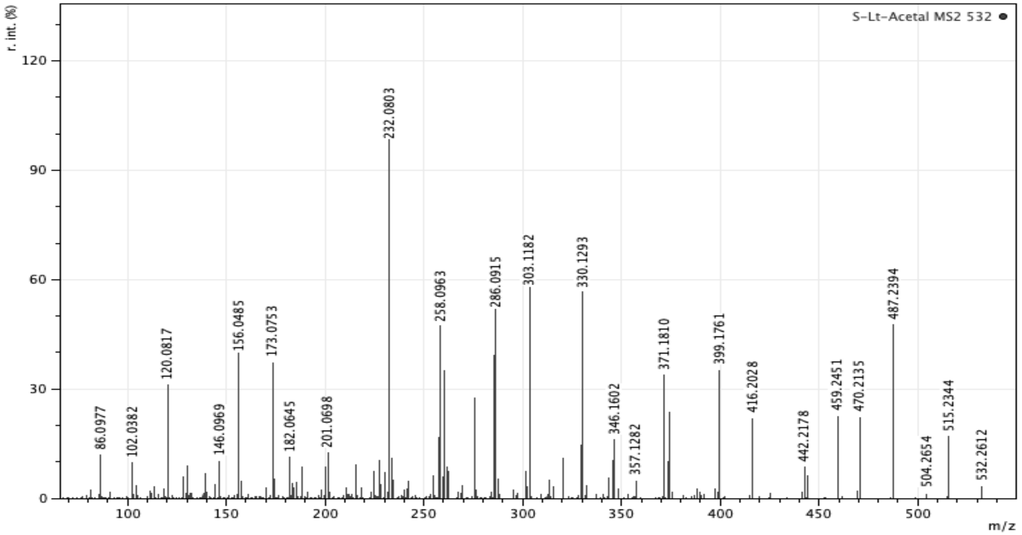

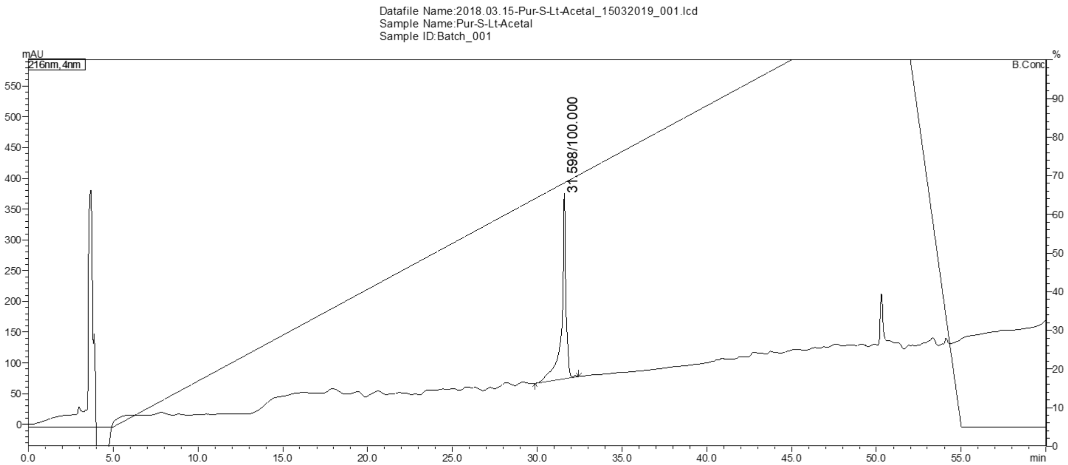

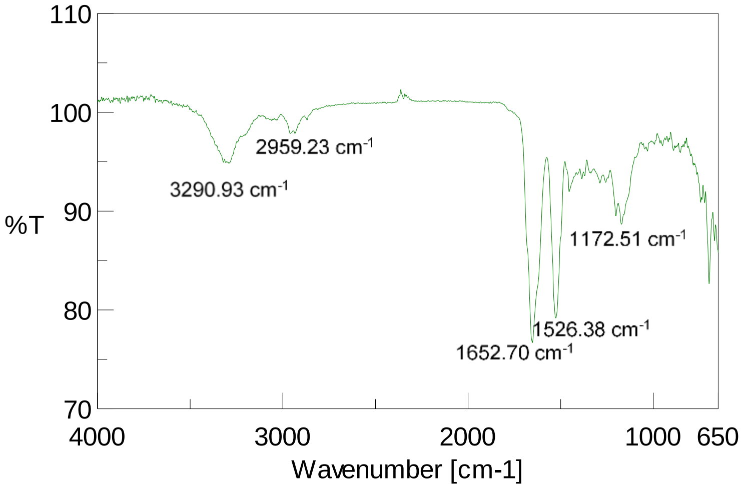

Characterization data for Compound 9b

^{1}H NMR Spectra of **9b** in DMSO-d_6

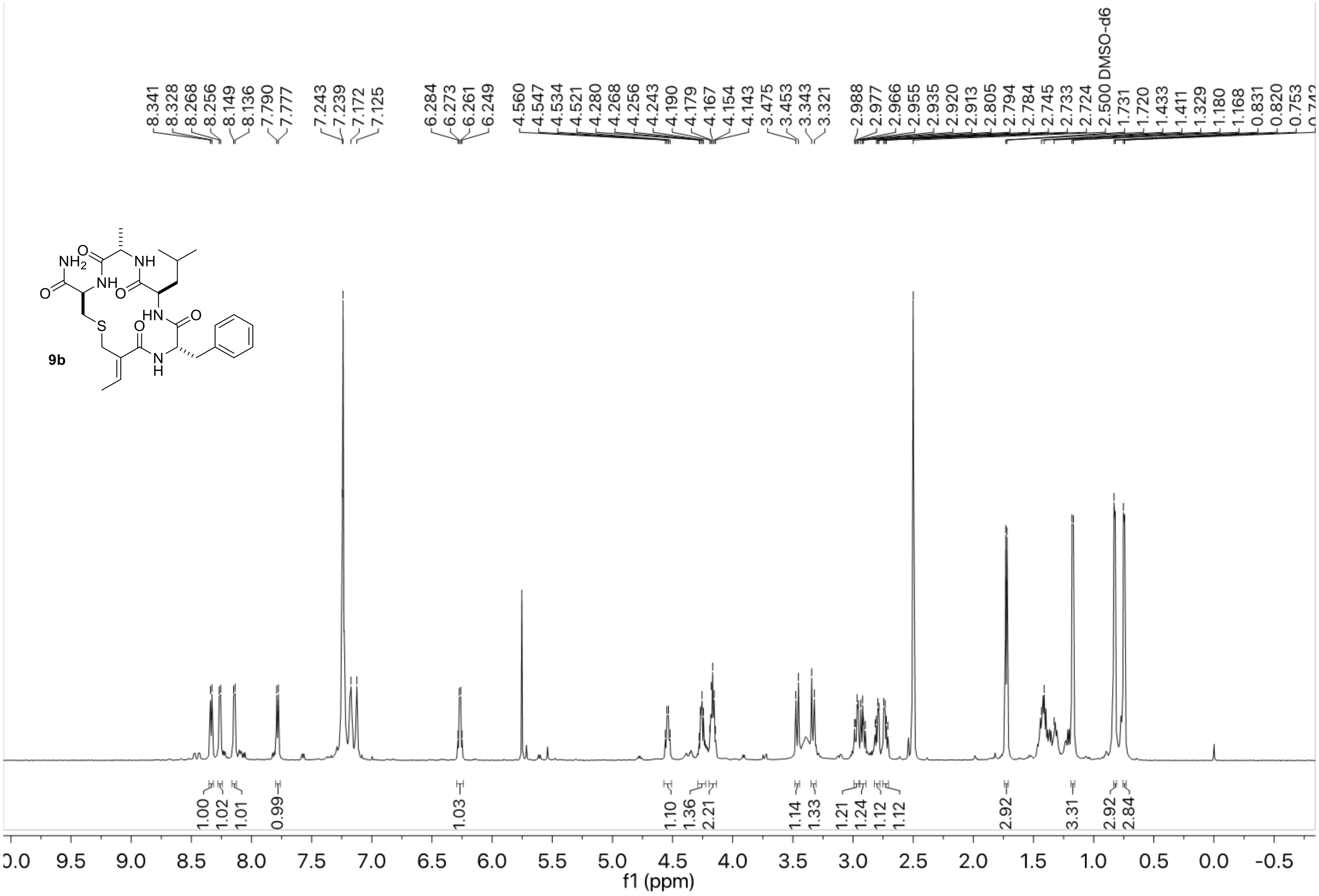

^{13}C NMR Spectra of **9b** in DMSO-d_6

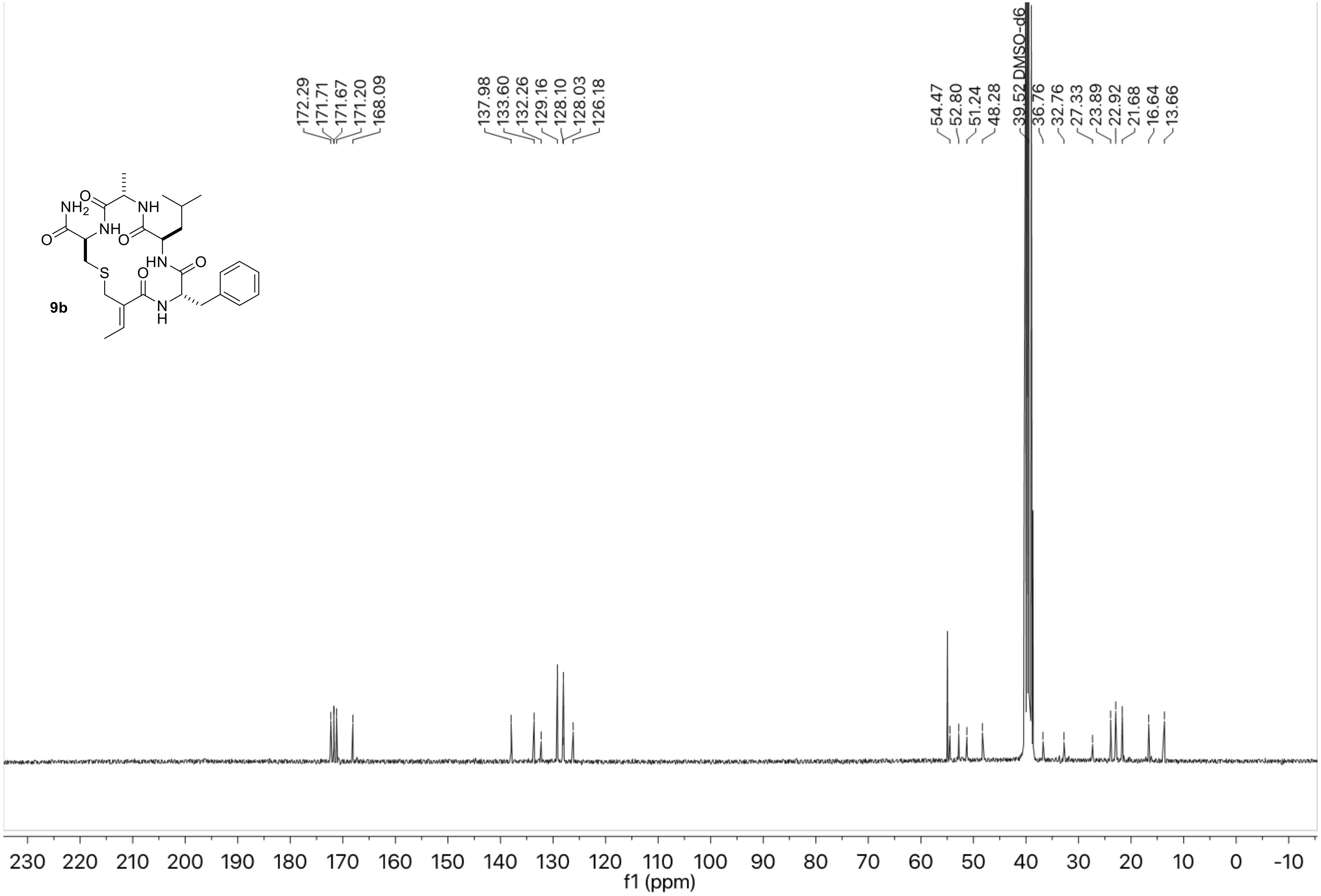

HSQC Spectra of **9b** in DMSO-d_6

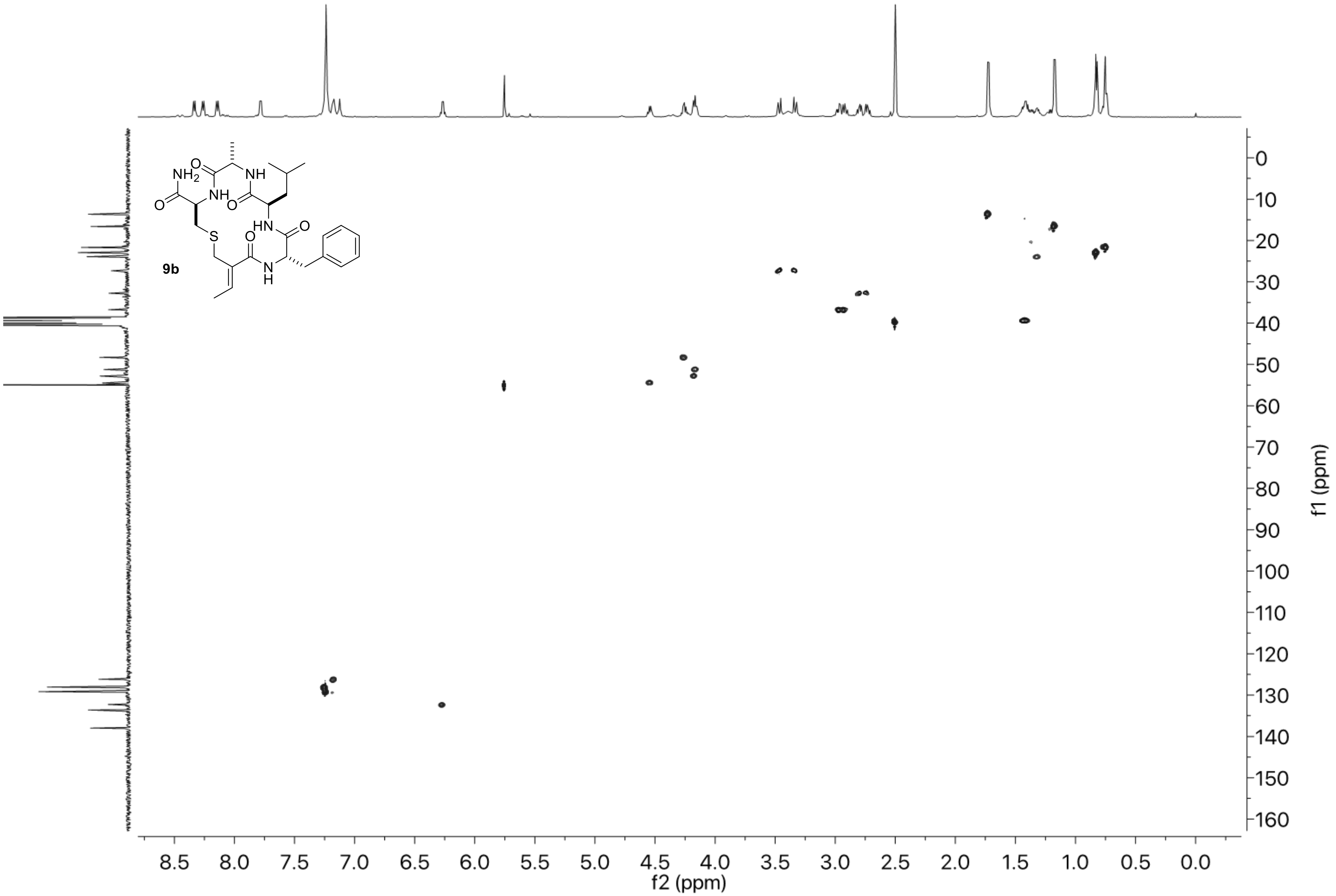

HMBC Spectra of **9b** in DMSO-d_6

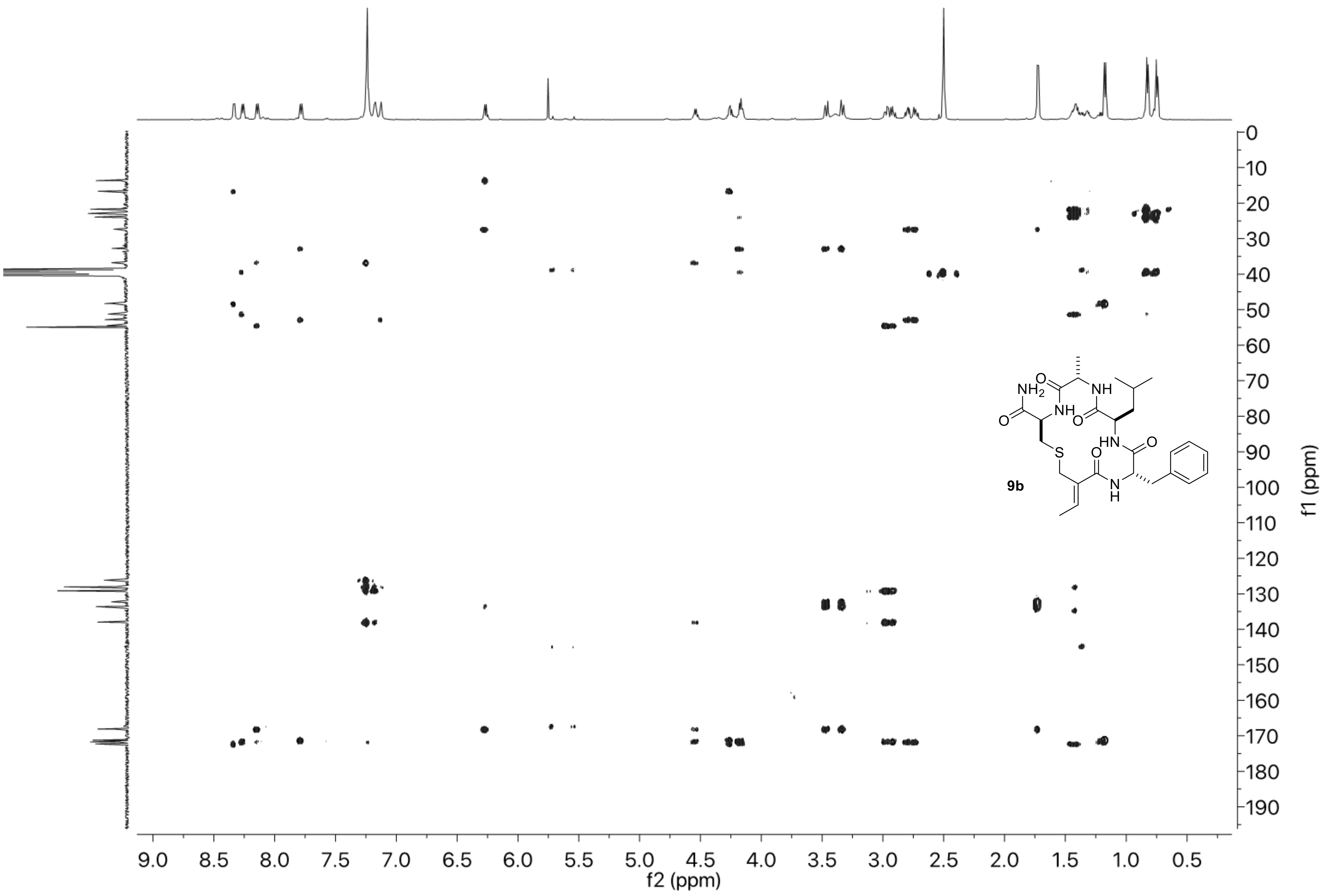

HRMS-ESI-TOF, HRMS/MS spectra, chromatogram and IR-ATR spectrum of **9b**

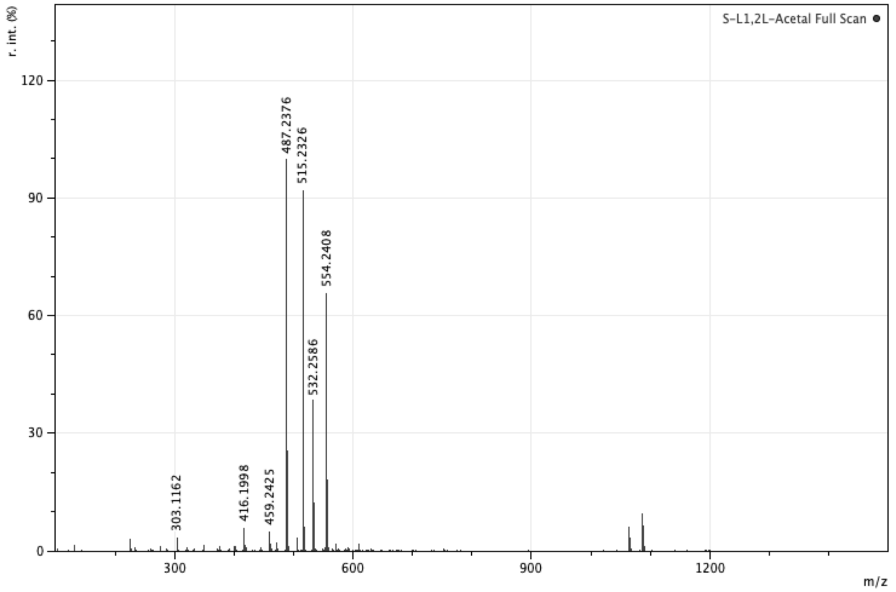

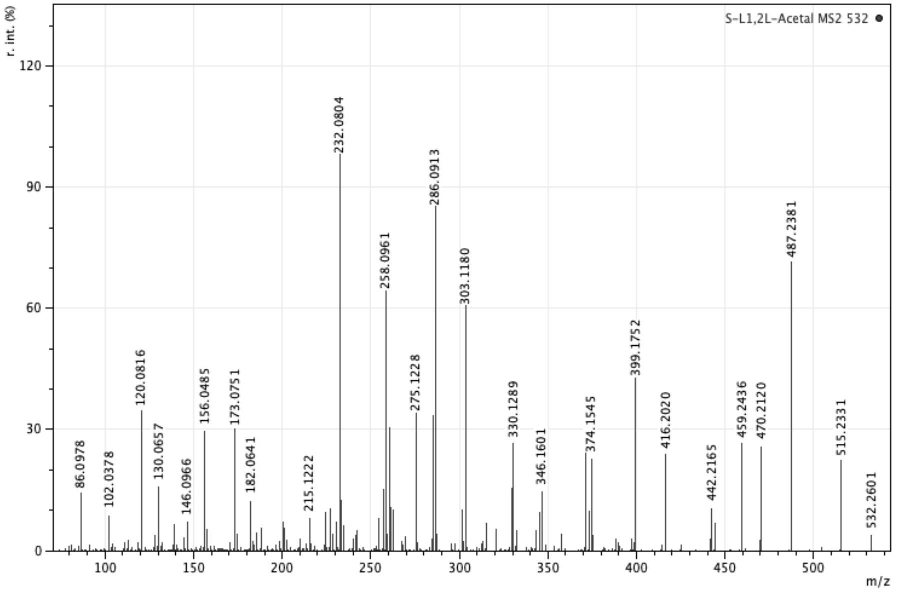

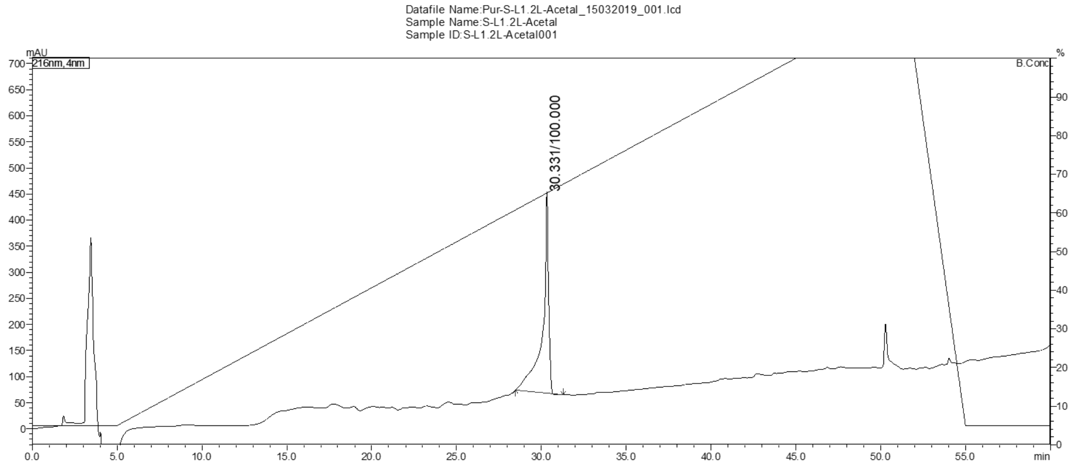

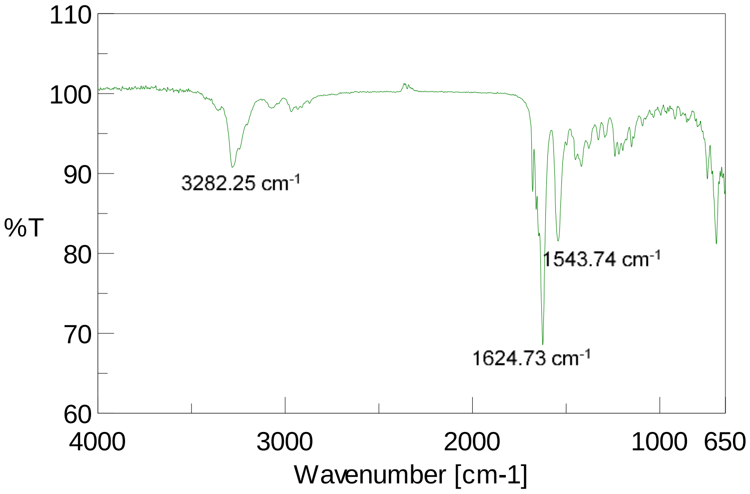

Characterization data for Compound 9c

^{1}H NMR Spectra of **9c** in DMSO-d_6

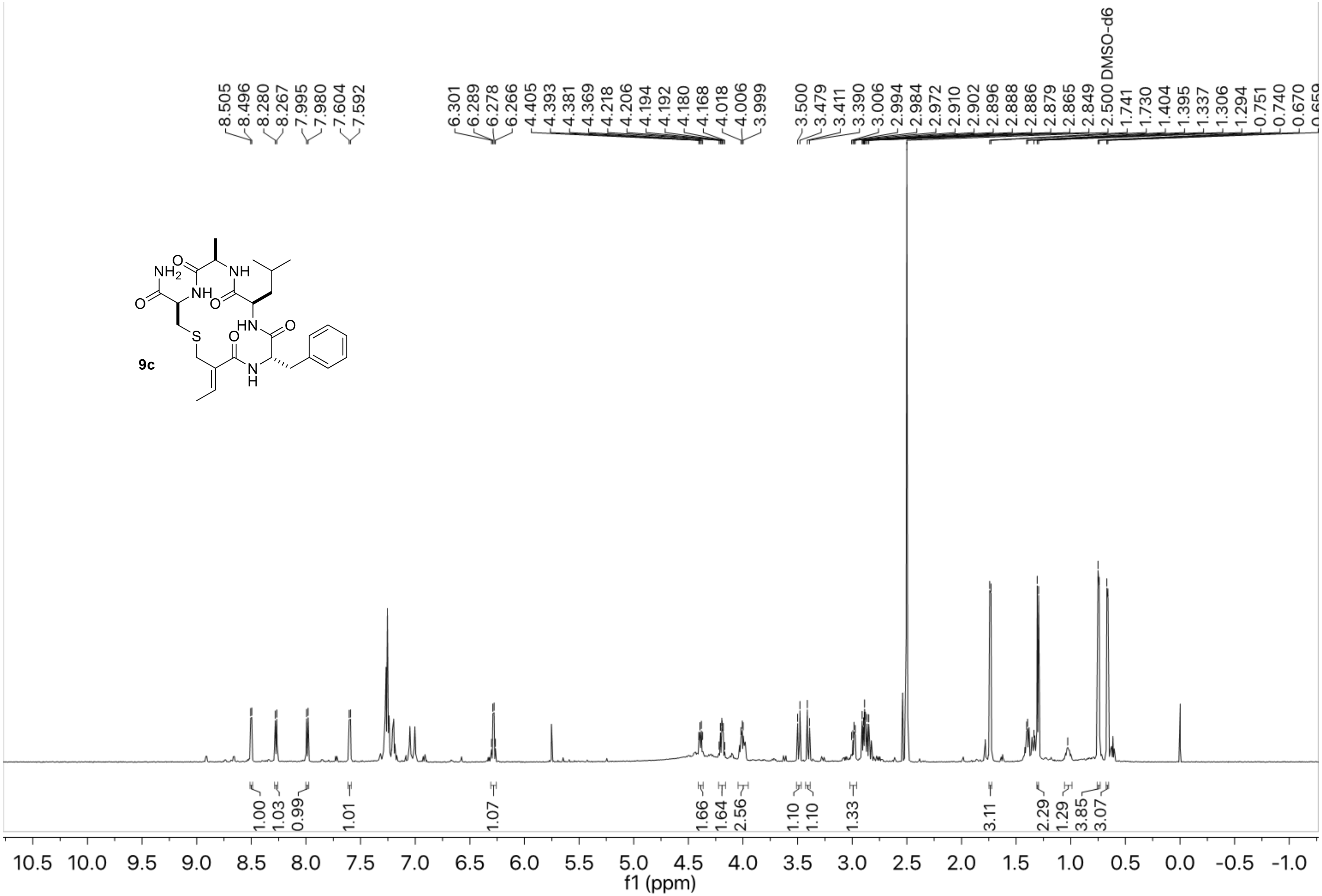

^{13}C NMR Spectra of **9c** in DMSO-d_6

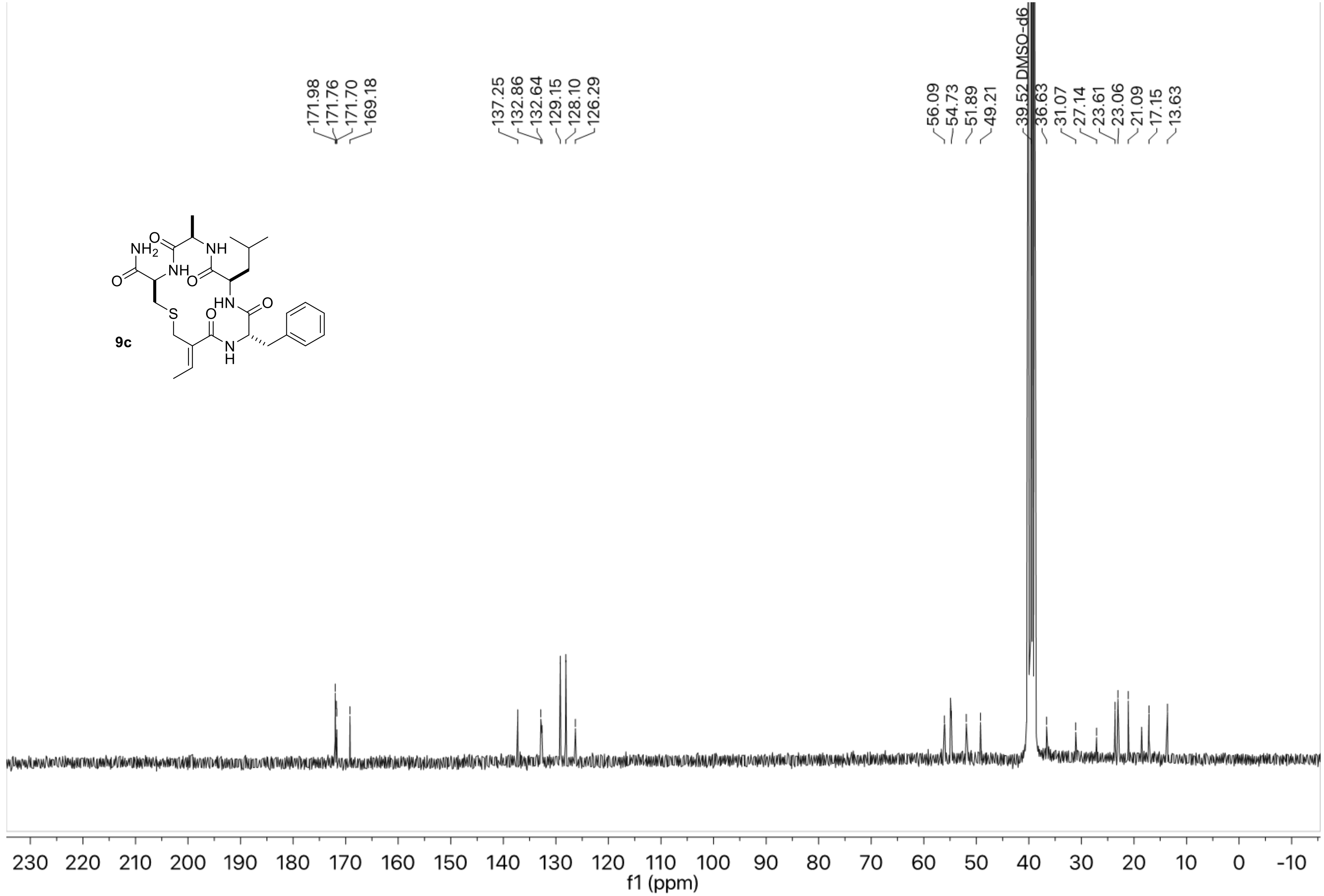

HSQC Spectra of **9c** in DMSO-d_6

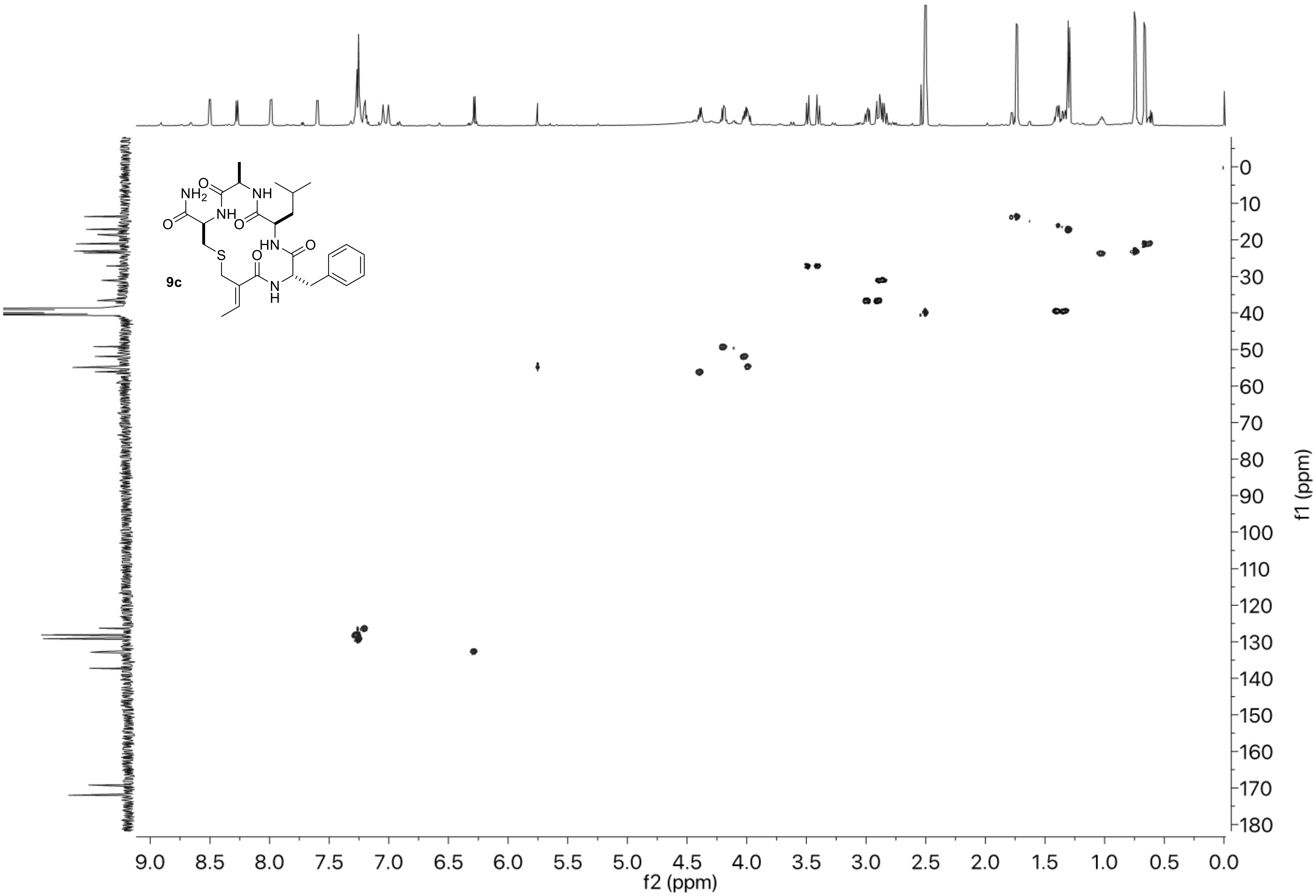

HMBC Spectra of **9c** in DMSO-d_6

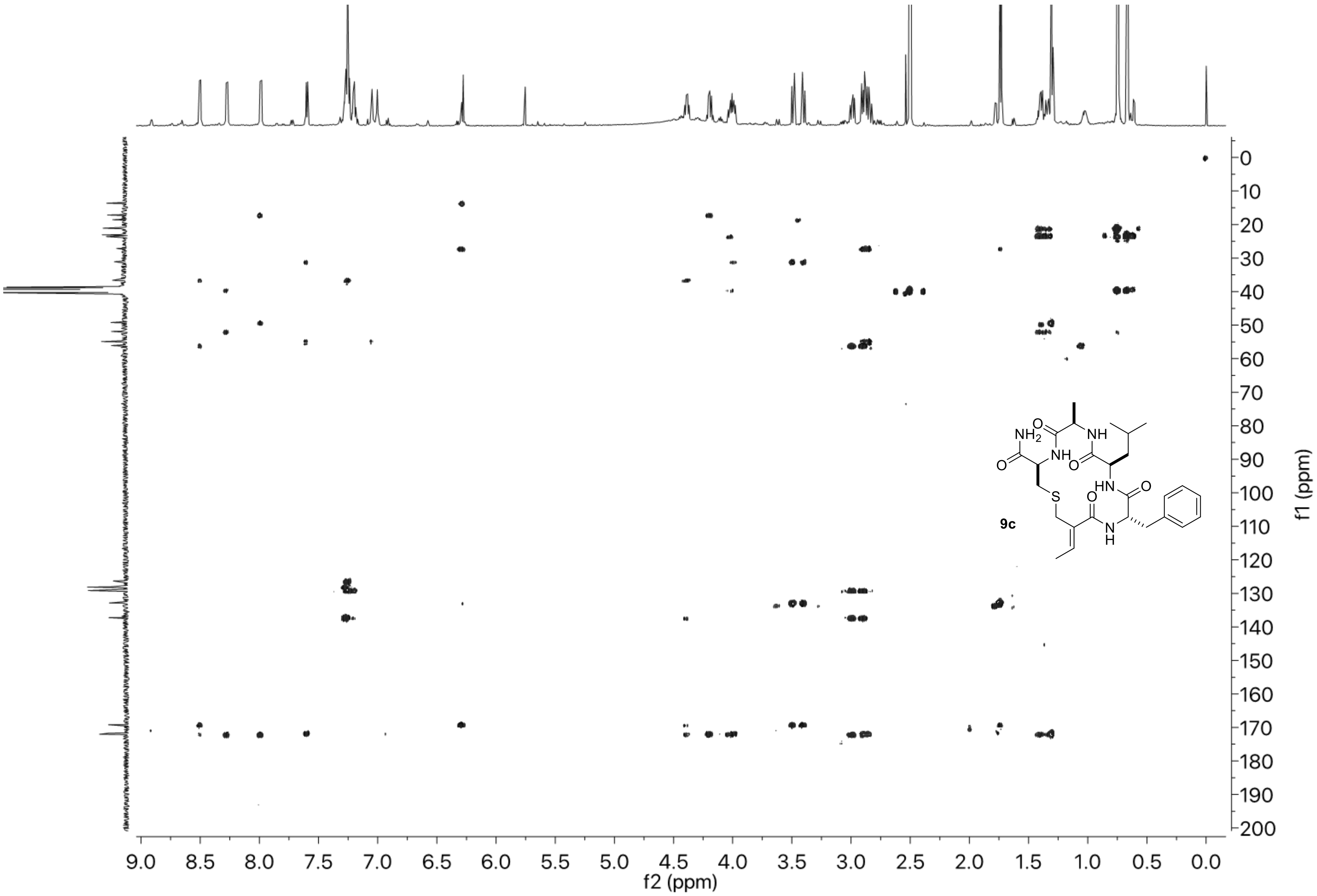

HRMS-ESI-TOF, HRMS/MS spectra, chromatogram and IR-ATR spectrum of **9c**

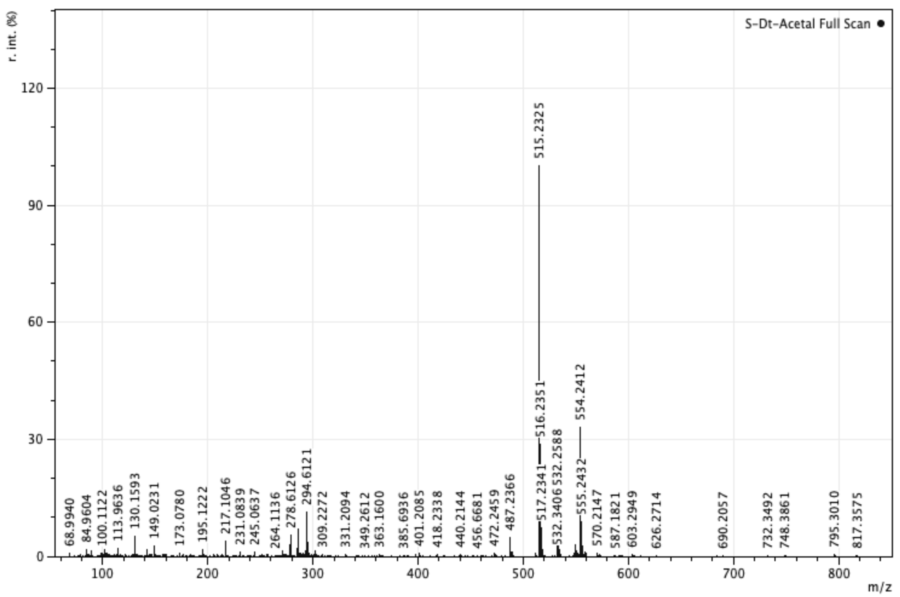

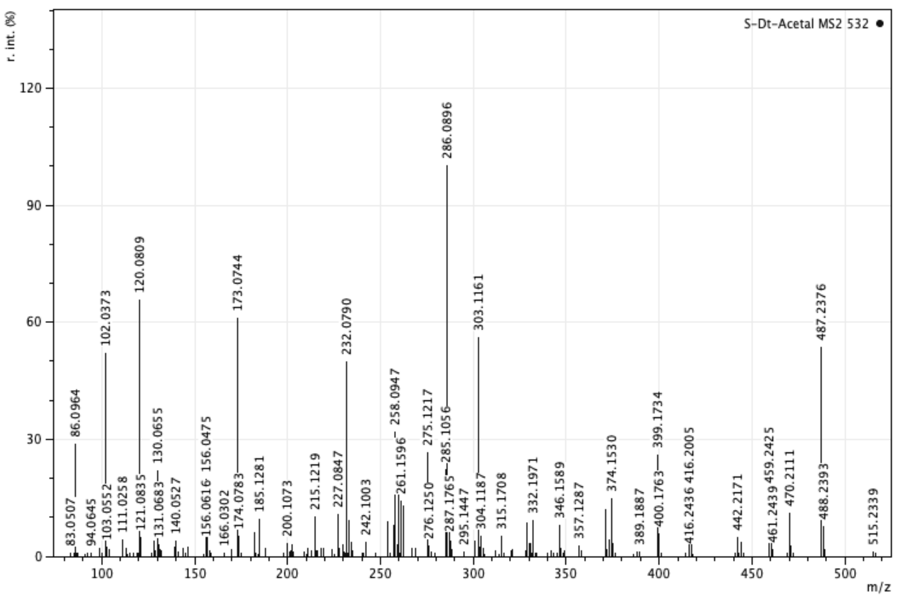

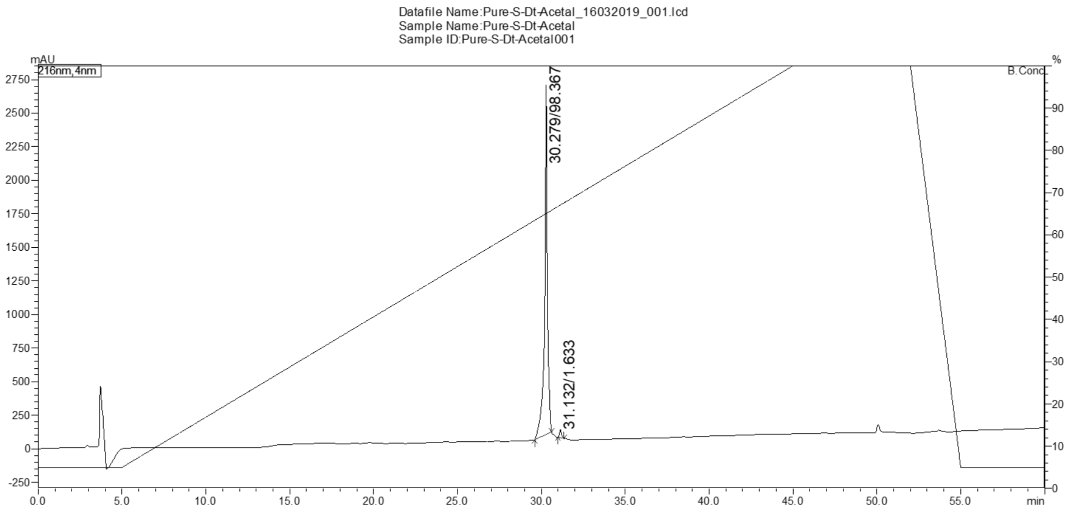

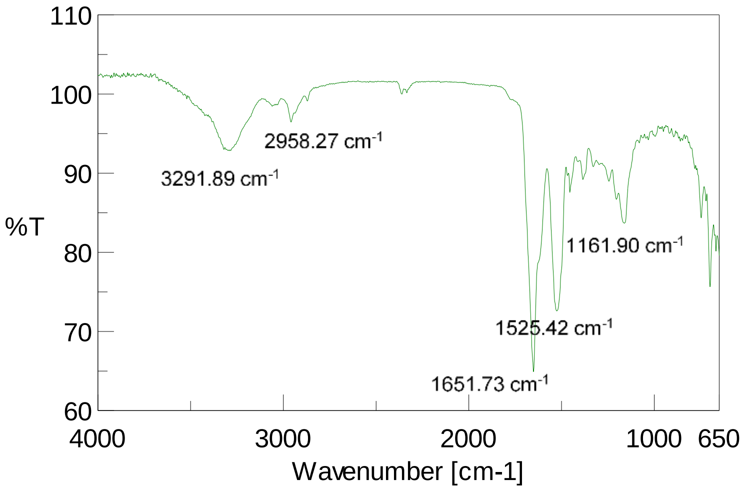

Characterization data for Compound 9d

^{1}H NMR Spectra of **9d** in DMSO-d_6

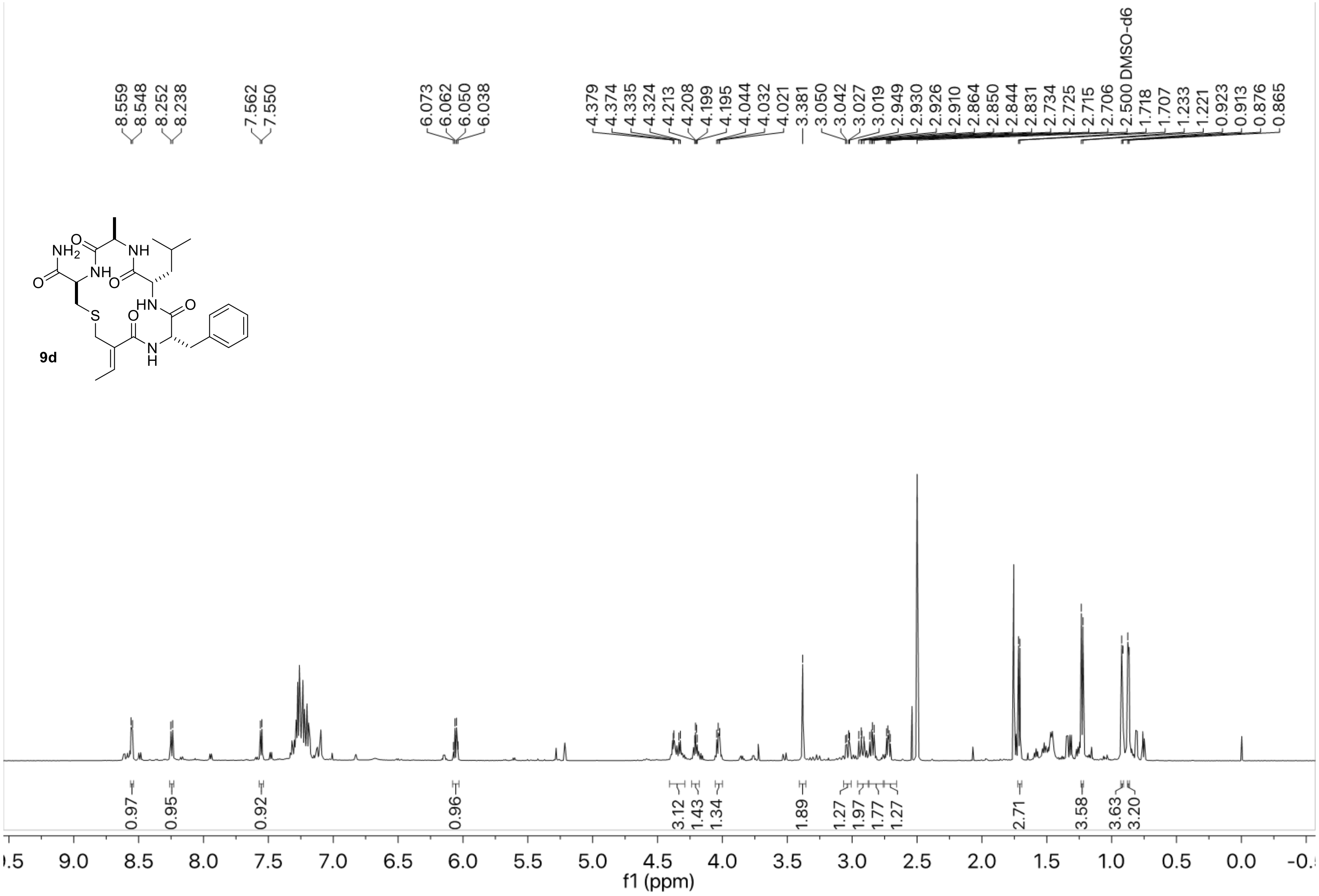

^{13}C NMR Spectra of **9d** in DMSO-d_6

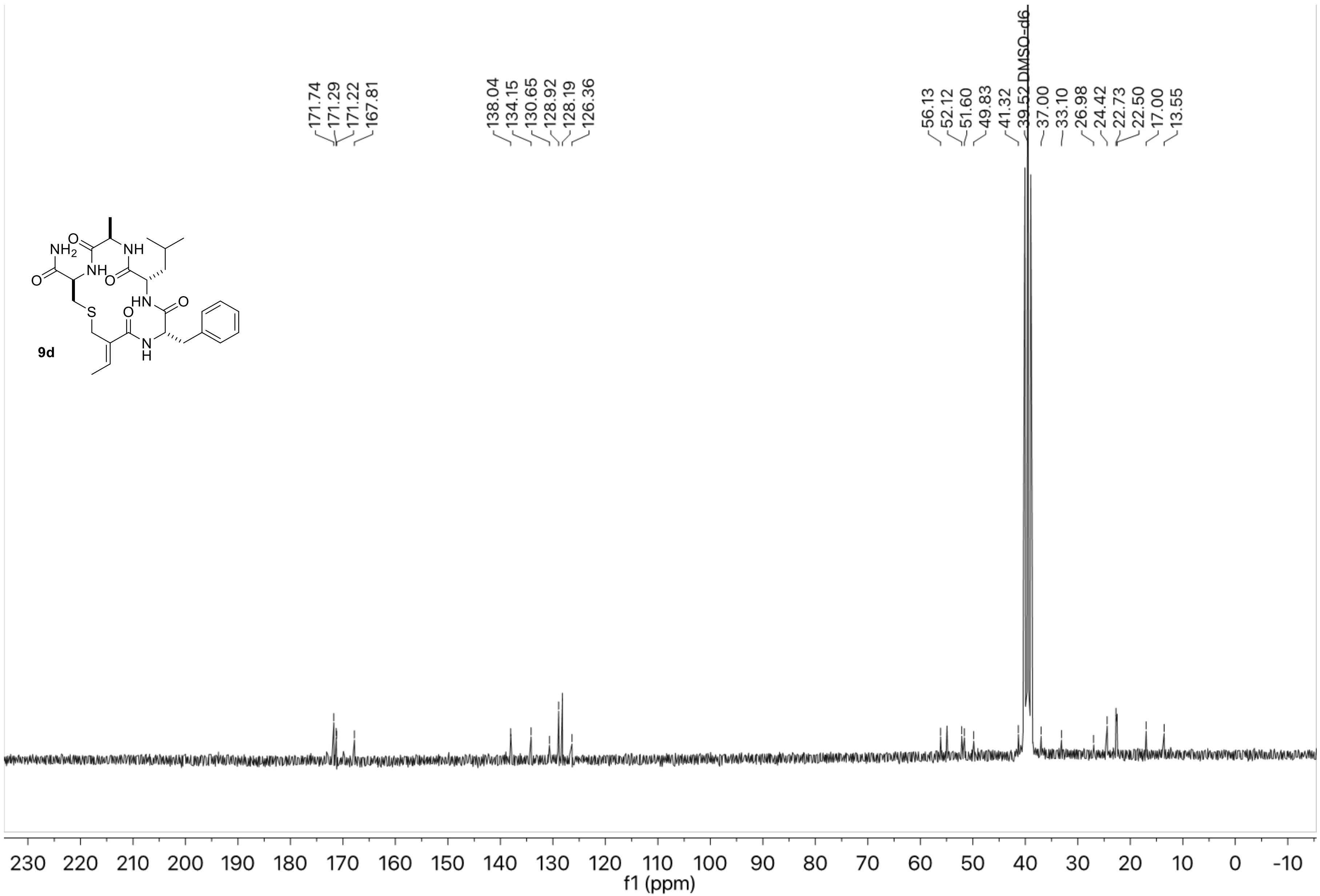

HSQC Spectra of **9d** in DMSO-d_6

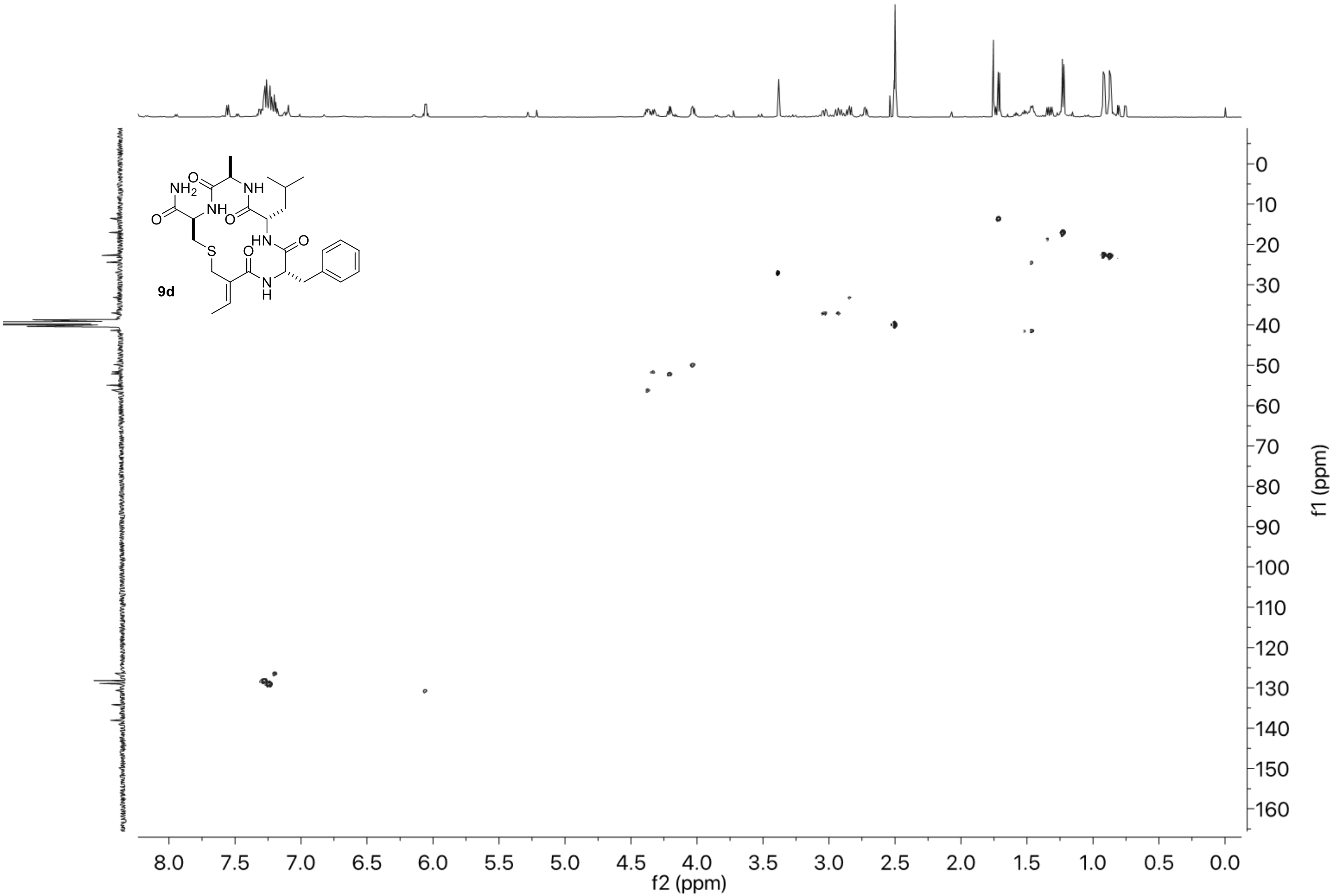

HMBC Spectra of **9d** in DMSO-d_6

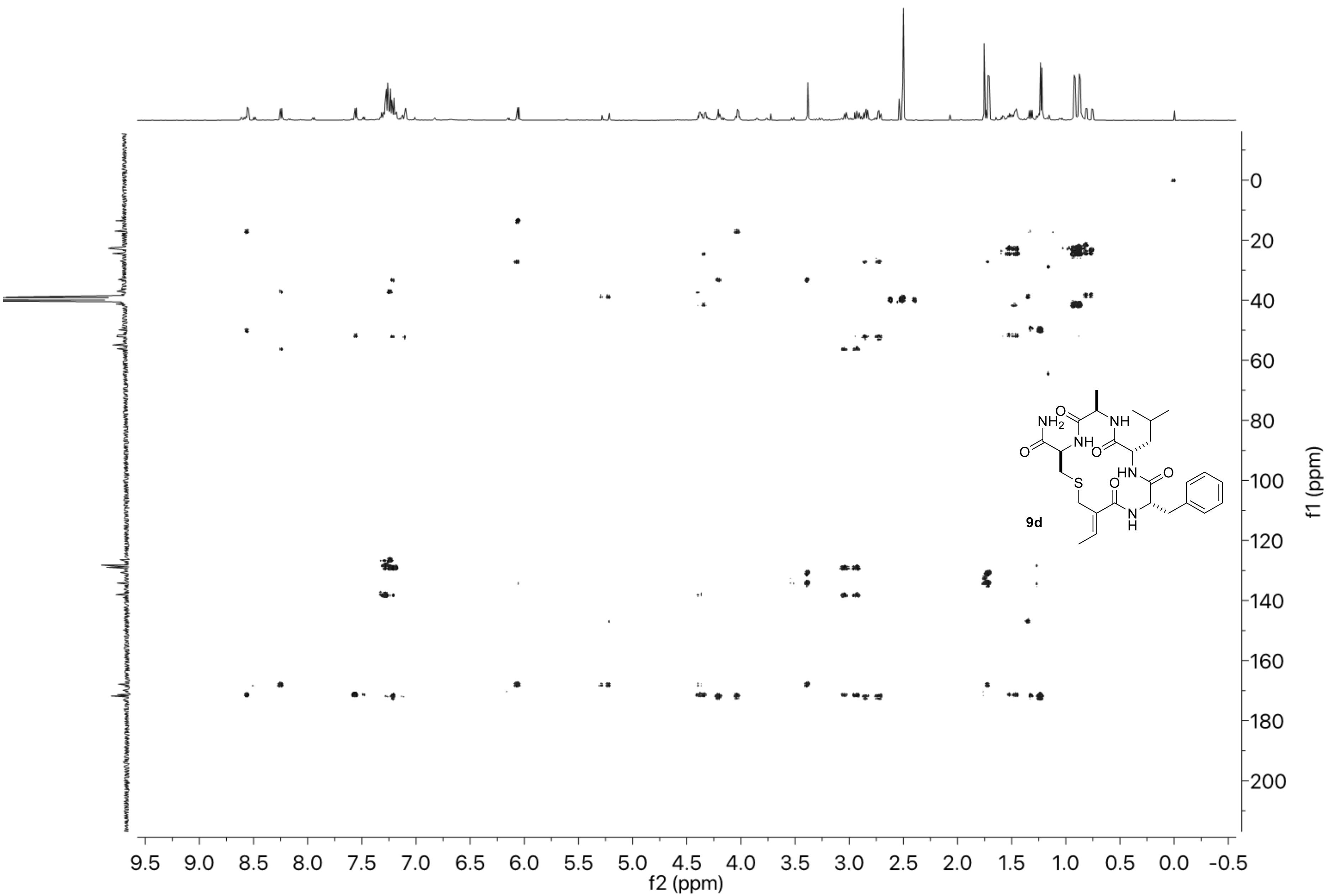

HRMS-ESI-TOF, HRMS/MS spectra, chromatogram and IR-ATR spectrum of **9d**

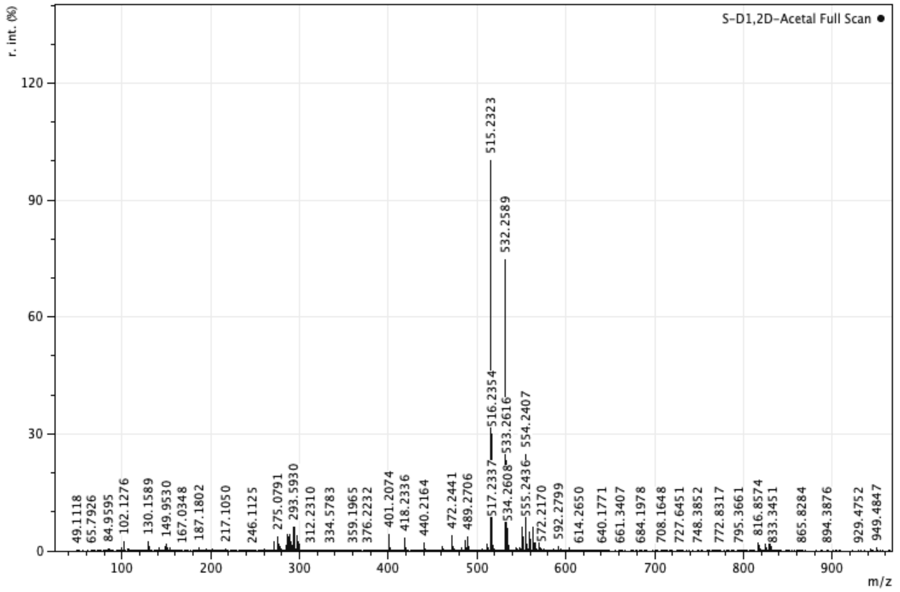

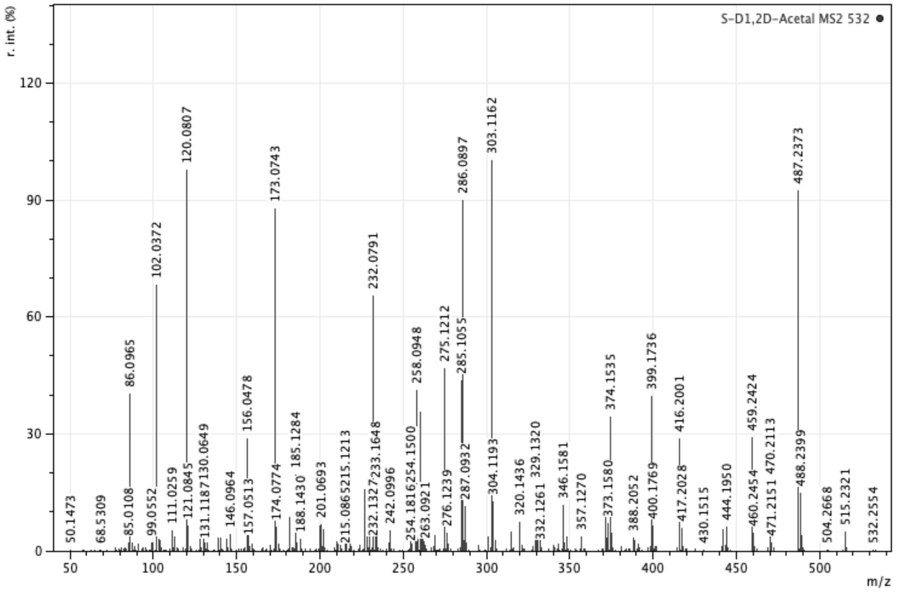

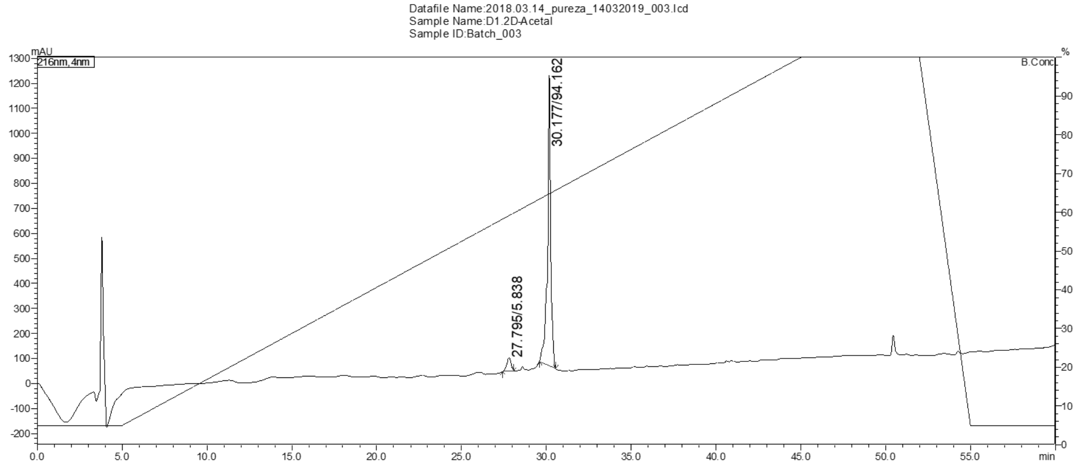

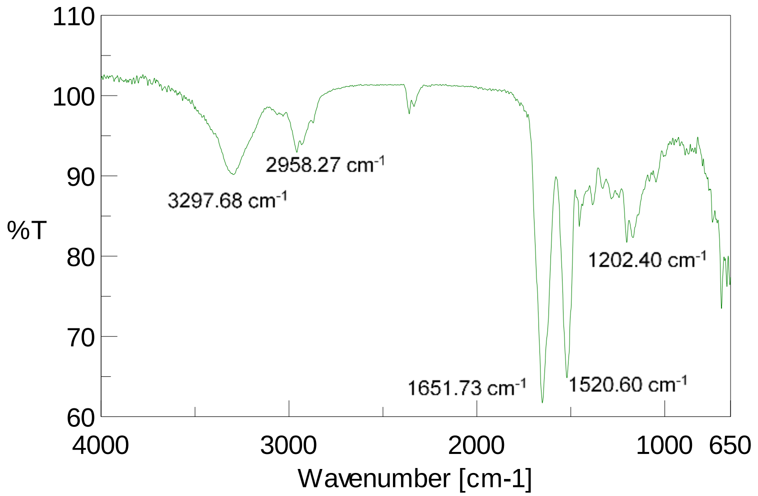

Characteristic fragmentation of compound **9a**, **9b**, **9c** and **9d**.

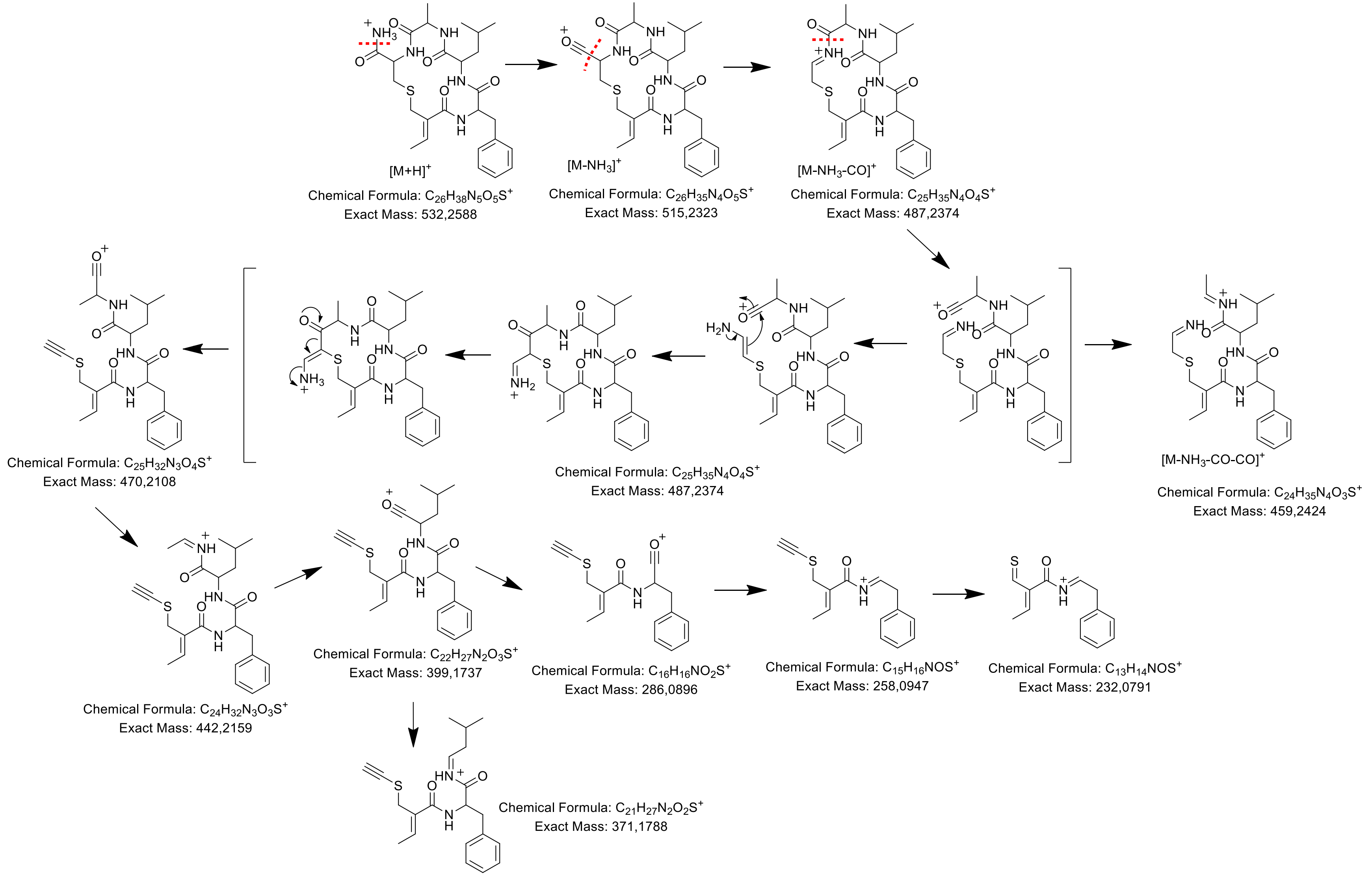

Characterization data for Compound 9e

^{1}H NMR Spectra of **9e** in DMSO-d_6

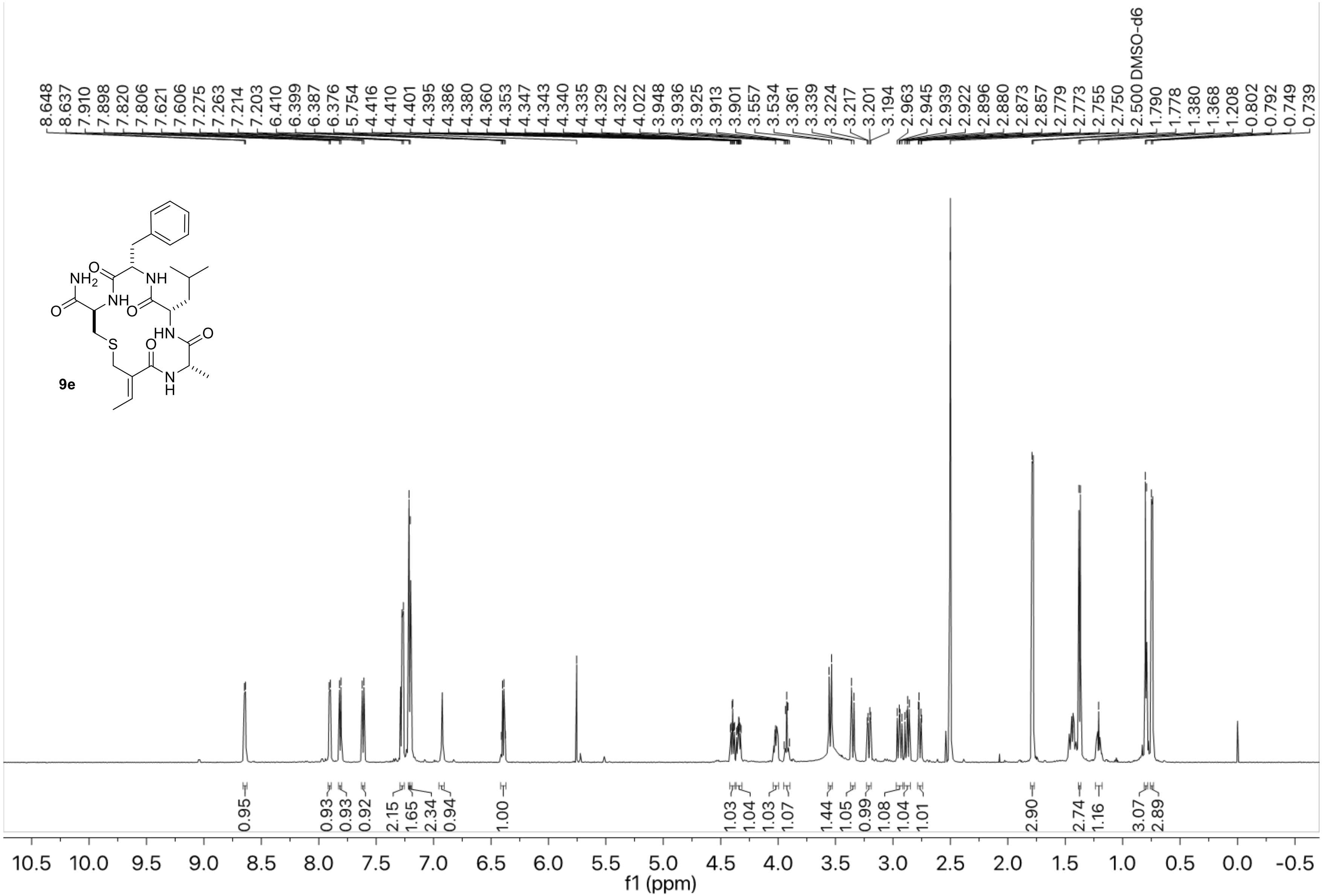

^{13}C NMR Spectra of **9e** in DMSO-d_6

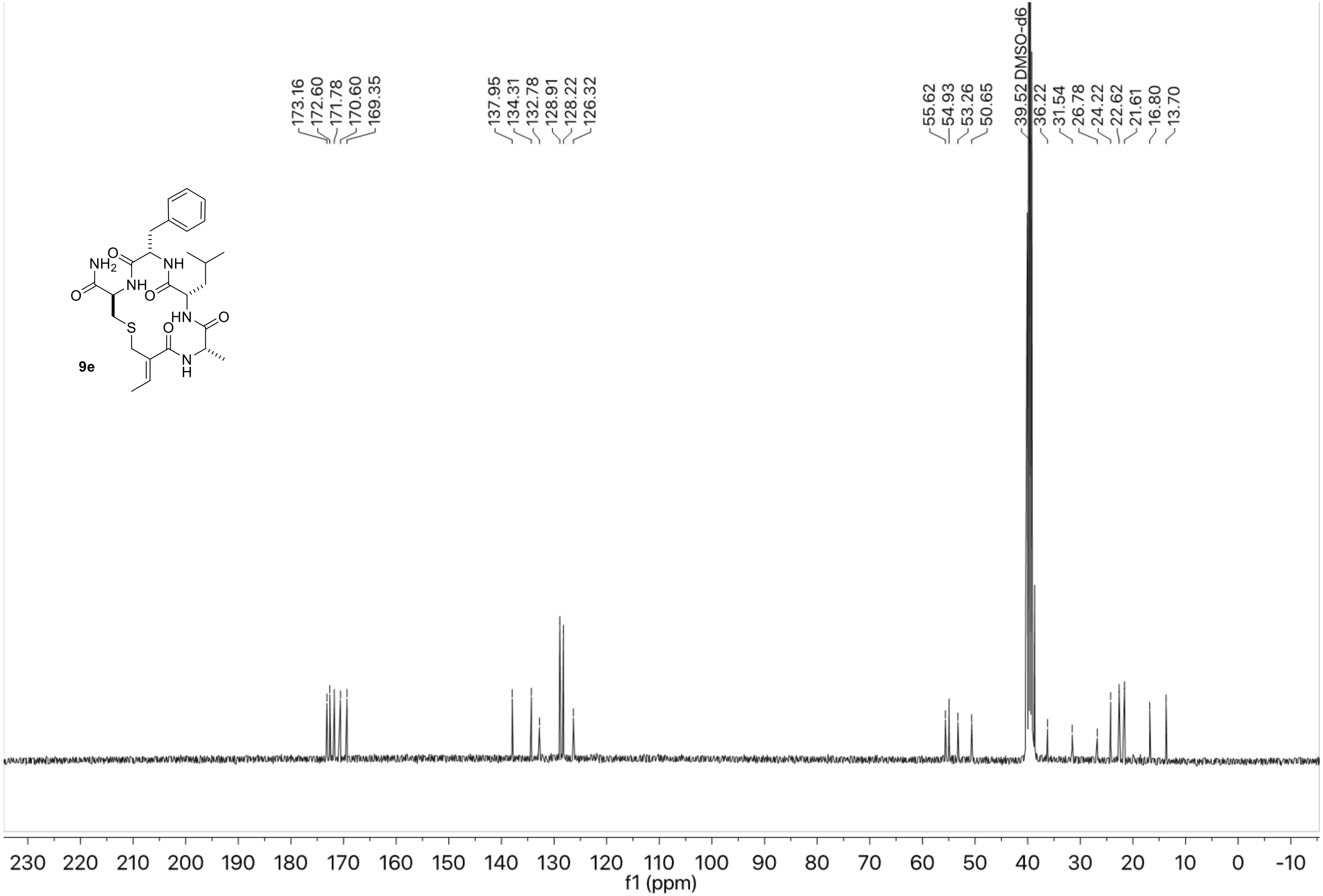

HSQC Spectra of **9e** in DMSO-d_6

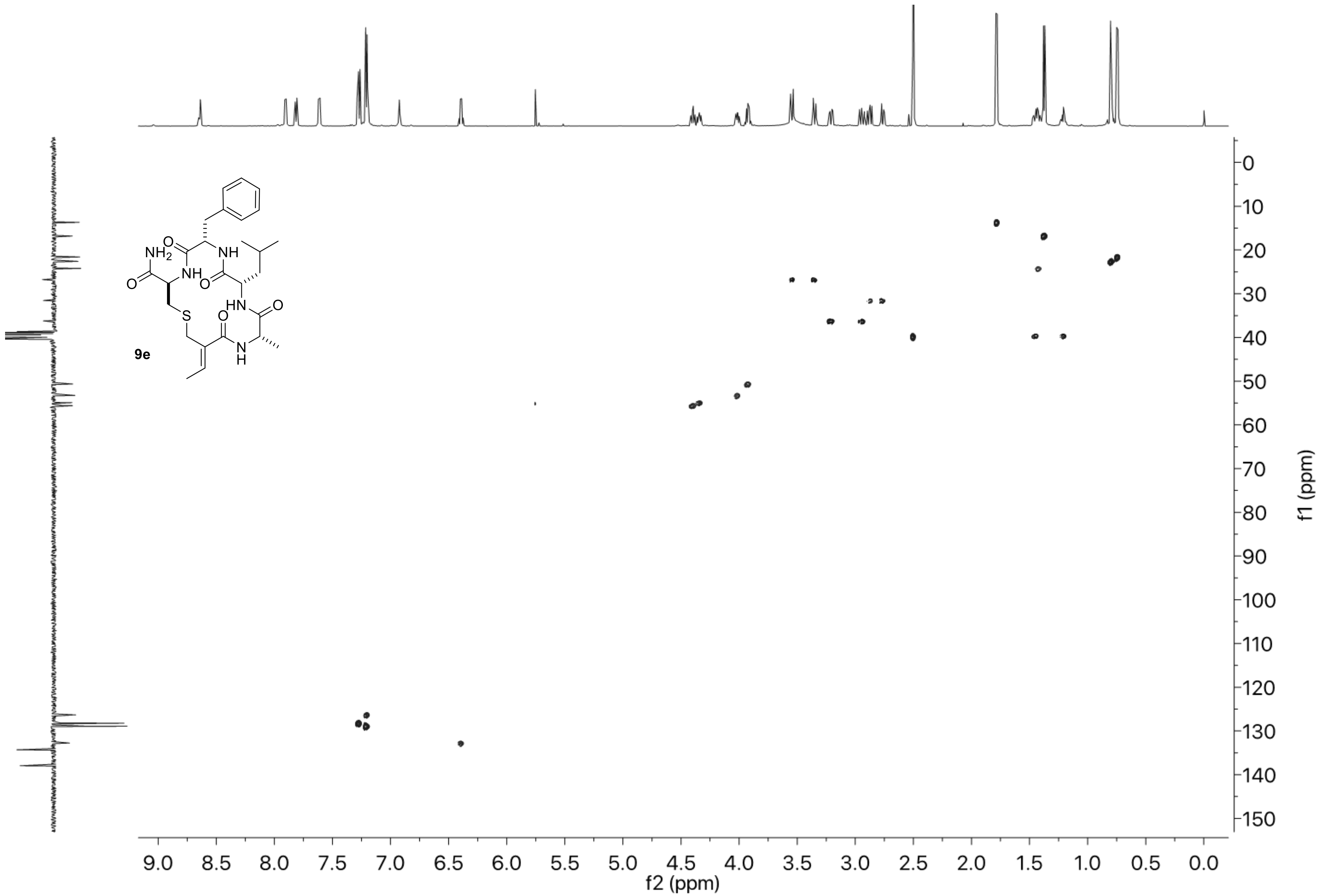

HMBC Spectra of **9e** in DMSO-d_6

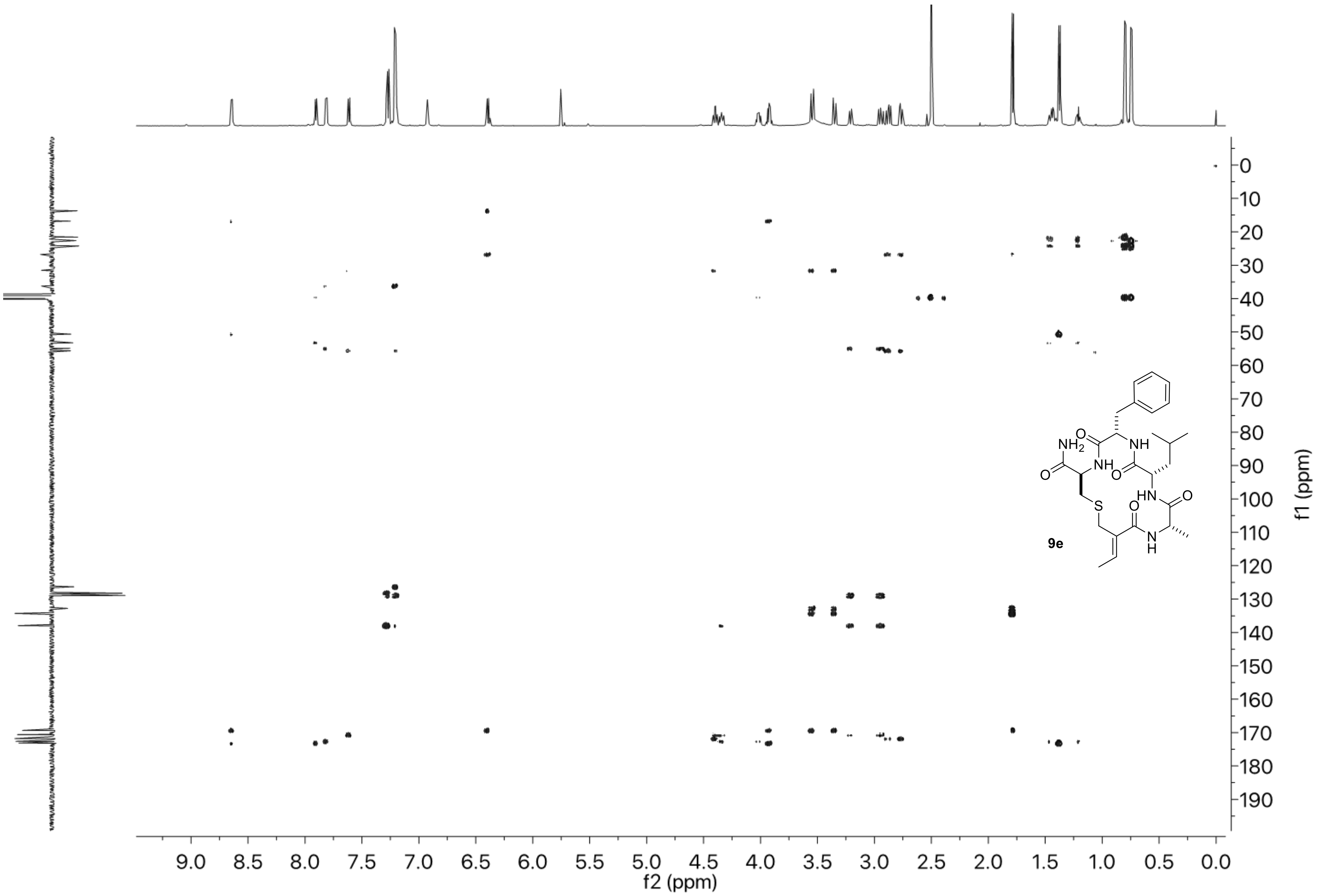

HRMS-ESI-TOF, HRMS/MS spectra, chromatogram and IR-ATR spectrum of **9e**

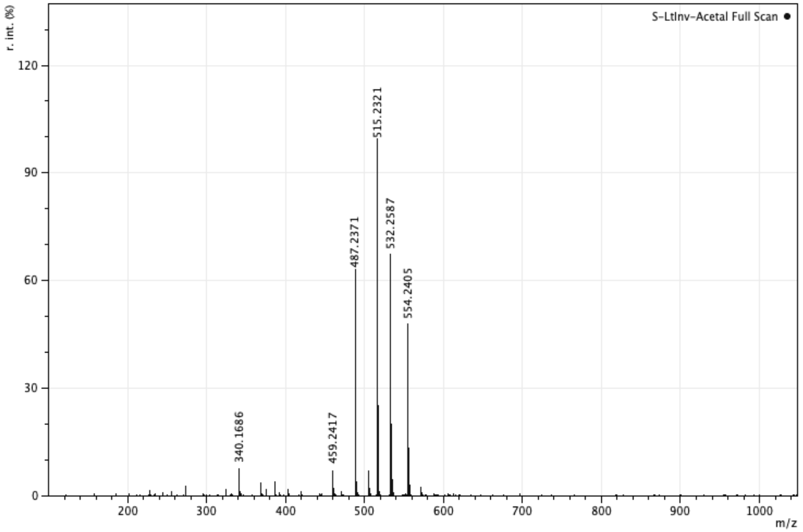

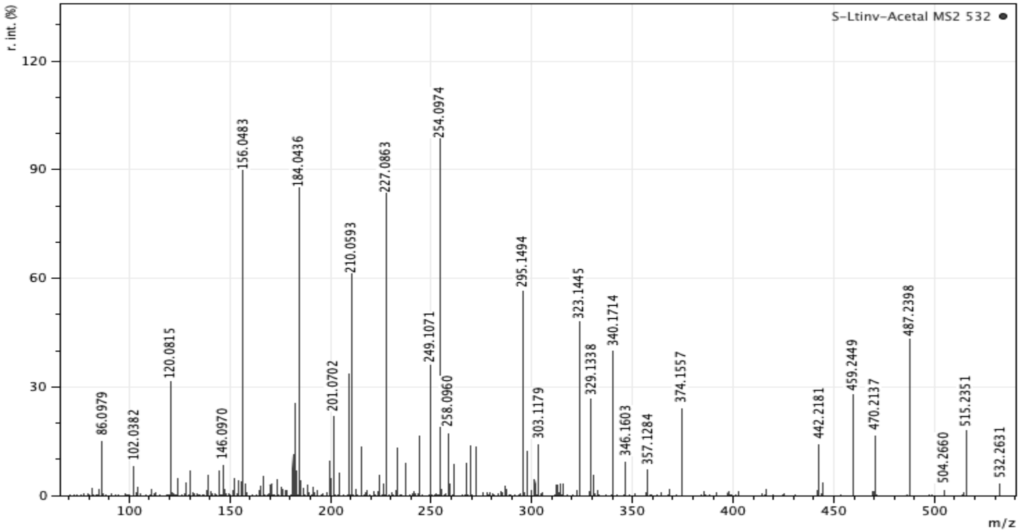

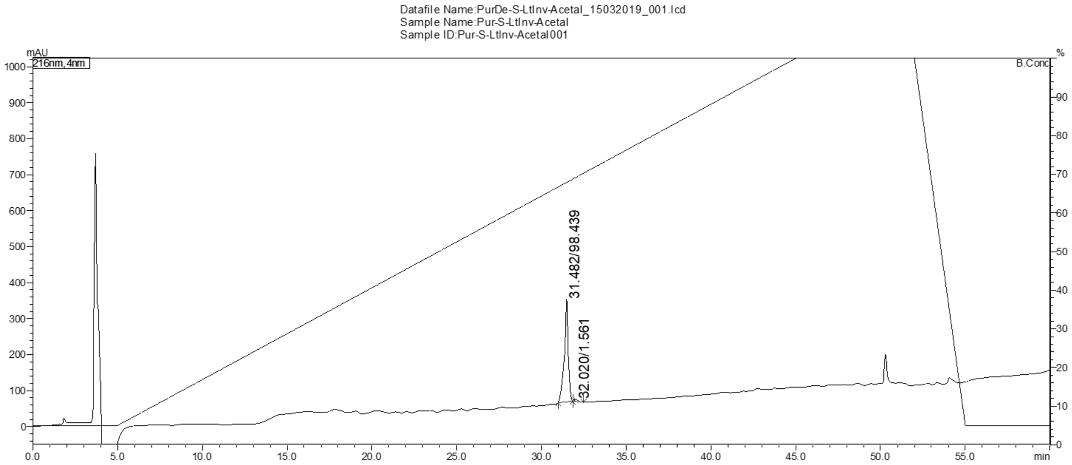

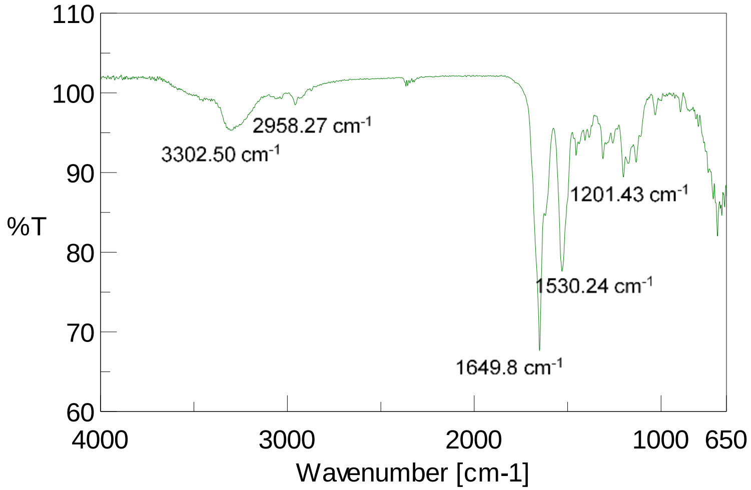

Characteristic fragmentation of compound **9e**

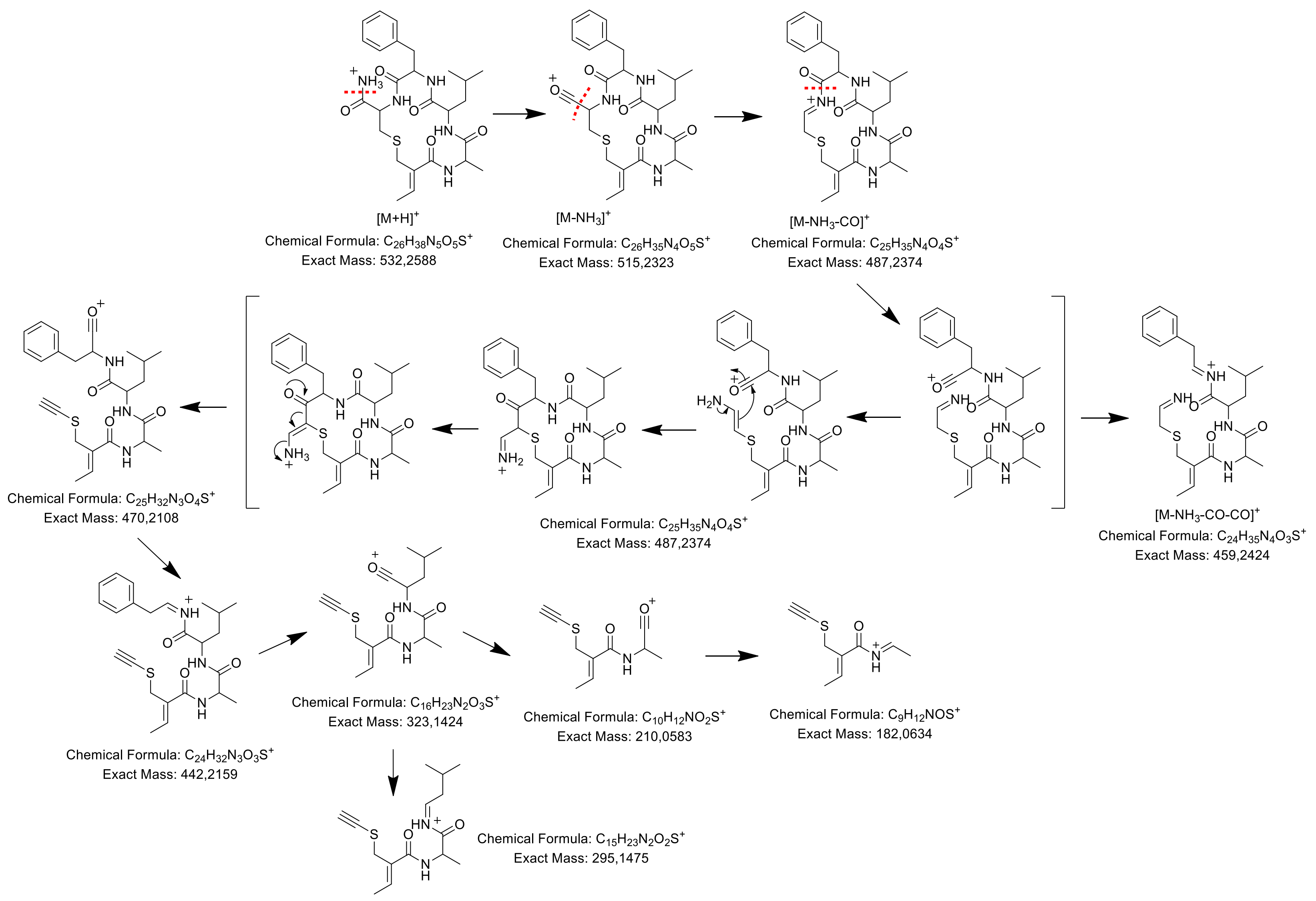

Characterization data for Compound 9f

^{1}H NMR Spectra of **9f** in DMSO-d_6

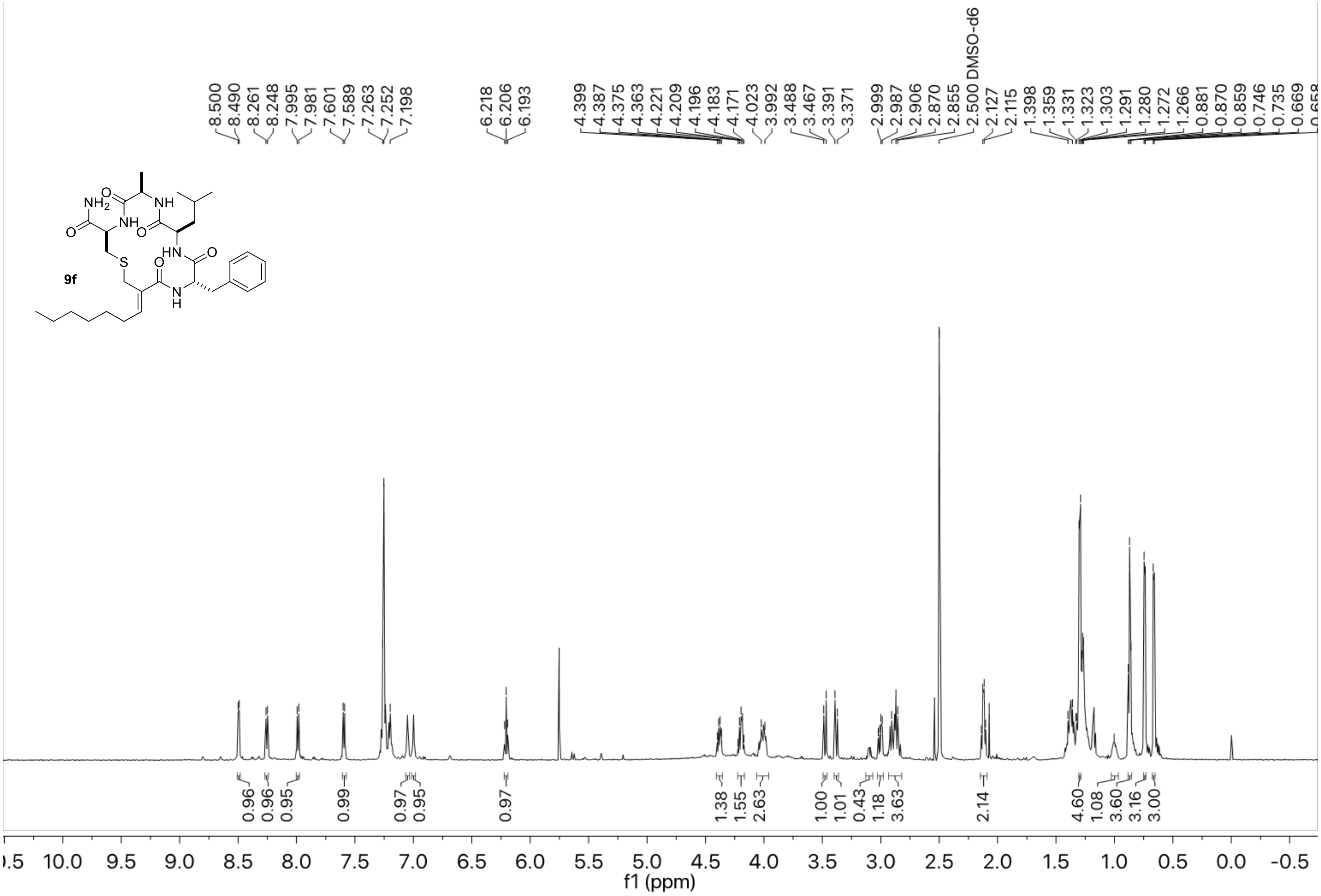

^{13}C NMR Spectra of **9f** in DMSO-d_6

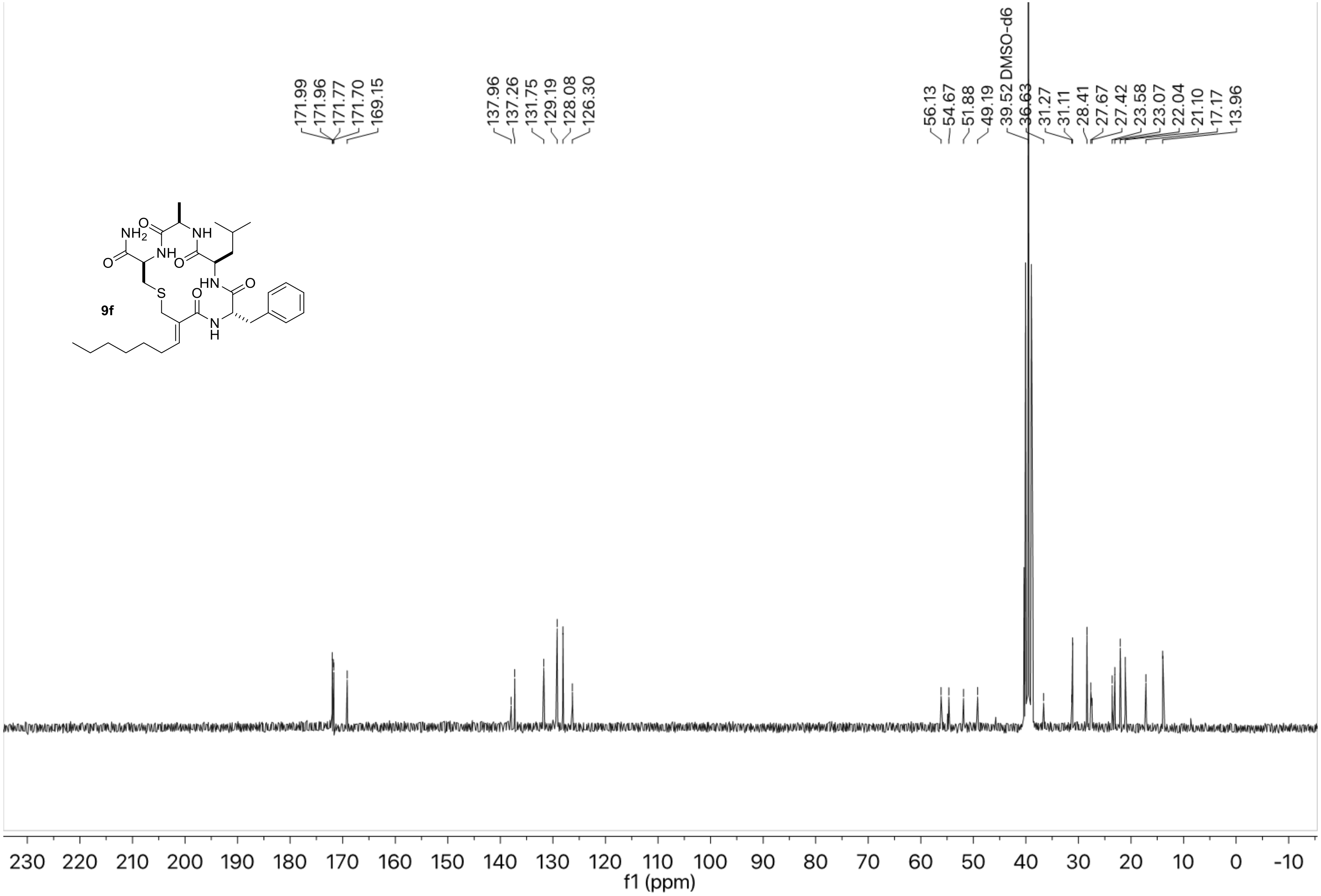

HSQC Spectra of **9f** in DMSO-d_6

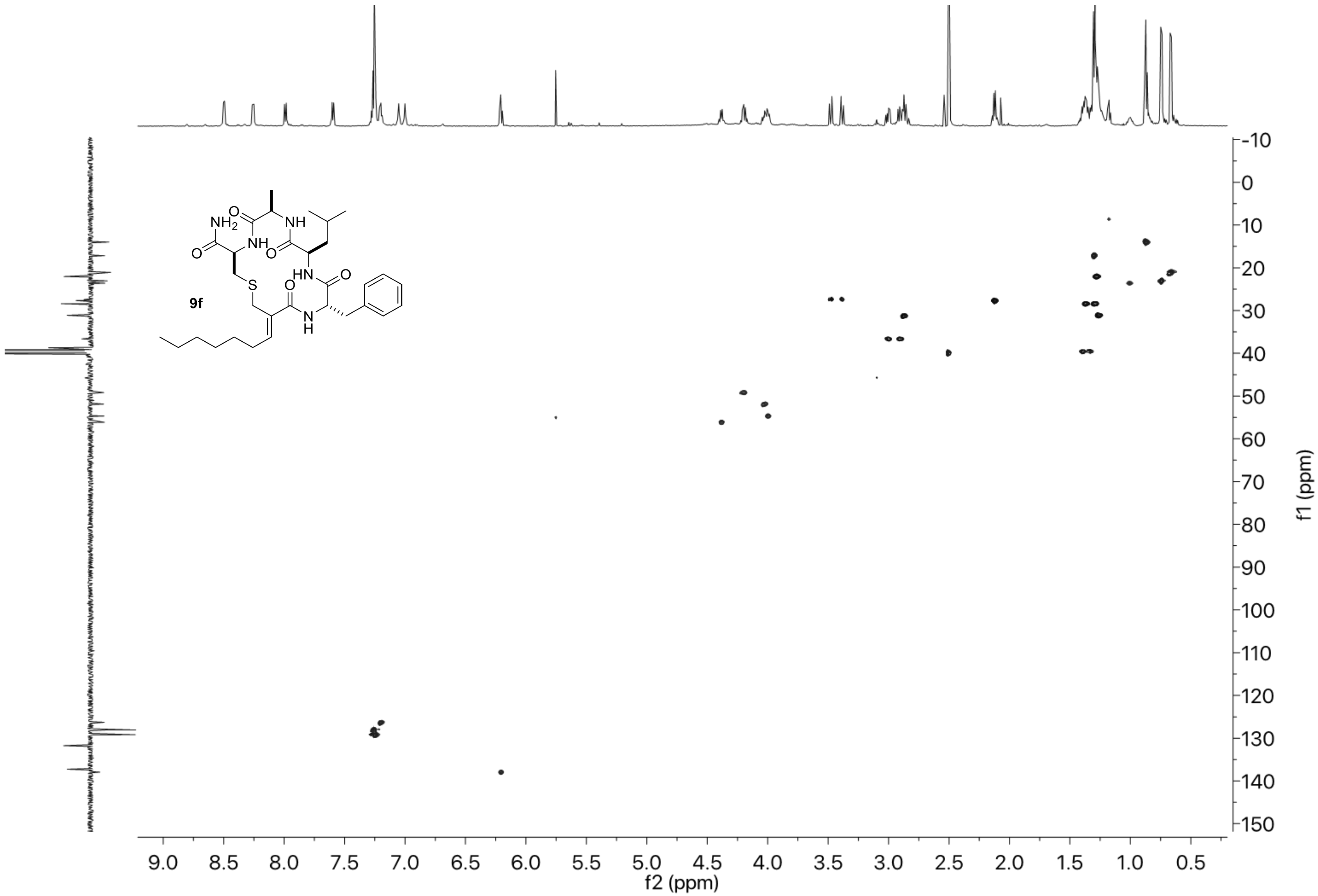

HMBC Spectra of **9f** in DMSO-d_6

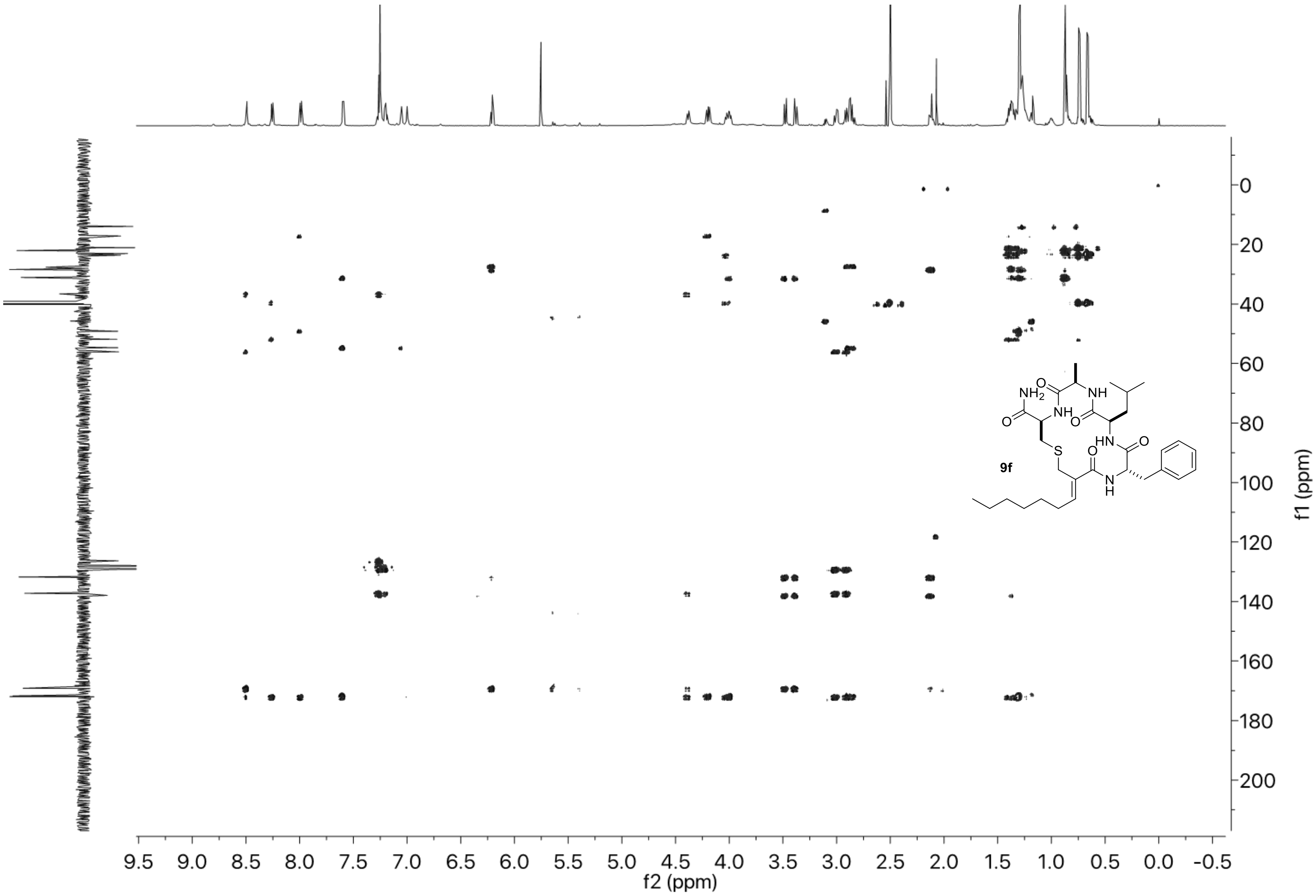

HRMS-ESI-TOF, HRMS/MS spectra, chromatogram and IR-ATR spectrum of **9f**

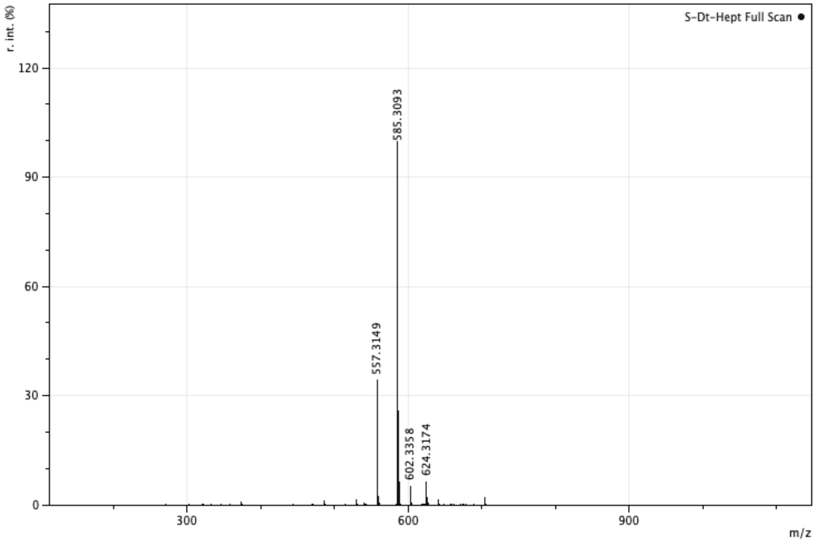

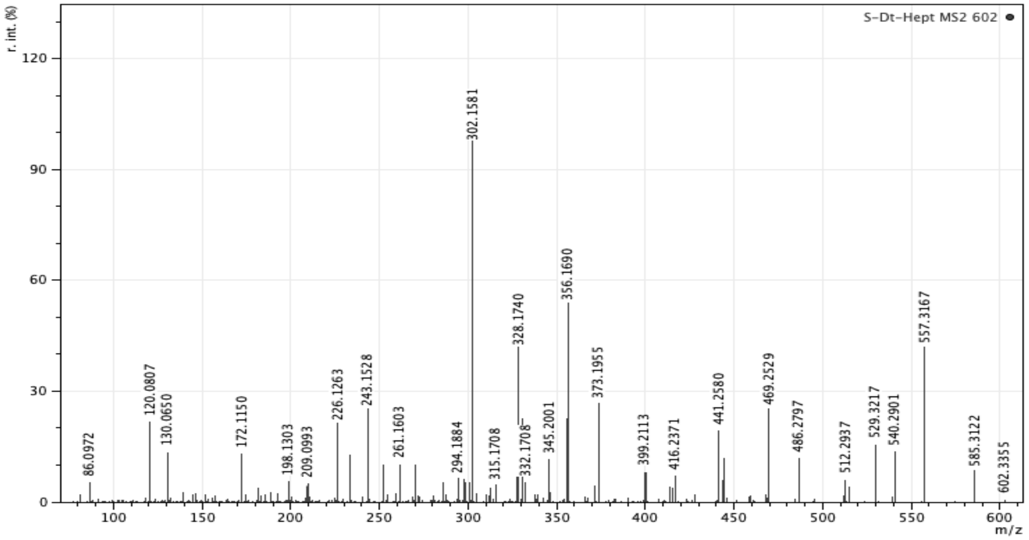

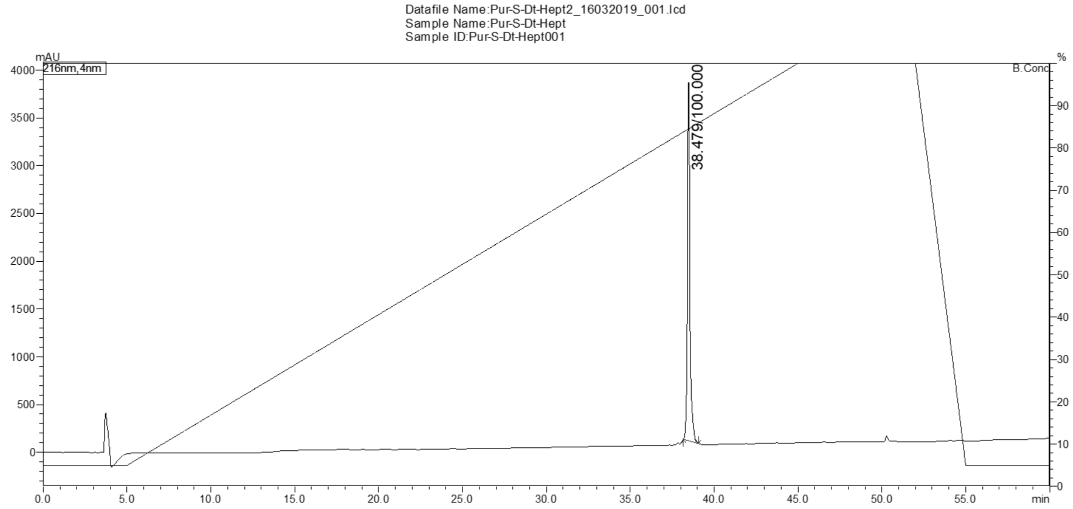

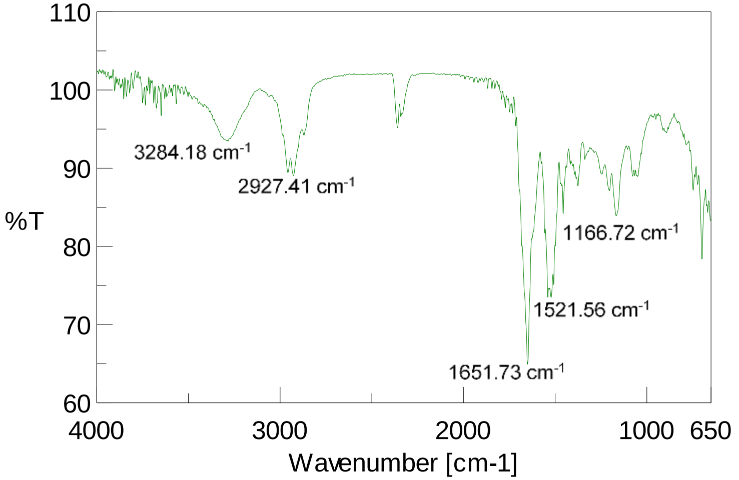

Characterization data for Compound 9g

^{1}H NMR Spectra of **9g** in DMSO-d_6

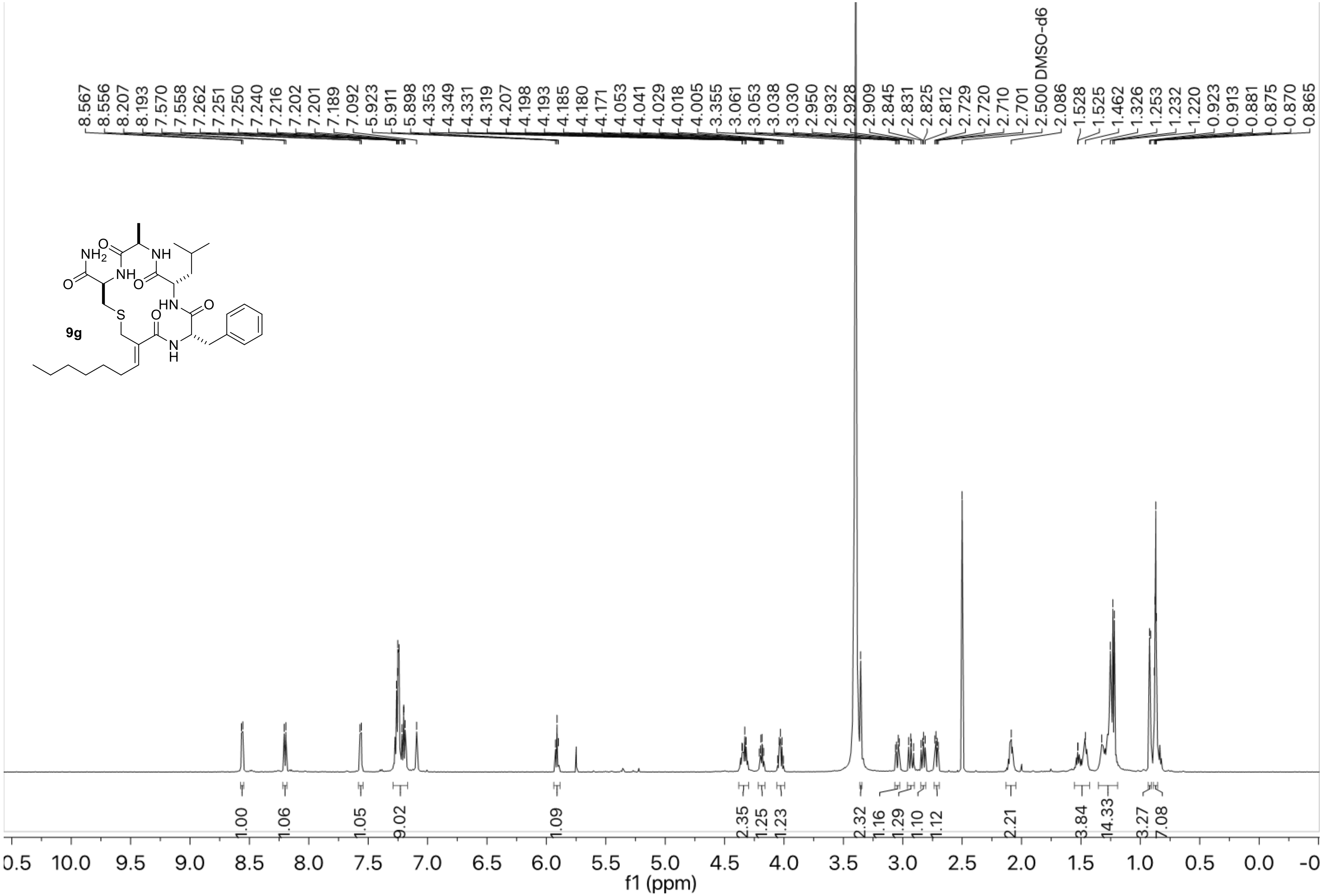

^{13}C NMR Spectra of **9g** in DMSO-d_6

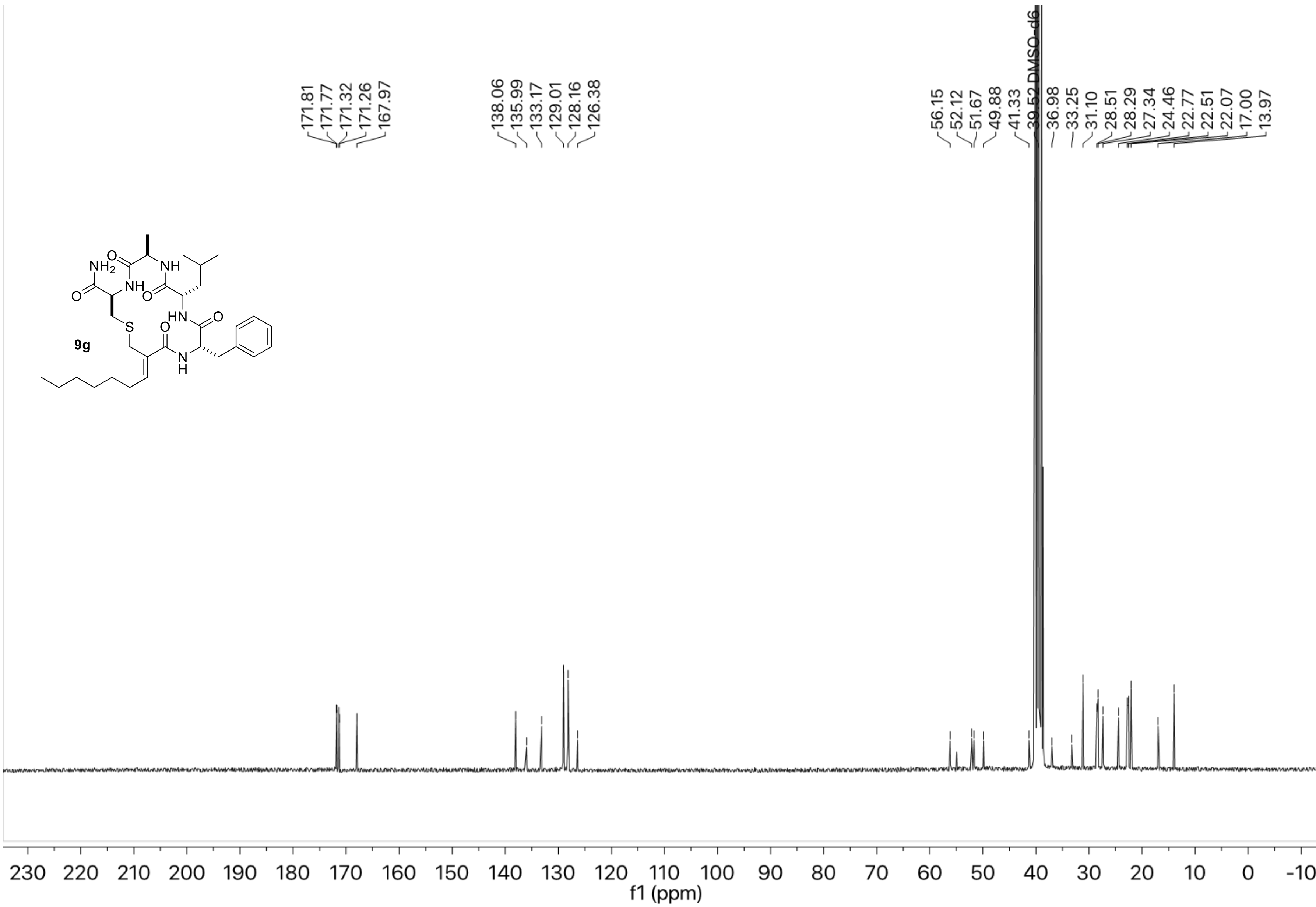

HSQC Spectra of **9g** in DMSO-d_6

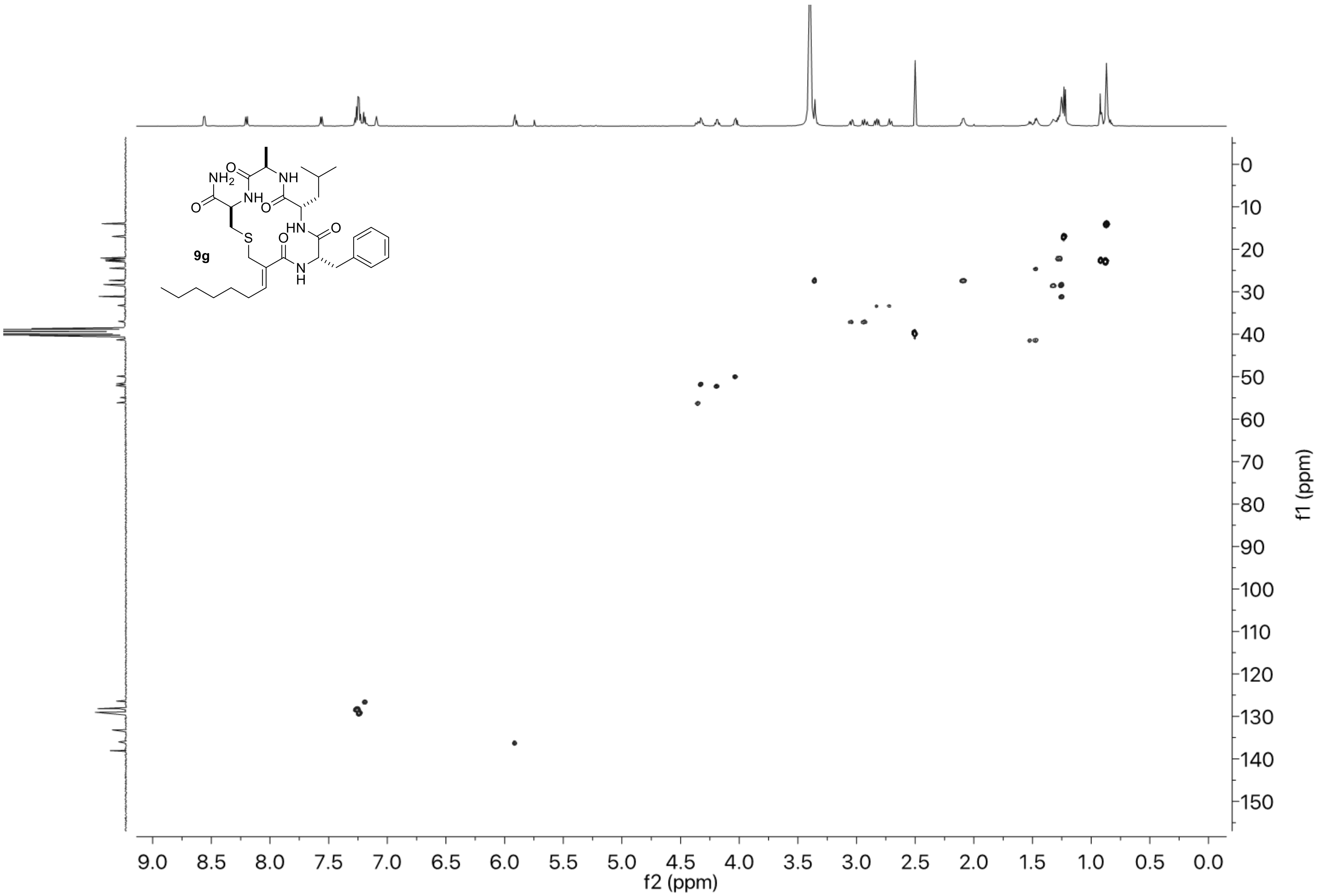

HMBC Spectra of **9g** in DMSO-d_6

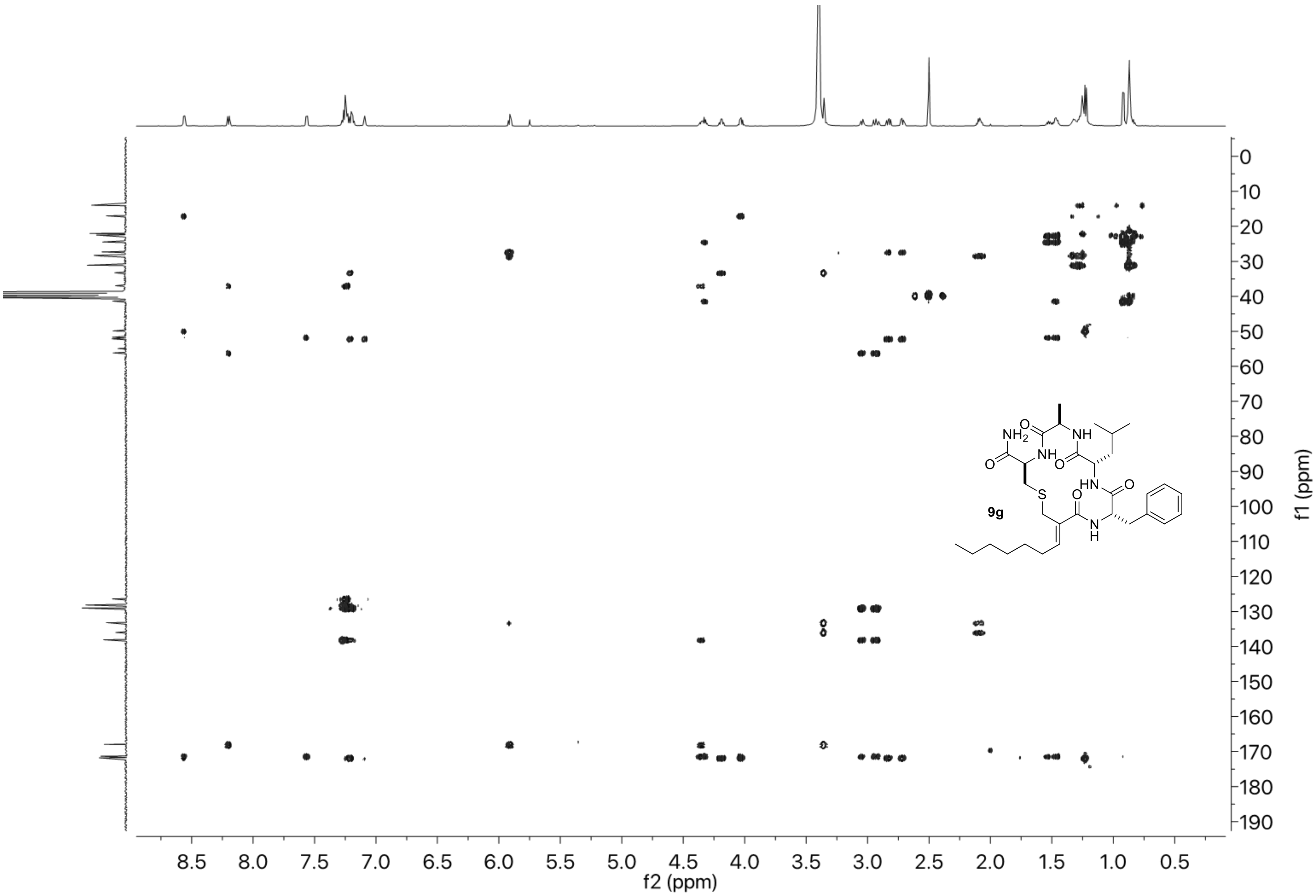

HRMS-ESI-TOF, HRMS/MS spectra, chromatogram and IR-ATR spectrum of **9g**

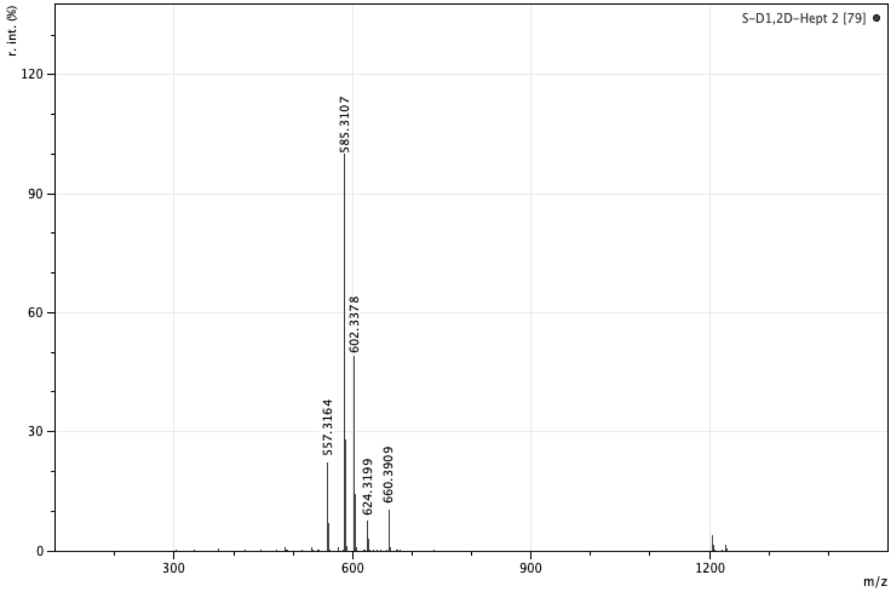

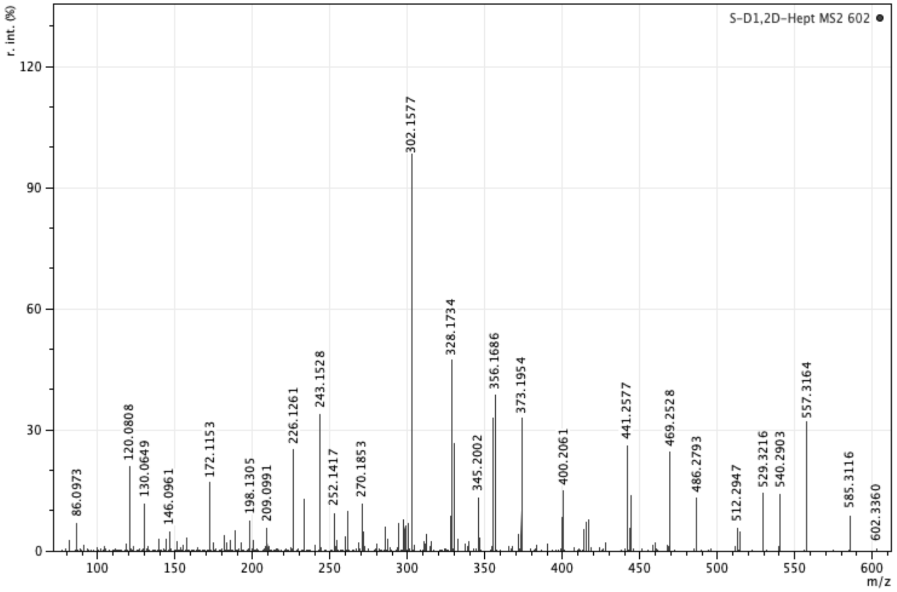

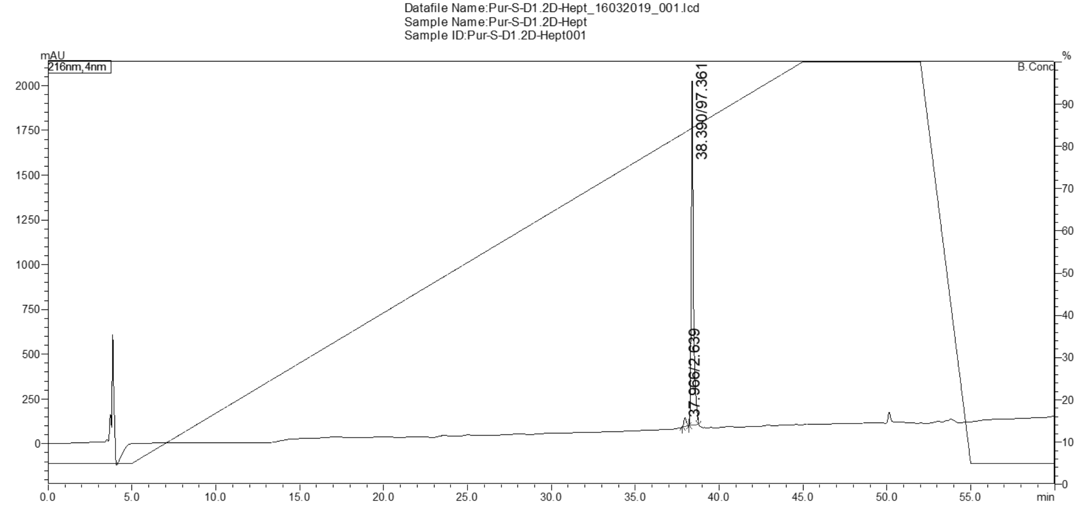

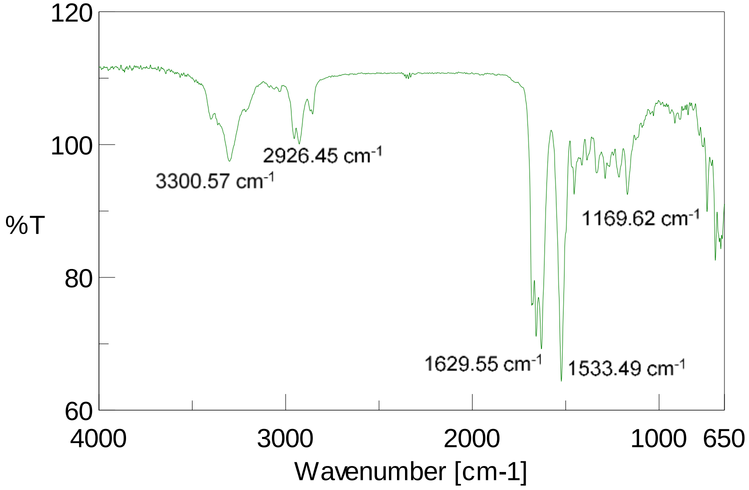

Characteristic fragmentation of compound **9f** and **9g**

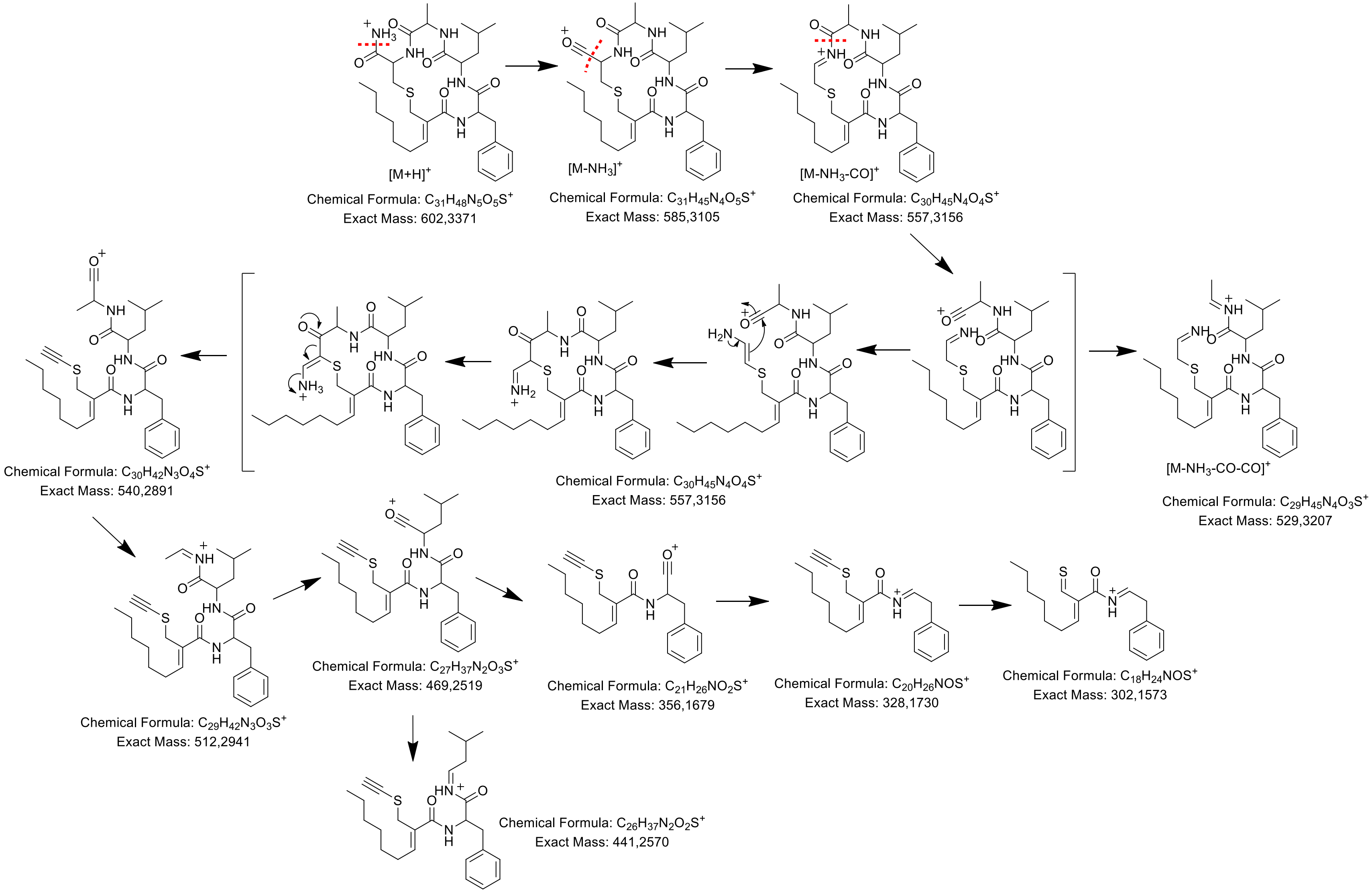

Characterization data for Compound 9h

^{1}H NMR Spectra of **9h** in DMSO-d_6

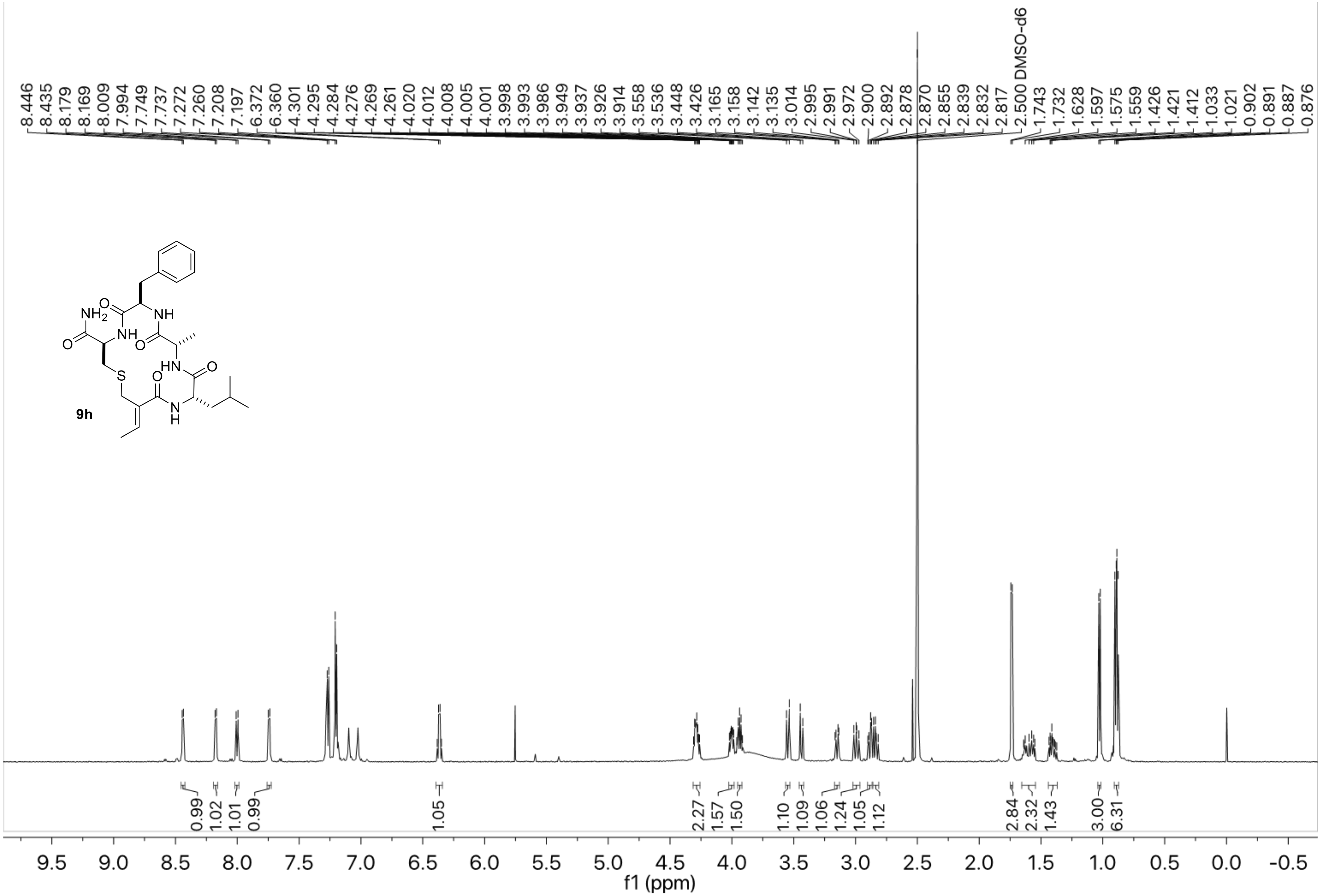

^{13}C NMR Spectra of **9h** in DMSO-d_6

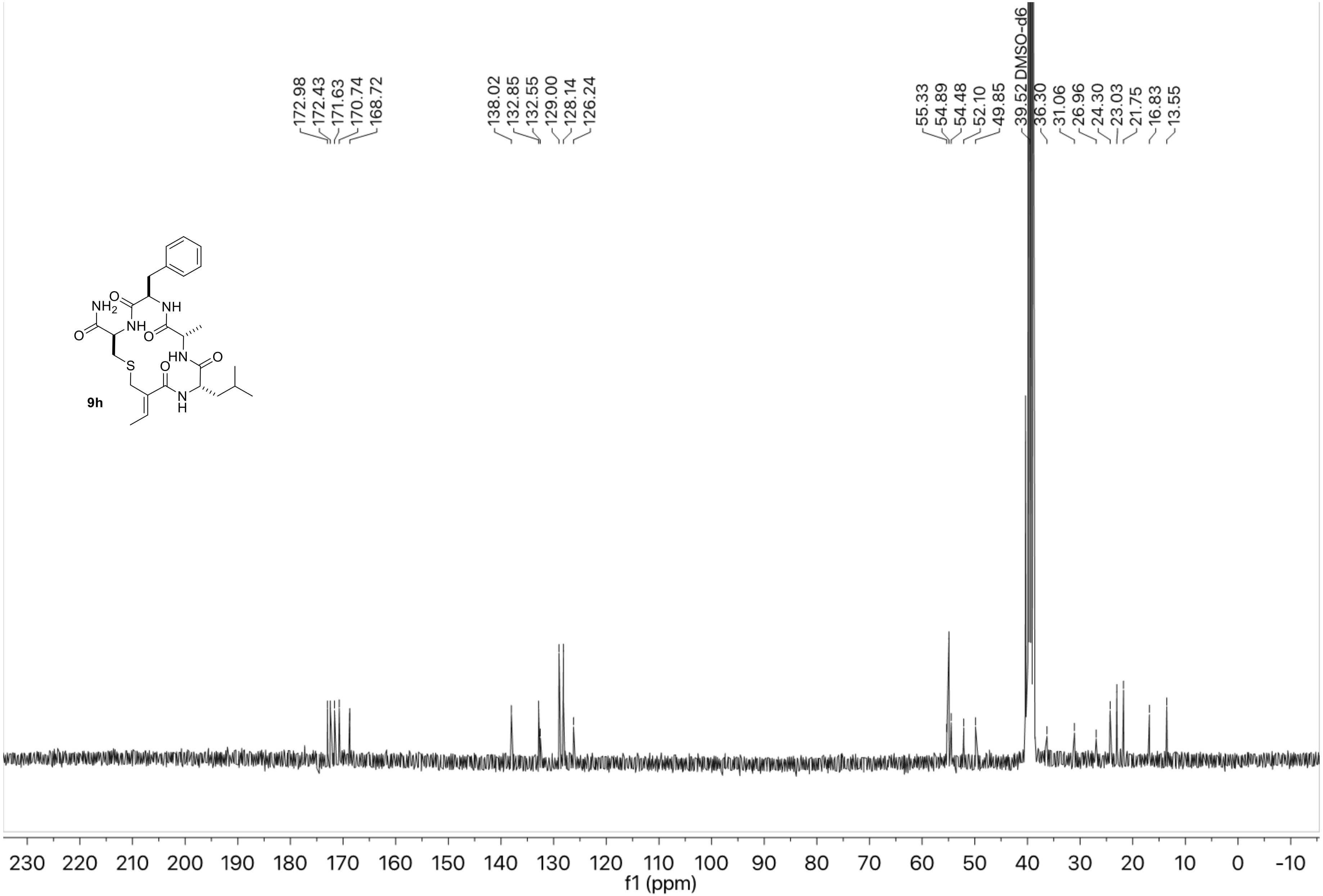

HSQC Spectra of **9h** in DMSO-d_6

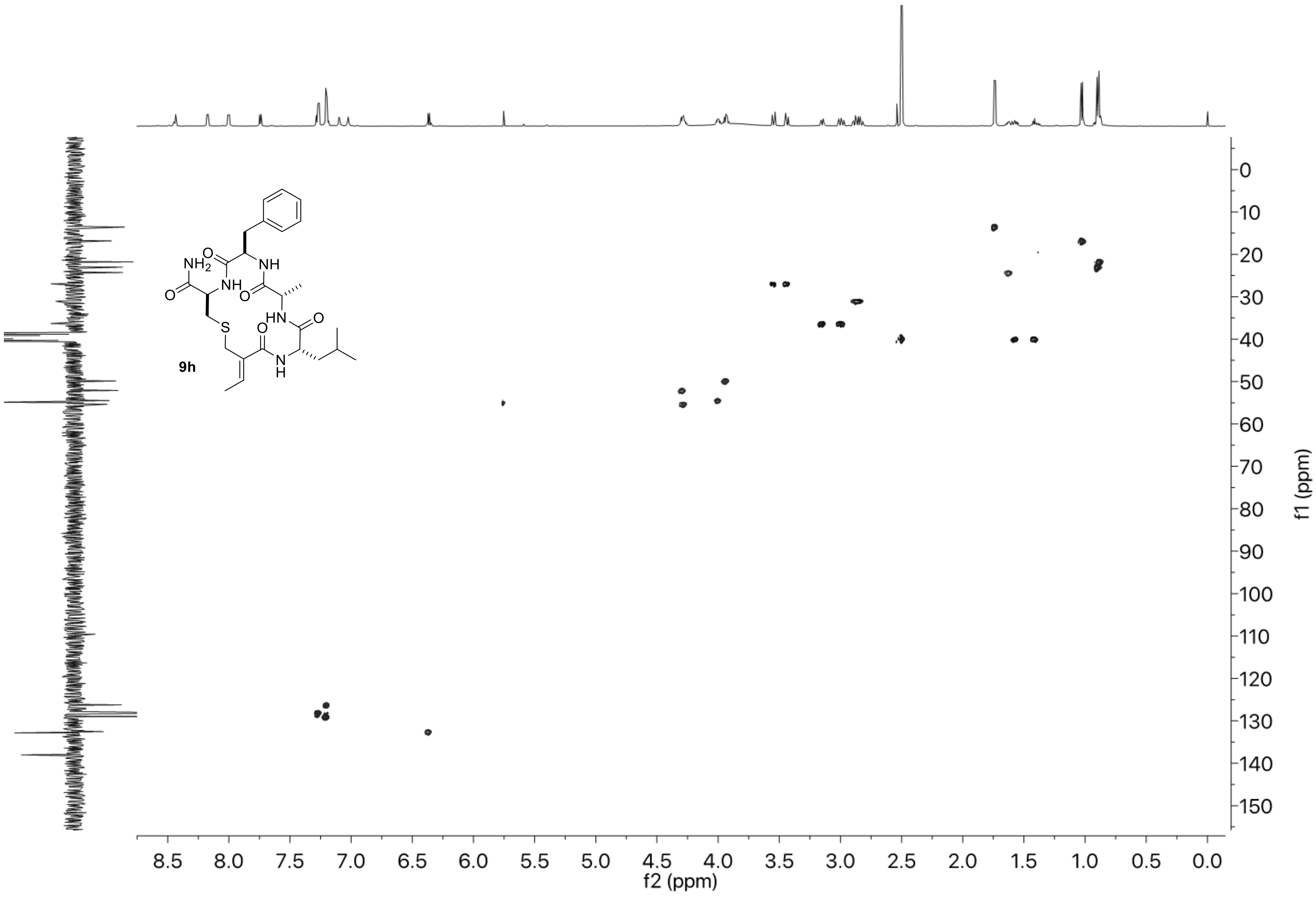

HMBC Spectra of **9h** in DMSO-d_6

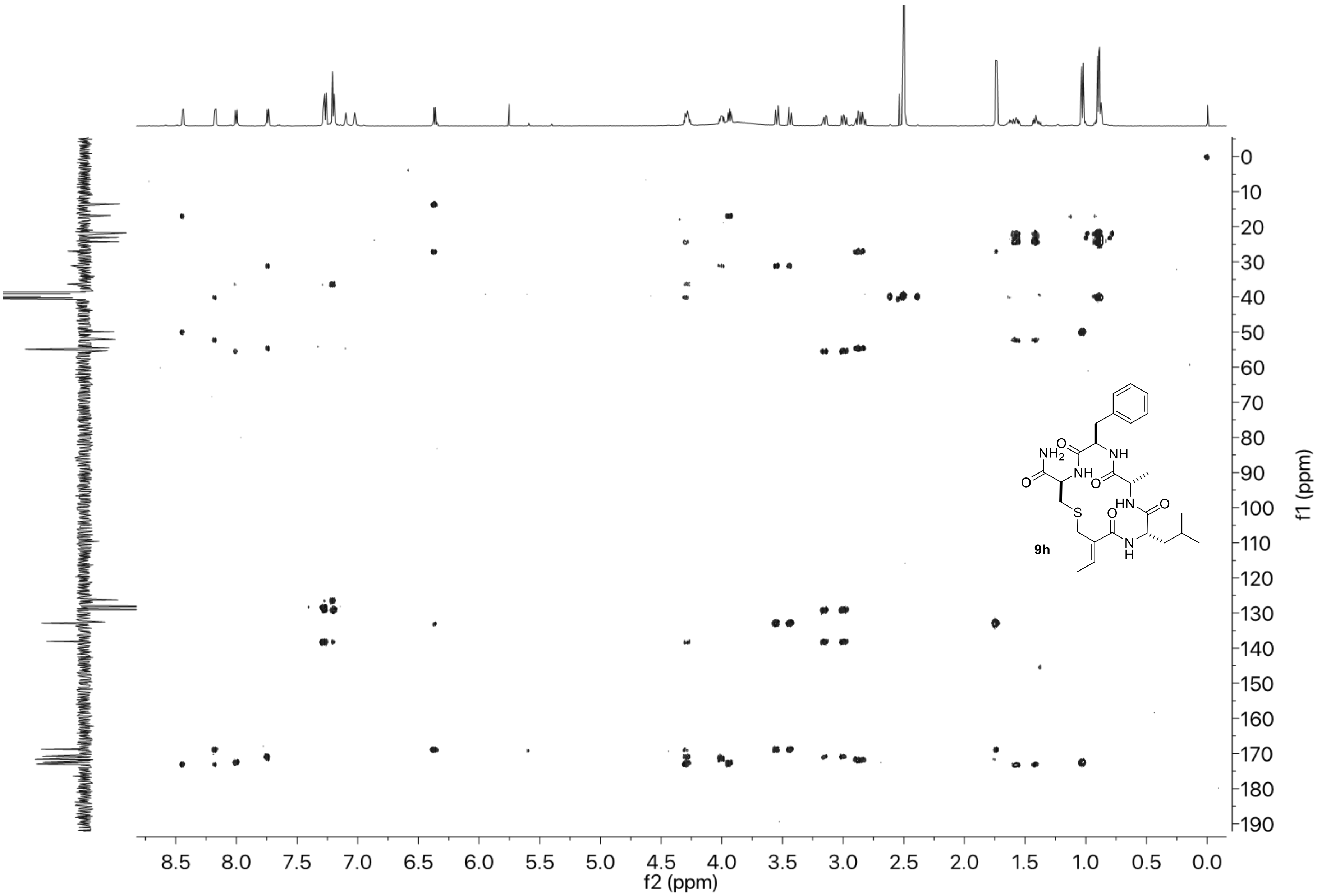

HRMS-ESI-TOF, HRMS/MS spectra, chromatogram and IR-ATR spectrum of **9h**

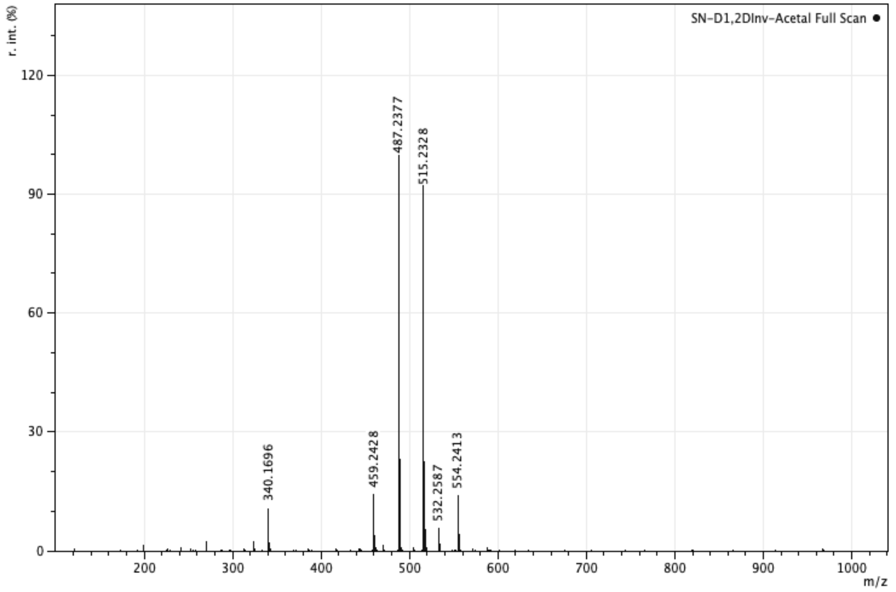

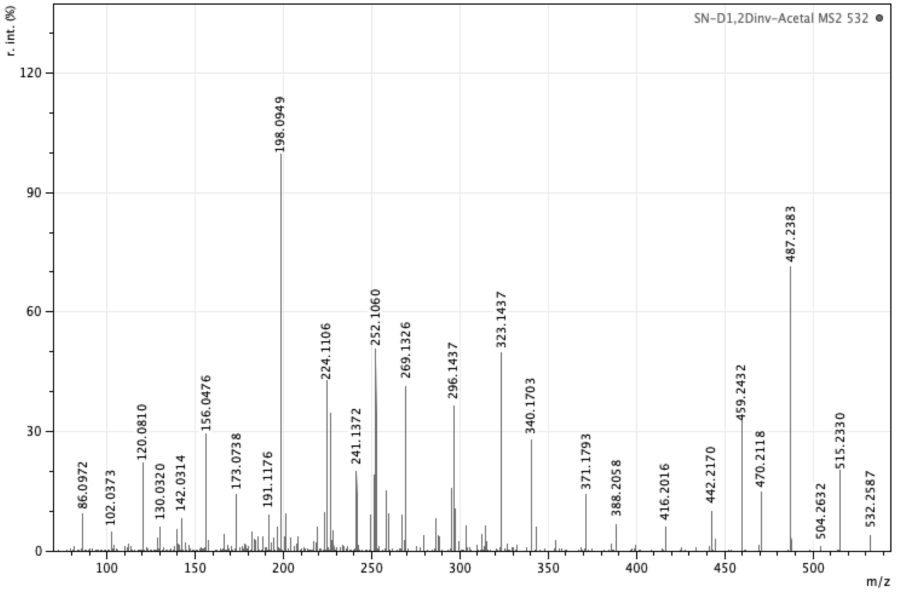

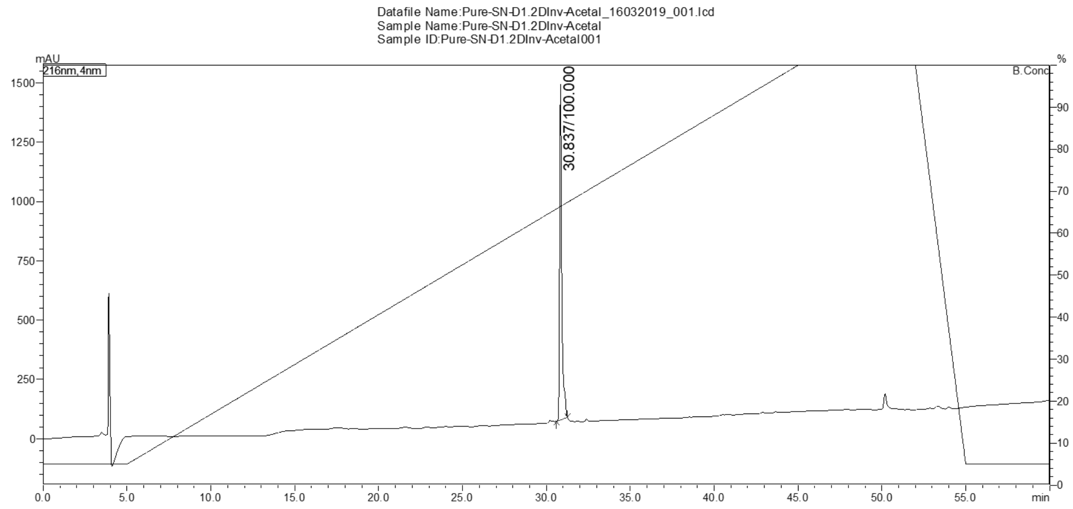

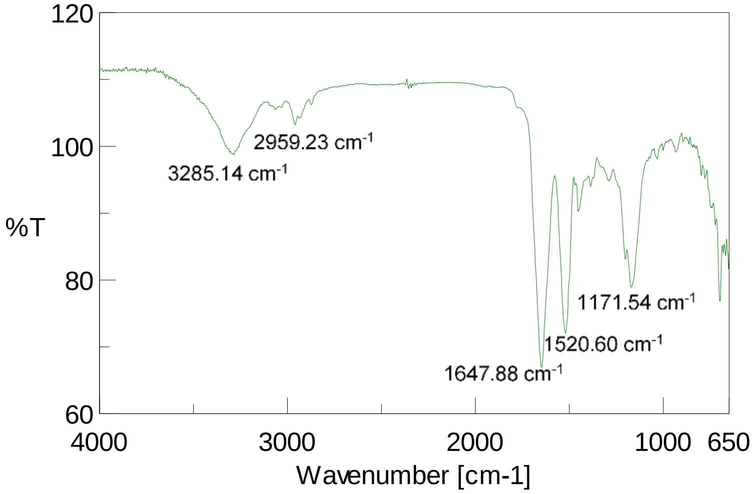

Characteristic fragmentation of compound **9h**

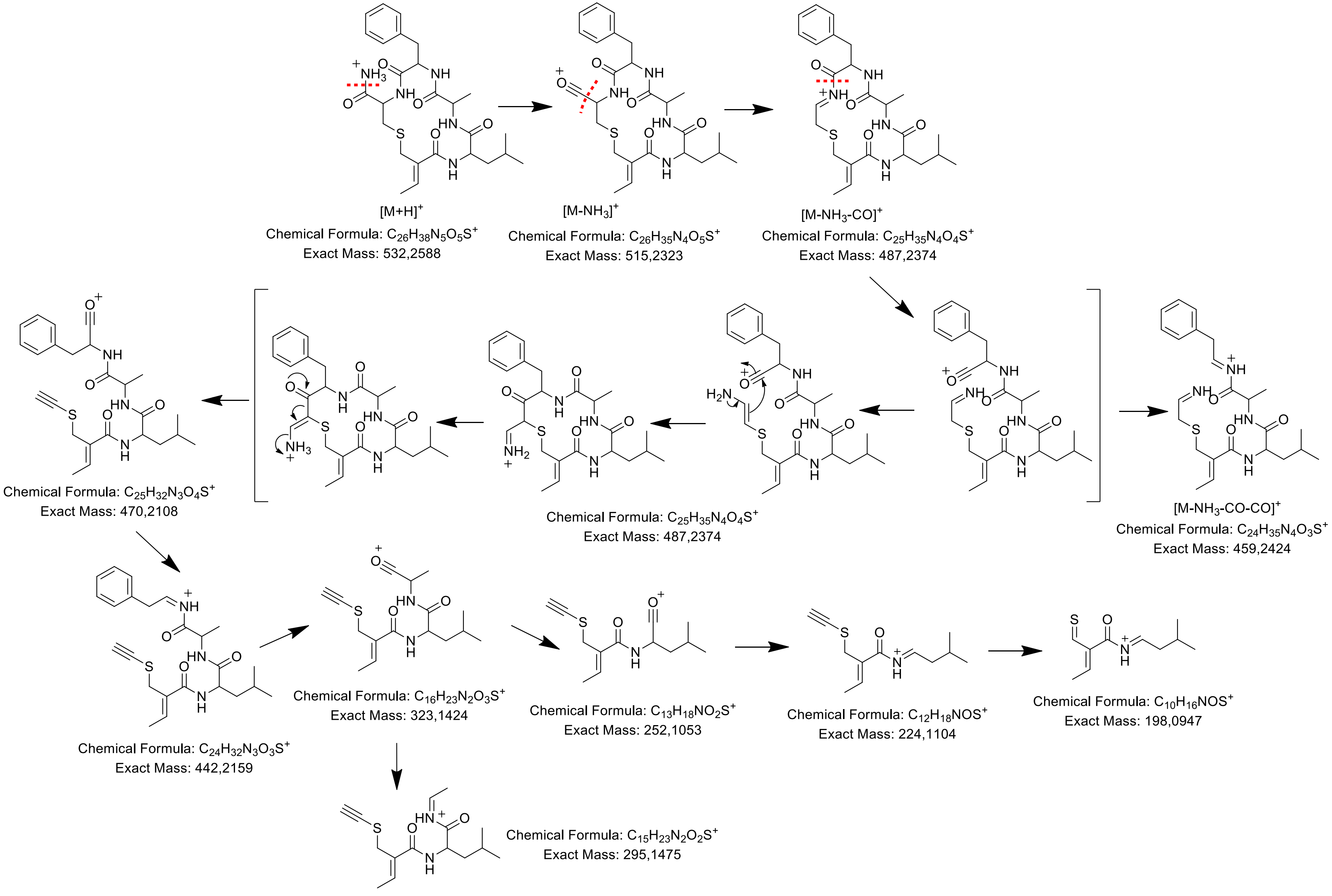

References

(1) mMass 5.5.0 (http://www.mmass.org)

(2) Clinical and Laboratory Standards Institute - CLSI. Methods for dilution antimicrobial susceptibility tests for bacteria that grow aerobically – ninth edition: approved Standard M7-A9. Wayne, PA, USA. 2012.

(3) Nakayama, J.; Uemura, Y.; Nishiguchi, K.; Yoshimura, N.; Igarashi, Y.; Sonomoto, K. *Antimicrob. Agents. Chemother.* **2009**, *53*, 580–586.

(4) Sakoulas, G.; Eliopoulos, G. M.; Moellering, R. C. Jr.; Wennersten, C.; Venkataraman, L.; Novick, R. P.; Gold, H. S.; *Antimicrob. Agents. Chemother.* **2002**, *46*, 1492–1502.

(5) Dos Santos, R. C.;Ombredane, A. S.; Souza, J. M. T.; Vasconcelos, A. G.; Plácido, A.; Amorim, A. D. G. N; Barbosa, E. A.; Lima, F. C. D. A.; Ropke, C. D.; Alves M. M. M.; Arcanjo, D. D. R.; Carvalho, F. A. A.; Delerue-Matos, C.; Joanitti, G. A.; Leite, J. R. S. A. *Food Res. Int.* **2018**, 105, 184-196.

(6) Silva, V. S.; Tolentino, T. A.; Rodrigues, T. C. A. F.; Santos, F. F. M.; Machado, D. F. S.; Silva, W. A.; de Oliveira, H. C. B.; Machado, A. H. L. *Org. Biomol. Chem.* **2019**, *17*, 4498–4511.

(7) Mateus, C. R.; Feltrin, M. P.; Costa, A. M.; Coelho, F.; Almeida, W. P. *Tetrahedron* **2001**, *57*, 6901–6908.

(8) Detterbeck, R.; Hesse, M. *Tetrahedron* **2002**, *58*, 6887–6893.

(9) Malapit, C. A.; Caldwell, D. R.; Sassu, N.; Milbin, S.; Howell, A. R. *Org. Lett.* **2017**, *19*, 1966–1969.
